# Parton distributions in the LHC era: MMHT 2014 PDFs

**DOI:** 10.1140/epjc/s10052-015-3397-6

**Published:** 2015-05-09

**Authors:** L. A. Harland-Lang, A. D. Martin, P. Motylinski, R. S. Thorne

**Affiliations:** Department of Physics and Astronomy, University College London, London, WC1E 6BT UK; Institute for Particle Physics Phenomenology, Durham University, Durham, DH1 3LE UK

## Abstract

We present LO, NLO and NNLO sets of parton distribution functions (PDFs) of the proton determined from global analyses of the available hard scattering data. These MMHT2014 PDFs supersede the ‘MSTW2008’ parton sets, but they are obtained within the same basic framework. We include a variety of new data sets, from the LHC, updated Tevatron data and the HERA combined H1 and ZEUS data on the total and charm structure functions. We also improve the theoretical framework of the previous analysis. These new PDFs are compared to the ‘MSTW2008’ parton sets. In most cases the PDFs, and the predictions, are within one standard deviation of those of MSTW2008. The major changes are the $u-d$ valence quark difference at small $x$ due to an improved parameterisation and, to a lesser extent, the strange quark PDF due to the effect of certain LHC data and a better treatment of the $D \rightarrow \mu$ branching ratio. We compare our MMHT PDF sets with those of other collaborations; in particular with the NNPDF3.0 sets, which are contemporary with the present analysis.

## Introduction

The parton distribution functions (PDFs) of the proton are determined from fits to the world data on deep inelastic and related hard scattering processes; see, for example, [1–6]. More than 5 years have elapsed since MSTW published [1] the results of their global PDF analysis entitled ‘Parton distributions for the LHC’. Since then there have been significant improvements in the data, including especially the measurements made at the LHC. It is therefore timely to present a new global PDF analysis within the MSTW framework, which we denote by MMHT2014.^1^

In the intervening period, the predictions of the MSTW partons have been compared with the new data as they have become available. The only significant shortcoming of these MSTW predictions was in the description of the lepton charge asymmetry from $W^\pm$ decays, as a function of the lepton rapidity. This was particularly clear in the asymmetry data measured at the LHC [9, 10]. This deficiency was investigated in detail in MMSTWW [11].^2^ In that work, fits with extended ‘Chebyshev’ parameterisations of the input distributions were carried out, to exactly the same data set as was used in the original global MSTW PDF analysis. To be specific, MMSTWW replaced the factors $(1\,+\,\epsilon x^{0.5}\,+\,\gamma x)$ in the MSTW valence, sea and gluon distributions by the Chebyshev polynomial forms $(1\,+\,\sum a_i T^\mathrm{Ch}_i(y))$ with $y=1-2\sqrt{x}$ and $i=1 \ldots 4$. The Chebyshev forms have the advantage that the parameters $a_i$ are well-behaved and, compared to the coefficients of the MSTW parameterisation, are rather small, with moduli usually $\le 1$. At the same time, MMSTWW [11] investigated the effect of also extending, and making more flexible, the ‘nuclear’ correction to the deuteron structure functions. The extended Chebyshev parameterisations resulted in an improved stability in the deuteron corrections. The main changes in the PDFs found in the ‘Chebyshev’ analysis, as compared to the MSTW fit, were in the valence up and down distributions, $u_V$ and $d_V$, for $x \lesssim 0.03$ at high $Q^2 \sim 10^4 ~\mathrm GeV^2$, or slightly higher $x$ at low $Q^2$; a region where there are weak constraints on the valence PDFs from the data used in these fits. These changes to the valence quark PDFs, essentially in the combination $u_V-d_V$, were sufficient to result in a good description of the data on lepton charge asymmetry from $W^\pm$ decays. Recall that the LHC data for the lepton asymmetry were not included in the MMSTWW [11] fit, but are predicted. There were no other signs of significant changes in the PDFs, and for the overwhelming majority of processes at the LHC (and the Tevatron) the MSTW predictions were found to be satisfactory; see [11] (though the precise shape of the $W,Z$ rapidity data was not ideal, particularly at NNLO) and e.g. [12, 13].

Nevertheless, it is time to take advantage of the new data in order to improve the precision of PDFs within the same general framework of the MSTW analysis. This includes a fit to new data from HERA, the Tevatron and the LHC, where the data have all been published by the beginning of 2014, which was chosen as a suitable cut-off point. It is worth noting at the beginning of the article that there are no very significant changes in the PDFs beyond those already in the MMSTWW set, and all predictions for LHC processes remain very similar to those for MMSTWW and in nearly all cases to MSTW2008. Despite the inclusion of new data there is a slight increase of PDF uncertainty in general (particularly for the strange quark) due to an improved understanding of the source of uncertainties. We also point out here that it is expected that there will be another update of the PDFs in the same framework with a time-scale consistent with the release of the final combination of HERA inclusive structure function data, more LHC data for a variety of processes, and also the expected availability of the full NNLO calculation of inclusive jet production and of top-quark pair production differential distributions.

The outline of the paper is as follows. In Sect. 2 we describe the improvements that we have in our theoretical procedures since the MSTW2008 analysis [1] was performed. In particular, we discuss the parameterisation of the input PDFs, as well as the improved treatments (i) of the deuteron and nuclear corrections, (ii) of the heavy flavour PDFs, (iii) of the experimental errors of the data and (iv) in fitting the neutrino-produced dimuon data. In Sect. 3 we discuss the non-LHC data which have been added since the MSTW2008 analysis, while Sect. 4 describes the LHC data that are now included in the fit, where we determine these by imposing a cut-off date of publication by the beginning of 2014. The latter section concentrates on the description of $W$ and $Z$ production data, together with a discussion of the inclusion of LHC jet production data.

**Fig. 1:**
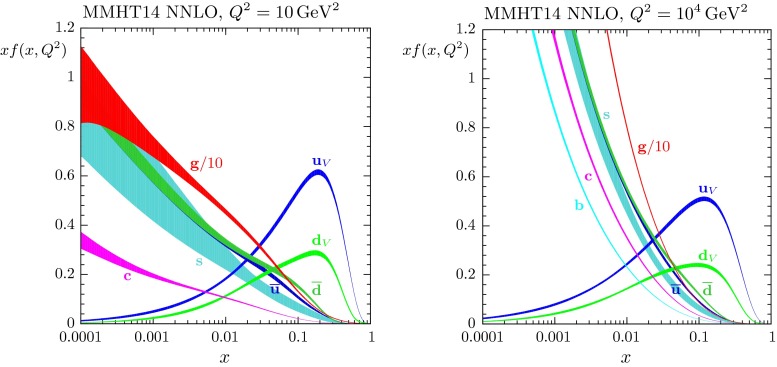
MMHT2014 NNLO PDFs at $Q^2=10 ~\mathrm GeV^2$ and $Q^2=10^4~\mathrm GeV^2$, with associated 68 $\%$ confidence-level uncertainty bands. The corresponding plot of NLO PDFs is shown in Fig. 20

The results of the global analysis can be found in Sect. 5. This section starts with a discussion of the treatment of the QCD coupling, and of whether or not to include $\alpha _S(M^2_Z)$ as a free parameter. We then present the LO, NLO and NNLO PDFs and their uncertainties, together with the values of the input parameters. These sets of PDFs are the end products of the analysis – the grids and interpolation code for the PDFs can be found at [14] and will be available at [15] and a new HepForge [16] project site is foreseen. An example is given in Fig. 1, which shows the NNLO PDFs at scales of $Q^2=10 ~\mathrm GeV^2$ and $Q^2=10^4 ~\mathrm GeV^2$, including the associated one-sigma (68 $\%$) confidence-level uncertainty bands.

Section 5 also contains a comparison of the NLO and NNLO PDFs with those of MSTW2008 [1]. The quality of the fit to the data at LO is far worse than that at NLO and NNLO, and is included for completeness, and because of the potential use in LO Monte Carlo generators, though the use of generators with NLO matrix elements is becoming far more standard. In Sect. 6 we make predictions for various benchmark processes at the LHC, and in Sect. 7 we discuss other data sets that are becoming available at the LHC which constrain the PDFs, but that are not included in the present global fit due to failure to satisfy our cut-off date; we refer to dijet and $W+c$ production and to the top quark differential distributions. In Sect. 8 we compare our MMHT PDFs with those of the very recent NNPDF3.0 analysis [17], and also with older sets of PDFs of other collaborations. In Sect. 9 we present our conclusions.

## Changes in the theoretical procedures

In this section, we list the changes in our theoretical description of the data, from that used in the MSTW analysis [1]. We also glance ahead to mention some of the main effects on the resulting PDFs.

### Input distributions 

As is clear from the discussion in the Introduction, one improvement is to use parameterisations for the input distributions based on Chebyshev polynomials. Following the detailed study in [11], we take for most PDFs a parameterisation of the form

1 $$\begin{aligned} xf(x,Q_0^2)~=~A(1-x)^\eta x^\delta \left( 1+\sum ^n_{i=1} a_i T^\mathrm{Ch}_i(y(x)) \right) , \end{aligned}$$

where $Q_0^2=1~\mathrm GeV^2$ is the input scale and the $T^\mathrm{Ch}_i(y)$ are Chebyshev polynomials in $y$, with $y=1-2x^k$, where we take $k=0.5$ and $n=4$. The global fit determines the values of the set of parameters $A,~\delta ,~\eta ,~a_i$ for each PDF, namely for $f=u_V,~d_V,~ S,~ s_+$, where $S$ is the light-quark sea distribution

2 $$\begin{aligned} S~\equiv ~2(\bar{u}+\bar{d})+s+\bar{s}. \end{aligned}$$

For $s_+\equiv s+\bar{s}$ we set $\delta _+=\delta _S$. As argued in [1] the sea quarks at very low $x$ are governed almost entirely by perturbative evolution, which is flavour independent, and any difference in the shape at very low $x$ is very quickly washed out. Hence, we choose to assume that this universality in the very low $x$ shape is already evident at input. For $s_+$ we also set the third and fourth Chebyshev polynomials to be the same as for the light sea, as there are not enough data which can constrain the strange quark, while leaving all four parameters in the polynomial free leads to instabilities.

We still have to specify the parameterisations of the gluon and of the differences $\bar{d}-\bar{u}$ and $s-\bar{s}$. For the parameterisation of $\Delta \equiv \bar{d}-\bar{u}$ we set $\eta _\Delta =\eta _S+2$, and we use the parameterisation

3 $$\begin{aligned} x\Delta (x,Q_0^2)~=~A_\Delta (1-x)^{\eta _{\Delta }} x^{\delta _{\Delta }} ( 1+ \gamma _\Delta x + \epsilon _\Delta x^2 ). \end{aligned}$$

The (poorly determined) strange quark difference is taken to have a simpler input form than that in (1). That is,

4 $$\begin{aligned} s_- ~\equiv ~ x(s-\bar{s}) ~=~A_- (1-x)^{\eta _{-}} x^{\delta _{-}} (1-x/x_0) \end{aligned}$$

where $A_-,~\delta _-$ and $\eta _-$ are treated as free parameters, and where the final factor in (4) allows us to satisfy the third number sum rule given in (6) below, i.e. $x_0$ is a crossing point. Finally, it was found long ago [18] that the global fit was considerably improved by allowing the gluon distribution to have a second term with a different small $x$ power

5 $$\begin{aligned} xg(x,Q_0^2)= & {} A_g(1-x)^{\eta _g} x^{\delta _g} \left( 1+\sum ^2_{i=1} a_{g,i} T^\mathrm{Ch}_i(y(x)) \right) \nonumber \\&+\,A_{g'}(1-x)^{\eta _{g'}} x^{\delta _{g'}}, \end{aligned}$$

where $\eta _{g'}$ is quite large, and concentrates the effect of this term towards small $x$. This means the gluon has seven free parameters ($A_g$ being constrained by the momentum sum rule), which would be equivalent to using five Chebyshev polynomials if the second term were absent.

The choice $k=0.5$, giving $y=1-2\sqrt{x}$ in (1), was found to be preferable in the detailed study presented in [11]. It has the feature that it is equivalent to a polynomial in $\sqrt{x}$, the same as the default MSTW parameterisation. The half-integer separation of terms is consistent with the Regge motivation of the MSTW parameterisation. The optimum order of the Chebyshev polynomials used for the various PDFs is explored in the fit. It generally turns out to be $n=4$ or 5. The advantage of using a parameterisation based on Chebyshev polynomials is the stability and good convergence of the values found for the coefficients $a_i$.

The input PDFs are subject to three constraints from the number sum rules

6 $$\begin{aligned}&\int ^1_0 \mathrm{d}x ~u_V(x,Q^2_0)=2,\quad \int ^1_0 \mathrm{d}x ~{d}_V(x,Q^2_0)=1,\nonumber \\&\quad \int ^1_0 \mathrm{d}x ~(s(x,Q^2_0)-\bar{s}(x,Q^2_0))=0, \end{aligned}$$

together with the momentum sum rule

7 $$\begin{aligned}&\int ^1_0 \mathrm{d}x ~x[u_V(x,Q^2_0)+d_V(x,Q_0^2)+S(x,Q_0^2)\nonumber \\&\quad +\,g(x,Q^2_0)]~=~1. \end{aligned}$$

We use these four constraints to fix $A_g,~A_u,~A_d$ and $x_0$ in terms of the other parameters. In total there are 37 free (PDF) parameters in the optimum global fit, and there is also the strong coupling defined at the scale of the $Z$ boson mass $M_Z$, i.e. $\alpha _s(M_Z^2)$, which we allow to be free when determining the best fit. Checks have been performed on our procedure which show that there is extremely little sensitivity to variation in $Q_0^2$ for either the fit quality or the PDFs extracted.

### Deuteron corrections

It is still the case that we need deep inelastic data on deuteron targets [19–24] in order to fully separate the $u$ and $d$ distributions at moderate and large values of $x$. Thus we should consider the correction factor $c(x)$ to be applied to the deuteron data

8 $$\begin{aligned} F^d(x,Q^2)~=~c(x) [F^p(x,Q^2)+F^n(x,Q^2) ]/2, \end{aligned}$$

where we assume $c$ is independent of $Q^2$ and where $F^n$ is obtained from $F^p$ by swapping up and down quarks, and anti-quarks; that is, isospin asymmetry is assumed. In the MSTW analysis, motivated by [25], despite the fact that the fit included all the deuteron data present in this analysis, the theory was only corrected for shadowing for small values of $x$, with a linear form for $c$ with $c=0.985$ at $x=0.01$ and $c=1$ just above $x=0.1$; above this point it was assumed that $c=1$.

In Ref. [11] we studied the deuteron correction factor in detail. We introduced the following flexible parameterisation of $c(x)$, which allowed for the theoretical expectations of shadowing (but which also allowed the deuteron correction factor to be determined by the data):

9 $$\begin{aligned} c(x)~&=~(1+0.01N)~[1+0.01c_1 \mathrm{ln}^2(x_p/x)],\nonumber \\&\qquad x<x_p,\end{aligned}$$

10 $$\begin{aligned} c(x)~&=~(1+0.01N)~[1+0.01c_2 \mathrm{ln}^2(x/x_p)\nonumber \\&\qquad +\,0.01c_3\mathrm{ln}^{20}(x/x_p)], \quad x>x_p, \end{aligned}$$

where $x_p$ is a ‘pivot point’ at which the normalisation is $(1+0.01N)$. For $x<x_p$ there is freedom for $c(x)$ to increase or decrease smoothly depending on the sign of the parameter $c_1$. The same is true above $x=x_p$, but the very large power in the $c_3$ term is added to allow for the expected rapid increase of $c(x)$ as $x \rightarrow 1$ due to Fermi motion. If, as expected, there is shadowing at low $x$ and also a dip for high, but not too high, $x$ (that is, if both $c_1$ and $c_2$ are found to be negative), then $x_p$ is where $c(x)$ will be a maximum, as expected from antishadowing (provided $N>0$). If we fix the value of $x_p$, then the deuteron correction factor $c(x)$ is specified by the values of four parameters: the $c_i$ and $N$. In practice $x_p$ is chosen to be equal to $0.05$ at NLO, but a slightly smaller value of $x_p=0.03$ is marginally preferred at NNLO.

**Fig. 2:**
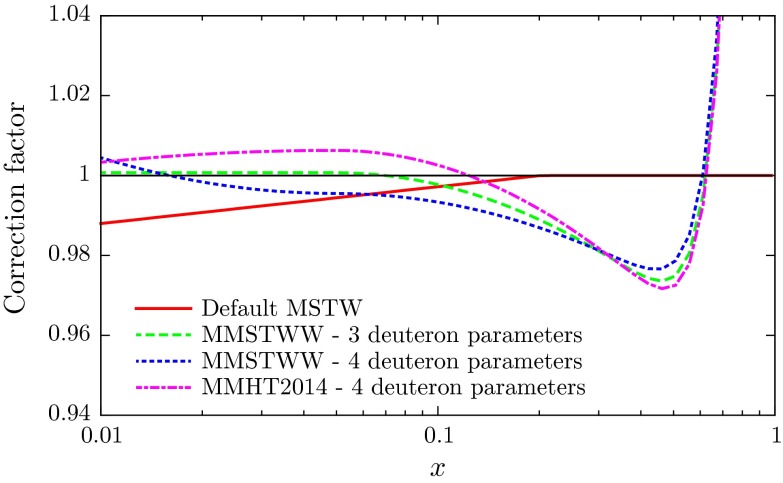
The deuteron correction factors versus $x$ at NLO shown for the fits listed in Table 1. The error corridor for the MMHT2014 curve is shown in Fig. 3, together with the result at NNLO

As already emphasised, the introduction of a flexible parameterisation of the deuteron correction, $c(x)$, coupled with the extended Chebyshev parameterisation of the input PDFs was found [11], unlike MSTW [1], to describe the data for lepton charge asymmetry from $W^\pm$ decays well, and, moreover, to give a much better description of the same set of global data as used in the MSTW analysis. The only blemish was that for the best possible fit the four-parameter version of $c(x)$ had an unphysical form (with $c_1$ positive), so the preferred fit, even though it was of slightly lower quality, was taken to be the three-parameter form with $c_1=0$. In the present analysis (which includes the post-MSTW data) this blemish does not occur, and the four-parameter form of the deuteron correction factor turns out to be much as expected theoretically. The parameters are listed in Table 1 and the corresponding deuteron correction factors shown in Fig. 2. The fit quality for the deuteron structure function data for MMSTWW at NLO with three parameters was 477/513, and it was just a couple lower when four parameters were used. For MMHT2014 at NLO the value is 471/513 and at NNLO is slightly better at 464/513. Hence, the new constraints on the flavour decomposition from the Tevatron and LHC are, if anything, slightly improving the fit to deuteron data, though part of the slight improvement is due to a small change in the way in which NMC data is used – see Sect. 2.7.

**Table 1:** The values of the parameters for the deuteron correction factor found in the MMSTWW [11] and the present (MMHT) global fits

PDF fit	$N$	$c_1$	$c_2$	$c_3\times 10^8$
MMSTWW, 3 pars.	0.070	0	$-0.608$	3.36
MMSTWW, 4 pars.	$-0.490$	0.349	$-0.444$	3.40
MMHT2014 NLO	$0.630 \pm 0.831$	$-0.116 \pm 0.507$	$-0.758 \pm 0.324$	$3.44 \pm 1.89$
MMHT2014 NNLO	$0.589 \pm 0.738$	$-0.116 \pm 0.996$	$-0.384 \pm 0.182$	$0.0489\pm 0.0056$

The uncertainties for the parameters in the MMHT2014 PDF fits are also shown in Table 1. These values are quoted as three times the uncertainty obtained using the standard $\Delta \chi ^2=1$ rule. In practice we use the so-called “dynamic tolerance” procedure to determine $\Delta \chi ^2$ for each of our eigenvectors, as explained in Section 6 of [1], and also discussed in Sect. 5 of this article, and a precise determination of the deuteron correction uncertainty is only obtained from the similar scan over $\chi ^2$ as used to determine eigenvector uncertainties. However, a typical value is three times the $\Delta \chi ^2=1$ uncertainty, and this should give a fairly accurate representation of the deuterium correction uncertainty.^3^ The correlation matrices for the deuteron parameters for the NLO and NNLO analyses are, respectively,

11 $$\begin{aligned}&c_{ij}^\mathrm{NLO} = \begin{pmatrix} 1.000 &{}\quad -0.604 &{}\quad -0.693 &{}\quad 0.177 \\ -0.604 &{}\quad 1.000 &{}\quad 0.426 &{}\quad -0.116 \\ -0.693 &{}\quad 0.426 &{}\quad 1.000 &{}\quad -0.360 \\ 0.177 &{}\quad -0.116 &{}\quad -0.360 &{}\quad 1.000 \\ \end{pmatrix},\end{aligned}$$

12 $$\begin{aligned}&c_{ij}^\mathrm{NNLO} = \begin{pmatrix} 1.000 &{}\quad -0.540 &{} \quad -0.692 &{}\quad 0.179 \\ -0.540 &{}\quad 1.000 &{}\quad 0.371 &{}\quad -0.118 \\ -0.692 &{}\quad 0.371 &{}\quad 1.000 &{}\quad -0.341 \\ 0.179 &{}\quad -0.118 &{}\quad -0.341 &{}\quad 1.000 \\ \end{pmatrix}. \end{aligned}$$

**Fig. 3:**
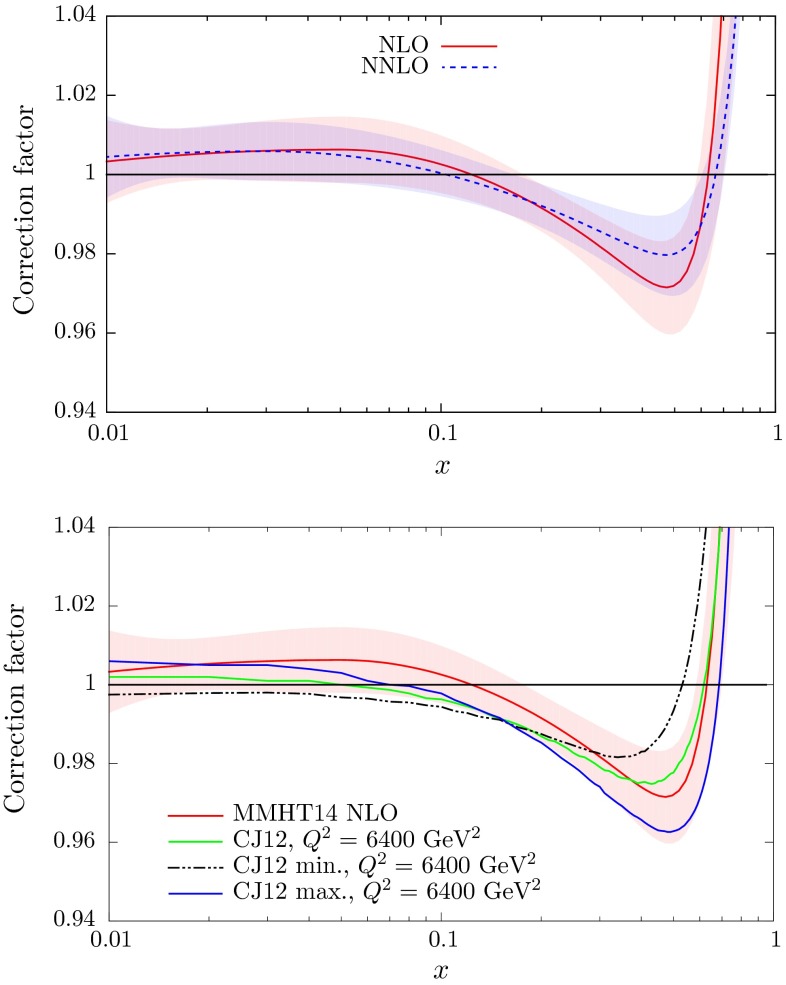
The deuteron correction factors versus $x$ at NLO and NNLO with uncertainties (*top*) and at NLO compared to the CJ12 corrections (*bottom*)

We plot the central values and uncertainties of the deuteron corrections at NLO and at NNLO in the higher plot of Fig. 3. One can see that the uncertainty is of order $1\,\%$ in the region $0.01\lesssim x\lesssim 0.4$ well constrained by deuteron data. Although the best fits now correspond to a decrease as $x$ becomes very small this is not determined within even a one standard deviation uncertainty band. The lack of deuteron data at high $x$, $x\gtrsim 0.75$, mean that the correction factor is not really well determined in this region, and the uncertainty is limited by the form of the parameterisation. However, the sharp upturn at $x \sim 0.6$ is driven by the data.

Until recently, most of the other groups that have performed global PDF analyses do not include deuteron corrections. An exception is the analysis of Ref. [27]. In the present work, and in MMSTWW [11], we have allowed the data to determine what the deuteron correction should be, with an uncertainty determined by the quality of the fit. The CTEQ-Jefferson Lab collaboration [27] have performed three NLO global analyses which differ in the size of the deuteron corrections. They are denoted CJ12min, CJ12med and CJ12max, depending on whether they have mild, medium or strong deuteron corrections. We plot the comparison of these to our NLO deuteron corrections in the lower plot of Fig. 3. The CJ12 corrections are $Q^2$-dependent due to target mass and higher-twist contributions, as discussed in [28]. These contributions die away asymptotically, so we compare to the CJ12 deuteron corrections quoted at a very high $Q^2$ value of $6400~\mathrm GeV^2$. In the present analysis it turns out that the data select deuteron corrections that are in very good agreement for $x>0.2$ with those given by the central CJ set, CJ12med. The behaviour at smaller values of $x$ is sensitive to the lepton charge asymmetry data from $W^\pm$ decays at the Tevatron and LHC, the latter of which are not included in the CJ12 fits.

### Nuclear corrections for neutrino data

The neutrino structure function data are obtained by scattering on a heavy-nuclear target. The NuTeV experiment [29] uses an iron target, and the CHORUS experiment [30] scatters on lead. Additionally the dimuon data from CCFR/NuTeV [31] is also obtained from (anti)neutrino scattering from an iron target. In the MSTW analysis [1] we applied the nuclear corrections $R_f$, defined as

13 $$\begin{aligned} f^A(x,Q^2)~=~R_f(x,Q^2,A)~f(x,Q^2), \end{aligned}$$

separately for each parton flavour $f$ using the results of a NLO fit by de Florian and Sassot [32]. The $f^A$ are defined to be the PDFs of a *proton* bound in a nucleus of mass number $A$. In the present analysis we use the updated results of de Florian et al., which are shown in Fig. 14 of [33]. The nuclear corrections for the heavy flavour quarks are assumed to be the same as that found for strange quarks, though the contribution from heavy quarks is very small. The updated nuclear corrections are quite similar, except for the strange quark for $x<0.1$, though this does not significantly affect the extracted values of the strange quark. The new corrections improve the quality of the fit by $\sim$25 units in $\chi ^2$, spread over a variety of data sets, including obvious candidates such as NuTeV $F_2(x,Q^2)$, but also HERA structure function data and CDF jet data which are only indirectly affected by nuclear corrections.

As in [1] we multiply the nuclear corrections by a three-parameter modification function, Eq. (73) in [1], which allows a penalty-free change in the details of the normalisation and shape. As in [1] the free parameters choose values $\lesssim 1$, i.e. they chose modification of only a couple of percent at most away from the default values. Hence, for both deuteron and heavy-nuclear corrections, we allow the fit to choose the final corrections with no penalty; but in both cases the corrections are fully consistent with expectation, i.e. any penalty applied would have very little effect.

### General mass – variable flavour number scheme (GM-VFNS)

The treatment of heavy flavours – charm, bottom – has an important impact on the PDFs extracted from the global analysis due to the data available for $F_2^h(x,Q^2)$ with $h=c,~b$, and also on the heavy flavour contribution to the total structure function at small $x$. Recall that there are two distinct regions where heavy quark production can be readily described. For $Q^2\sim m^2_h$ the massive quark may be regarded as being only produced in the final state, while for $Q^2 \gg m^2_h$ the quark can be treated as massless, with the ln$(Q^2/m^2_h)$ contributions being summed via the evolution equations. The GM-VFNS is the appropriate way to interpolate between these two regions, and as shown recently [34–36], the use of the fixed-flavour number scheme (FFNS) leads to significantly different results in a PDF fit to the GM-VFNS, even at NNLO. However, there is freedom to define different definitions of a GM-VFNS, which has resulted in the existence of various prescriptions, each with a particular reason for its choice. Well-known examples are the original Aivazis–Collins–Olness–Tung (ACOT) [37] and Thorne–Roberts (TR) [38] schemes, and their more recent refinements [39–41]. The MSTW analysis [1] adopted the more recent TR’ prescription in [41].

Ideally one would like any GM-VFNS to reduce exactly to the correct fixed-flavour number scheme at low $Q^2$ and to the correct zero-mass VFNS as $Q^2 \rightarrow \infty$. This has been accomplished in [34], by introducing a new ‘optimal’ scheme which improves the smoothness of the transition region where the number of active flavours is increased by one. The optimal scheme is adopted in the present global analysis.^4^

In general, at NLO, the PDFs, and the predictions using them can vary by as much as 2 $\%$ from the mean value due to the ambiguity in the choice of the GM-VFNS, and a similar size variation feeds into predictions for e.g. $W,Z$ and Higgs boson production at colliders. At NNLO there is far more stability to varying the GM-VFNS definition. Typical changes are less than 1 $\%$, and then only at very small $x$ values. This is illustrated well by the plots shown in Fig. 6 of [34]. Similarly predictions for standard cross sections vary at the sub-percent level at NNLO.

### Treatment of the uncertainties

All data sets which are common to the MSTW2008 and the present analysis are treated in the same manner in both, except that the multiplicative, rather than additive, definition of correlated uncertainties is used, as discussed in more detail below. All new data sets use the full treatment of correlated uncertainties, if these are available. For some data sets these are provided as a set of individual sources of correlated uncertainty, while for others only the final correlation matrix is provided.

If only the final correlation matrix is provided, then we use the expression

14 $$\begin{aligned} \chi ^2 = \sum _{i=1}^{N_\mathrm{pts}} \sum _{i=j}^{N_\mathrm{pts}} (D_i-T_i) (C^{-1})_{ij} (D_j-T_j), \end{aligned}$$

where $D_i$ are the data values $T_i$ are the parametrised^5^ predictions, and $C_{ij}$ is the covariance matrix.

In the case where the individual sources of correlated errors are provided the goodness-of-fit, $\chi ^2$, including the full correlated error information, is defined as

15 $$\begin{aligned} \chi ^2=\sum _{i=1}^{N_\mathrm{pts}}\left( \frac{D_i+\sum _{k=1}^{N_\mathrm{corr}} r_k\sigma _{k,i}^\mathrm{corr}-T_i}{\sigma _i^\mathrm{uncorr}}\right) ^2+\sum _{k=1}^{N_\mathrm{corr}}r_k^2, \end{aligned}$$

where $D_i+\sum _{k=1}^{N_\mathrm{corr}}r_k\sigma _{k,i}^\mathrm{corr}$ are the data values allowed to shift by some multiple $r_k$ of the systematic error $\sigma _{k,i}^\mathrm{corr}$ in order to give the best fit, and where $T_i$ are the parameterised predictions. The last term on the right is the penalty for the shifts of data relative to theory for each source of correlated uncertainty. The errors are combined multiplicatively, that is, $\sigma _{k,i}^\mathrm{corr}= \beta _{k,i}^\mathrm{corr}T_i$, where $\beta _{k,i}^\mathrm{corr}$ are the percentage errors. Previously, in MSTW [1], the additive definition was employed for all but the normalisation uncertainty. That is, $\sigma _{k,i}^\mathrm{corr} = \beta _{k,i}^\mathrm{corr}D_i$ was used.

To appreciate the consequence of the change we can think of the shift of data relative to theory as being approximately given by

16 $$\begin{aligned} \sum _{k=1}^{N_\mathrm{corr}} r_k\sigma _{k,i}^\mathrm{corr} = \sum _{k=1}^{N_\mathrm{corr}}\beta _{k,i}^\mathrm{corr}D_i(T_i) \approx \delta f D_i(T_i), \end{aligned}$$

where $\delta f$ is the fractional shift in the data – this is exactly correct for a normalisation uncertainty.

Defining $1+\delta f = f$, effectively the difference between the additive and multiplicative use of errors is that

17 $$\begin{aligned} D_i \!+\! \sum _{k=1}^{N_\mathrm{corr}}\beta _{k,i}^\mathrm{corr}D_i \sim f*D_i \quad \hbox {or} \quad T_i \!-\! \sum _{k=1}^{N_\mathrm{corr}}\beta _{k,i}^\mathrm{corr}T_i \sim T_i/f.\nonumber \\ \end{aligned}$$

So for the additive definition the data are effectively rescaled by $f$ while for the multiplicative definition the theory is rescaled by $1/f$. This means that in the two cases the $\chi ^2$ definition behaves like

18 $$\begin{aligned}&\chi ^2\sim \left( \frac{f*D_i-T_i}{\sigma _i^\mathrm{uncorr}}\right) ^2 \quad \hbox {or} \quad \chi ^2\sim \left( \frac{D_i-T_i/f}{\sigma _i^\mathrm{uncorr}}\right) ^2 \nonumber \\&\quad = \left( \frac{f*D_i-T_i}{f*\sigma _i^\mathrm{uncorr}}\right) ^2. \end{aligned}$$

Hence, with our new choice, the uncorrelated errors effectively scale with the data, whereas with the previous additive definition the uncorrelated uncertainties remain constant as the data are rescaled. The additive definition can therefore lead to a tendency for the data to choose a small scaling $f$ to bring the data closer together and hence reduce the $\chi ^2$, as pointed out in [42] and discussed in [43]. Our previous treatment of uncertainties guarded against this for the most obvious case of normalisation uncertainty by using the multiplicative definition for this particular source. However, the same type of effect is possible in any relatively large systematic uncertainty which affects all data points with the same sign, e.g. the jet energy scale uncertainty, so the multiplicative definition is the safer choice and is recommended by many experiments.

The other change we make in our treatment of correlated uncertainties is that we now use the standard quadratic penalty in $\chi ^2$ for normalisation shifts, rather than the quartic penalty adopted in MSTW [1]. It is checked explicitly that this makes essentially no difference in NLO and NNLO fits, but there is a tendency for some data to normalise down in a LO fit. In some cases the quality of the fit at LO would be very poor without this freedom, though it could often be largely compensated by a change in renormalisation and/or factorisation scale away from the standard values.

### Fit to dimuon data

**Fig. 4:**
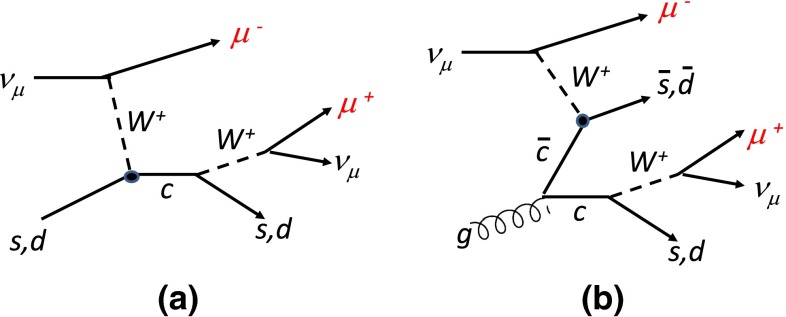
Diagrams for dimuon production in $\nu _{\mu }N$ scattering. Only diagram **a** was considered in [1], but here we include **b**, although it gives a very small contribution

Information on the $s$ and $\bar{s}$ quark distributions comes from dimuon production in $\nu _\mu N$ and ${\bar{\nu }}_\mu N$ scattering [31], where (up to Cabibbo mixing) an incoming muon (anti)neutrino scatters of a (anti)strange quark to produce a charm quark, which is detected via the decay of a charmed meson into a muon; see Fig. 4a. These data were included in the MSTW2008 analysis, but here we make two changes to the analysis, one far more significant, in practice, than the other.

#### Improved treatment of the $D \rightarrow \mu$ branching ratio, $B_{\mu }$

The comparison of theory predictions to the measured cross section on dimuon production requires knowledge of the branching fraction $B_\mu \equiv B(D\rightarrow \mu )$. In the previous analysis we used the fixed value $B_\mu =0.099$ obtained by the NuTeV collaboration itself [44]. However, this requires a simultaneous fit of the dimuon data and the branching ratio, which can be dependent on assumptions made in the analysis. Indeed, in studies for this article we have noticed a significant dependence on the parameterisation used for the input strange quark and the order of perturbative QCD used. Hence, in the present analysis, we avoid using information on $B_\mu$ obtained from dimuon data. Instead we use the value obtained from direct measurements [45]: $B_\mu = 0.092\pm 10\,\%$, where we feed the uncertainty into the PDF analysis. We note that this is somewhat lower than the number used in our previous analysis, though the two are easily consistent within the uncertainty of this value. We find that the fits prefer

19 $$\begin{aligned} B_\mu =(0.085-0.091) \pm 15\,\%, \end{aligned}$$

where the variation in the first number is the variation between the best value from different fits, and the uncertainty of $15\,\%$ is the uncertainty within any one fit due to the uncertainty on the data, i.e. the variation that provides a significant deterioration in $\chi ^2$ for dimuon data as determined by the dynamical tolerance procedure used to define PDF uncertainties. Hence, the preferred value is always close to the central value in [45]. These lower branching ratios compared to the MSTW2008 analysis lead to a small increase in the normalisation of the strange quark. However, probably more importantly, the large uncertainty on the branching ratio allows for a much larger uncertainty on the strange quark than in our previous analysis. Indeed, this is one of the most significant differences between MMHT2014 and MSTW2008 PDFs.

#### Inclusion of the $g\rightarrow c\bar{c}$ initiated process with a displaced vertex

We also correct the dimuon cross sections for a small missing contribution. In the previous analysis we calculated the dimuon cross section ignoring the contribution where the charm quark is produced away from the interaction point of the quark with the $W$ boson, i.e. the contributions where $g \rightarrow c \bar{c}$ then $(\bar{c})c + W^{\pm } \rightarrow (\bar{s})s$, as sketched in Fig. 4b. Previously we had included only Fig. 4a and had (incorrectly) assumed that the absence of Fig. 4b was accounted for by the acceptance corrections. We now include this type of contribution, but it is usually of the order $5\,\%$ or less of the total dimuon cross section. The correction to each of the structure functions, $F_2, F_L$ and $F_3$, is proportionally larger than this, but if we look at the total dimuon cross section then it is proportional to $s +(1-y)^2 \bar{c}$ (or $\bar{s} +(1-y)^2 c$), where $y$ is the inelasticity $y=Q^2/(xs)$ and $c (\bar{c})$ is the charm distribution coming from the gluon splitting. However, $c (\bar{c})$ only becomes significant compared to $s(\bar{s})$ at higher $Q^2$ and low $x$, exactly where $y$ is large and the charm contribution in the total cross section is suppressed. As such, this correction has a very small effect on the strange quark distributions that are obtained, being of the same order as the change in nuclear corrections and much smaller than the changes due to the different treatment of the branching ratio $B_{\mu }$.

### Fit to NMC structure function data

In the MSTW2008 fit we used the NMC structure function data with the $F_2(x,Q^2)$ values corrected for $R= F_L/(F_2-F_L)$ measured by the experiment, as originally recommended. However, it was pointed out in [46] that $R_\mathrm{NMC}$, the value of $R$ extracted from data by the NMC collaboration [20], was used more widely than was really applicable. For example it was applied without changing the value over a range of $Q^2$, and it was also often rather different from the prediction for $R$ obtained using the PDFs and perturbative QCD. In Section 5 of [47] we agreed with this, and showed the effect of using instead $R_{1990}$, a $Q^2$-dependent empirical parameterisation of SLAC data dating from 1990 [24] which agrees fairly well with the QCD predictions in the range where data are used. It was shown that the effect of this change on our extracted PDFs and value of $\alpha _S(M_Z^2)$ was very small (in contradiction to the claims in [46] but broadly in agreement with [48]), since the change in $F_2(x,Q^2)$ was only at most about the size of the uncertainty of a data point for a small fraction of the data points, and negligible for many data points. In this analysis we use the same treatment as in [47], i.e. the NMC structure data on $F_2(x,Q^2)$ with the $F_L(x,Q^2)$ correction very close to the theoretical $F_L(x,Q^2)$ value. This has very little effect, though the change in $F_2^d(x,Q^2)$ for $x<0.1$ does help the deuteron correction at low $x$ to be more like the theoretical expectation.

## Non-LHC data included since the MSTW2008 analysis

Here we list the changes and additions to the non-LHC data sets used in the present analysis as compared to MSTW2008 [1]. All the data sets used in the MSTW2008 analysis are still included, unless the update is explicitly mentioned below. We continue to use the same cuts on structure function data, i.e. $Q^2=2~\mathrm GeV^2$ and $W^2=15~\mathrm GeV^2$. In [1] we imposed a stronger $W^2=25~\mathrm GeV^2$ cut on $F_3(x,Q^2)$ structure function data due to the expected larger contribution from higher-twist corrections in $F_3(x,Q^2)$ than in $F_2(x,Q^2)$; see e.g. [49]. However, this still leaves a possible contribution from quite small $x$ values for rather low $Q^2$. Hence we now impose a cut on $Q^2 = 5~\mathrm GeV^2$ for $F_3(x,Q^2)$.

As an aside, we should comment on the very small $x$ domain. As usual we do not impose any cut at low $x$, although, at present, there are essentially no (non-LHC or LHC) data available probing the $x\lesssim 0.001$ domain.^6^ The present analysis is based entirely on fixed-order DGLAP evolution. So when we show plots, like Fig. 1 going down to $x=10^{-4}$, and, later, when we show comparison plots going down to $x=10^{-5}$, we are going well beyond the available data, and also entering a domain which is potentially beyond the validity of a pure DGLAP framework. One possible source of contamination is large higher-twist corrections. However, even assuming these are small, in principle, the very small $x$ physics is influenced by the presence of large $\ln (1/x)$ terms in the perturbative expansion, which can be obtained from solutions of the BFKL equation (though this can include some higher-twist information as well). When data constraints are available at very small $x$, it is arguably the case that a unified fixed-order and resummation approach should be implemented. In [57–59] splitting functions are derived in this approach, with good agreement between groups. These suggest that the resummation effects lower the splitting functions for $x \sim 0.001$–$0.0001$ before a rise at $x< 10^{-5}$, and the likely effect is a slight slowing of evolution at low $Q^2$ and $x$. Another related approach is to consider unified BFKL/DGLAP evolution which has been derived for the (integrated) gluon PDF in terms of the gluon emission opening angle [60].

Having discussed the kinematic cuts that we apply, we are now ready to discuss the fit obtained using only the non-LHC data sets. We study the inclusion of a variety of LHC data in the next section. We note that in the fits, performed in this section, the coefficients of all four Chebyshev polynomials for the $s_+$ distribution are set equal to those for the light sea, as without LHC data there is insufficient constraining power in the data to fit these independently. This makes a completely direct comparison between the full PDFs including LHC data in the analysis and the PDFs without LHC data impossible.

We replace the previously used HERA run I neutral and charged current data measured by the H1 and ZEUS collaborations, by their combined data set [61] and use the full treatment of correlated errors. We use a lower $Q^2$ cut of $2 ~\mathrm GeV^2$ and break the data down into five subsets; $\sigma ^{\mathrm{NC},e^+p}$ at centre-of-mass energy $820$ GeV (78 points), $\sigma ^{\mathrm{NC},e^+p}$ at centre-of-mass energy $920$ GeV (330 points), $\sigma ^{\mathrm{NC},e^-p}$ at centre-of-mass energy $920$ GeV (145 points), $\sigma ^{\mathrm{CC},e^+p}$ at centre-of-mass energy $920$ GeV (34 points) and $\sigma ^{\mathrm{NC},e^-p}$ at centre-of-mass energy $920$ GeV (34 points). The fit to these data is very good at both NLO and NNLO; with a slightly better fit at NNLO, i.e. $\chi ^2/N_\mathrm{pts}=644.2/621$ at NNLO compared to $666.0/621$ at NLO. Most of this improvement is in the $\sigma ^{\mathrm{NC},e^+p}$ data which is 16 units better at NNLO. We do not include the separate H1 and ZEUS run II data yet, but wait for the combined data set, which as for run I we anticipate will produce improved constraints compared to the separate sets.

Similarly, we remove the previous measurements by ZEUS and H1 of $F_2^{c\bar{c}}(c,Q^2)$ and include instead the combined HERA data on $F_c(x,Q^2)$ [62] and use the full information on correlated uncertainties. Unlike the inclusive structure function data these data are fit better at NLO than NNLO, with $\chi ^2/N_\mathrm{pts} = 68.5/52$ at NLO but $\chi ^2/N_\mathrm{pts} = 78.5/52$ at NNLO (this difference is less clear, and the values of $\chi ^2$ are lower, if the additive definition of correlated uncertainties is used for this data set). As in the MSTW2008 analysis we use $m_c=1.4~\mathrm GeV$ in the pole mass scheme. Preliminary investigation implies that if $m_c$ is varied, a value $1.2$–$1.3~\mathrm GeV$ is preferred at both NLO and NNLO.

**Fig. 5:**
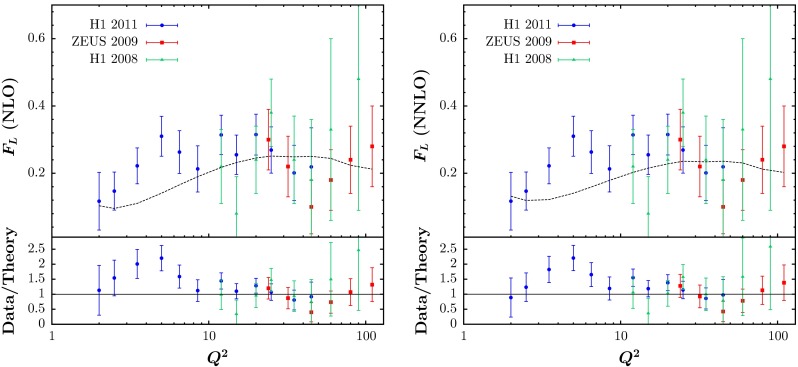
The fit quality for the HERA data on $F_L(x,Q^2)$ from [63–65] at NLO (*left*) and NNLO (*right*). The *dotted curve*, shown for illustration, is obtained from the prediction for the data in [64] below $Q^2=45~\mathrm GeV^2$ and from the prediction for the data in [65] above this. The “Data/Theory” comparison is obtained for the individual data points in each case

We also include all of the HERA $F_L(x,Q^2)$ measurements published before the beginning of 2014 [63–65]. The global fit undershoots some of the data a little at the lowest $Q^2$ values, slightly more so at NNLO than at NLO, as seen in Fig. 5, but the $\chi ^2$ values are not much more than one per point. For the HERA $F_L(x,Q^2)$ data we obtain $\chi ^2/N_\mathrm{pts}= 29.8/26$ at NLO and $\chi ^2/N_\mathrm{pts}= 30.4/26$ at NNLO.

**Fig. 6:**
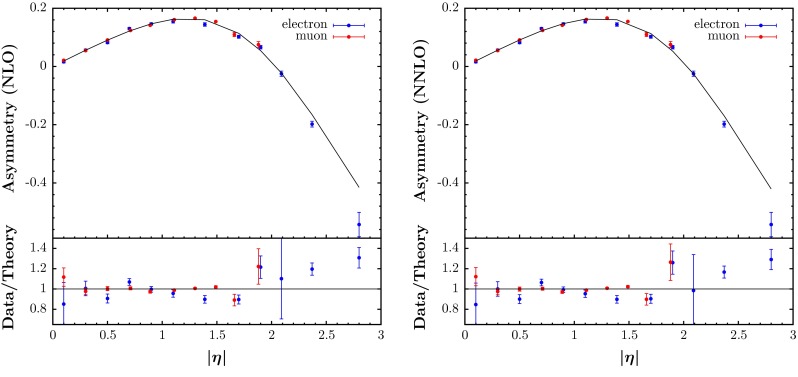
The fit quality for the two D0 lepton asymmetry data sets [67, 68] at NLO (*left*) and NNLO (*right*)

In the present analysis we include the CDF $W$ charge asymmetry data [66], the D0 electron charge asymmetry data with $p_T>25$ GeV based on 0.75 $\mathrm{fb}^{-1}$ [67] and the new D0 muon charge asymmetry data with $p_T>25$ GeV based on 7.3 $\mathrm{fb}^{-1}$ [68]. These replace the Tevatron asymmetry data used in the MSTW2008 analysis. Where the information on correlated uncertainties is available we use this in the conventional manner in calculating the $\chi ^2$ values. The nominal fit quality for each of these data sets appears quite poor with $\chi ^2/N_\mathrm{pts}= 32.1/13, 30.5/12$ and $20.3/10$, respectively, at NLO and $\chi ^2/N_\mathrm{pts}= 28.8/13, 28/12$ and $19.8/10$ respectively at NNLO, but this seems to be mainly due to fluctuations in the data making a very good quality fit impossible (especially when fitting the data sets simultaneously), as seen in Fig. 6. There is a tendency to overshoot the data at the very highest rapidity, though this is a little less at NNLO than at NLO (we use FEWZ [69] for the NLO and NNLO corrections).We do get an approximately 2 sigma shift of data relative to theory corresponding to the systematic uncertainty due to electron identification for the fit to CDF $W$ charge asymmetry data, but no large shifts for the new D0 muon charge asymmetry data.

We also include the final measurements for the CDF $Z$ rapidity distribution [70], since the final data changed slightly after the MSTW fit. We also now include the very small photon contribution in our calculation. The effect of this second correction was discussed in Section 11.2 of [1], although it was not used in the extraction of the MSTW2008 PDFs. The effect of both the final data set and the photon contribution is to improve the fits quality, $\chi ^2/N_\mathrm{pts}= 36.9/28$ at NLO and $39.6/28$ at NNLO, compared to $49/29$ at NLO and $50/29$ at NNLO in [1], while having essentially negligible impact on the PDFs.

These changes to the theoretical procedures, and additions to the global data that are fitted, do not change the PDFs very much from those in [1], except for the large change in ($u_V-d_V$) around $x \lesssim 0.01$, which was already found in [11]. The small changes can be seen in Figs. 21, 22, 23, 24 and 25 where we show the central values of these PDFs fit only to non-LHC data with the comparison of the MMHT2014 and MSTW2008 PDFs. There is a moderate reduction in the uncertainty on the very small $x$ gluon distribution due to the inclusion of the combined HERA data. Without the inclusion of the error on the branching ratio in dimuon production there is also a small improvement in the uncertainty on light quarks, but this is lost when the branching ratio uncertainty is included; as the increased uncertainty on the strange quarks also leads to some increase in the uncertainty of the up and down quarks. As seen in Fig. 13 of [11] the increased parameterisation and improved deuteron corrections lead to an increase in the uncertainty in the up and down valence quarks, and this is far from compensated for by the inclusion of the new non-LHC data in this analysis. There is also only a small shift in the value of the QCD coupling extracted in the best fit to data:

20 $$\begin{aligned} \mathrm{at \,\,NLO}\quad \alpha _S(M_Z^2)= & {} 0.1200 \quad \mathrm{from}\quad 0.1202 \end{aligned}$$

21 $$\begin{aligned} \mathrm{at \,\,NNLO} \quad \alpha _S(M_Z^2)= & {} 0.1181 \quad \mathrm{from}\quad 0.1171 \end{aligned}$$

## The LHC data included in the present fit

We now discuss the inclusion of the LHC data into the PDF fit. This includes a variety of data on $W$ and $Z$ production, also the completely new process for our PDF determination of top-quark pair production, and finally jet production. The addition of these LHC data sets to the data already discussed leads us to our final set of MMHT2014 PDFs. We make these PDFs available at NLO and NNLO, but also at LO. The full LO fit requires a much higher value of the strong coupling, $\alpha _S(M_Z^2)=0.135$, if the standard scale choices are made, i.e. $\mu ^2=Q^2$ in deep inelastic scattering, $\mu ^2 =M^2$ in Drell–Yan production and $\mu ^2=p_T^2$ in jet production, the same choices as made at NLO and NNLO. Even so the fit quality is much worse at LO than at NLO and NNLO, both of which give a similar quality of description of the global data. We will present full details of the fit quality and the PDFs in the next section, but first we present the results of the fit to each of the different types of LHC data.

### $W$ and $Z$ data

In order to include the LHC data on $W$ and $Z$ production in a variety of forms of differential distribution we use APPLGrid–MCFM [71–73] at NLO to produce grids which are interfaced to the fitting code, and at NNLO we use DYNNLO [74] and FEWZ [69] programs to produce precise $K$-factors (as a function of $\alpha _S$) to convert NLO to NNLO. In the vast majority of cases the NLO to NNLO conversion is a very small correction, especially for asymmetries and ratios.

The quality of the description of the LHC $W$ and $Z$ data in the present NLO and NNLO MMHT fits is shown in the last column of Table 2. For comparison, we also show the quality of the predictions of the MMHT fits and of the MMSTWW fits [11], neither of which included these, or any other, LHC data. We discuss the description of the data sets listed in Table 2 in turn.

**Table 2:** The quality of the description (as measured by the value of $\chi ^2$) of the LHC $W,Z$ data before and after they are included in the global NLO and NNLO fits. We also show for comparison the $\chi ^2$ values obtained in the CPdeut fit of the NLO MMSTWW analysis [11], which did not include LHC data

Data set	$N_\mathrm{pts}$	MMSTWW (Ref. [11])	MMHT2014 (no LHC)	MMHT2014 (with LHC)
NLO				
ATLAS $W^+, W^-, Z$	30	47	44	38
CMS $W$ asymm $p_T >35~\mathrm GeV$	11	9	16	7
CMS asymm $p_T >25~\mathrm GeV,30~\mathrm GeV$	24	9	17	8
LHCb $Z\rightarrow e^+e^-$	9	13	13	13
LHCb $W$ asymm $p_T >20~\mathrm GeV$	10	12	14	12
CMS $Z\rightarrow e^+e^-$	35	21	22	19
ATLAS high-mass Drell–Yan	13	20	20	21
CMS double-diff. Drell–Yan	132	385	396	372
NNLO				
ATLAS $W^+, W^-, Z$	30	72	53	39
CMS $W$ asymm $p_T >35~\mathrm GeV$	11	18	15	8
CMS asymm $p_T >25,30~\mathrm GeV$	24	18	17	9
LHCb $Z\rightarrow e^+e^-$	9	23	22	21
LHCb $W$ asymm $p_T >20~\mathrm GeV$	10	24	21	18
CMS $Z\rightarrow e^+e^-$	35	30	24	22
ATLAS high-mass Drell–Yan	13	18	16	17
CMS double-diff. Drell–Yan	132	159	151	150

#### ATLAS $W$ and $Z$ data

**Fig. 7:**
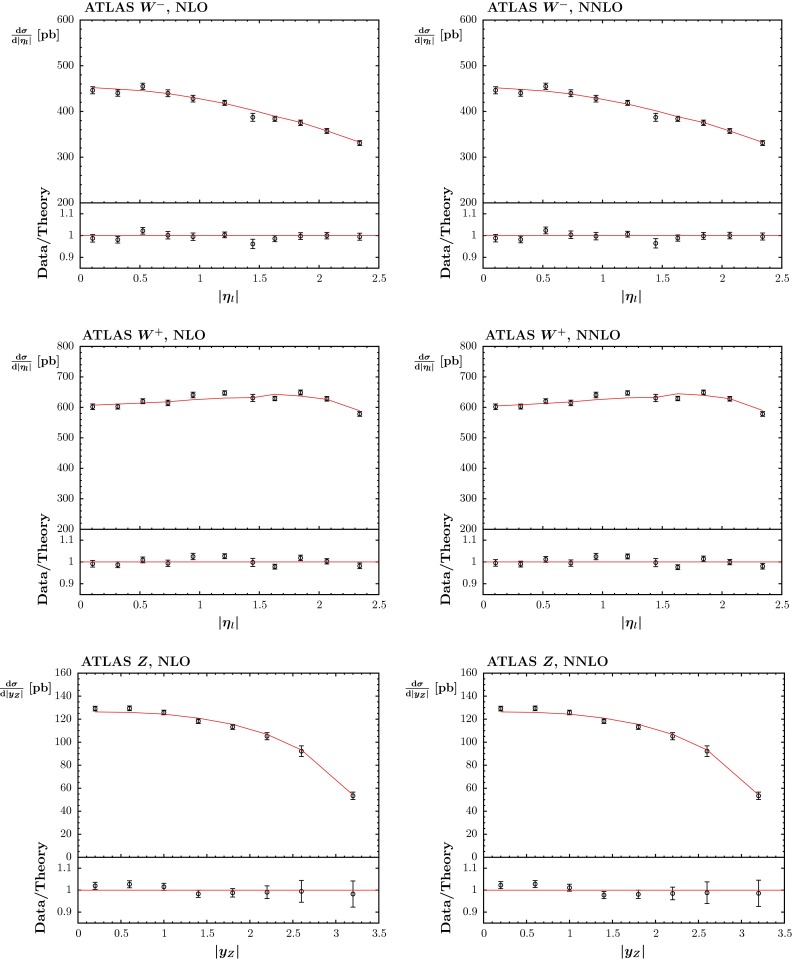
The fit quality of the ATLAS $W^-,W^+$ data sets for $\mathrm{d}\sigma /\mathrm{d}|\eta _l|$ (pb) versus $|\eta _l|$, and of the $Z$ data set for $\mathrm{d}\sigma /\mathrm{d}|y_Z|$ versus $|y_Z|$ [10], obtained in the NLO (*left*) and NNLO (*right*) analyses. The *points* shown are when the shift of data relative to the theory due to correlated systematics is included. However, this shift is small compared to the uncorrelated error for the data, so the comparison before shifts is not shown

First we consider the description of the ATLAS $W$ and $Z$ rapidity data [10]. These were poorly predicted by the MSTW2008 PDFs (see e.g. [75]), primarily due to the incorrect balance between $W^+$ and $W^-$ production at low rapidity, which is sensitive to the low-$x$ valence quark difference, and which shows up most clearly in the asymmetry between $W^+$ and $W^-$ production. This particular issue was automatically largely solved by the improved parameterisation and deuteron corrections in the MMSTWW study [11]. Nevertheless, we see from Table 2 that the quality of the description using the MMSTWW sets still has $\chi ^2 \sim 1.6$ per point for the NLO fit, and $\chi ^2 > 2$ per point in the NNLO fit. At NNLO it turns out that $u_V(x)-d_V(x)$ at small-$x$ is still not quite large enough to reproduce the observable charge asymmetry. However, at both NLO and NNLO the shape of the rapidity distribution (driven by the evolution of anti-quarks and hence ultimately by the gluon) is not quite ideal, and also a slightly larger fraction of strange quarks in the sea is preferred. The inclusion of the non-LHC data, together with the changes in theoretical procedure mentioned in Sect. 2 (not included in [11]), already improves the fit quality, particularly at NNLO, and after the inclusion of these ATLAS data, the $\chi ^2$ improves to about 1.3 per point at both NLO and NNLO. This appears to be not quite as good as the best possible fits to these data, which seem to require an even larger strange quark fraction in the sea; indeed, the same fraction as the up and down sea [76], or even larger (in the ‘collider-only’ fit in [3]). The fit quality is shown in Fig. 7. One can see that there is a slight tendency to undershoot the $Z$ data at the lowest rapidity, which could be improved by a slight increase in the strange distribution for $x \sim 0.01$, as seen in [76], but also verified in our studies.

#### CMS asymmetry data

**Fig. 8:**
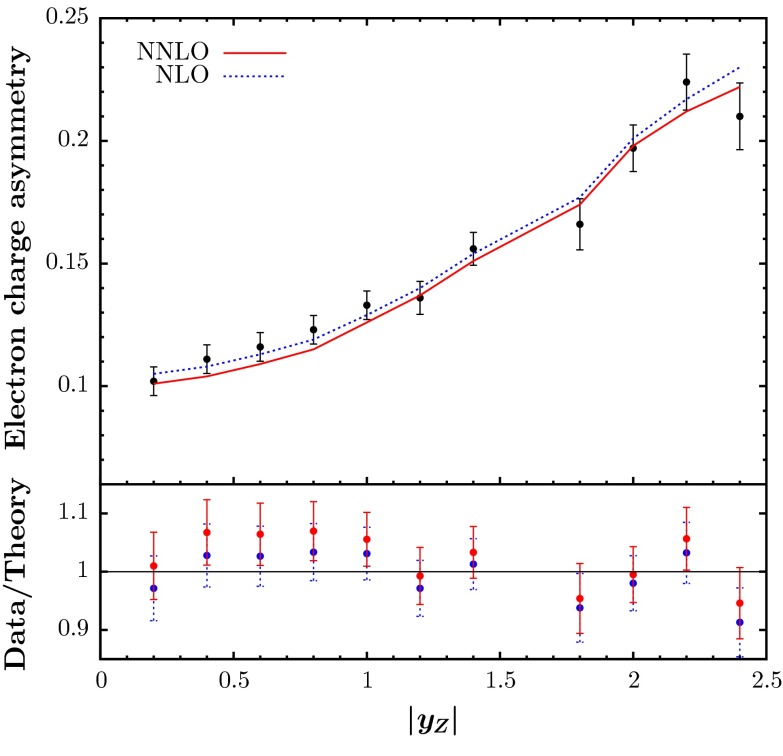
The fit quality for the CMS electron asymmetry data for $p_T>35~\mathrm GeV$ in [9] at NLO and NNLO. Note that correlated uncertainties are made available in the form of a correlation matrix, so the shift of data relative to theory cannot be shown, and makes a comparison of data with PDF uncertainties less useful

Next we discuss the description of the charge lepton asymmetries observed in the CMS data [9, 77]. These data were also not well described by MSTW2008 PDFs, but as seen in Table 2, the prediction using the MMSTWW set at NLO is very good. However, it is still not ideal when using the NNLO set. If we implement the changes discussed above, in the present article, but before including the LHC data, the prediction for these data deteriorates at NLO (due to $u_V(x)-d_V(x)$ becoming too large at $x\sim 0.01$) while it improves slightly at NNLO. When the LHC data are included, we see from Table 2 that the fit quality becomes excellent. This is particularly the case at NLO, where the fit is about as good as possible, but the NNLO description is nearly as good. The fit quality is shown in Fig. 8, and indeed the NLO fit is excellent, but at NNLO there is a slight tendency to undershoot the low rapidity data, but this is exaggerated by the fact that only uncorrelated uncertainties are shown.

#### LHCb $W$ and $Z$ data

**Fig. 9:**
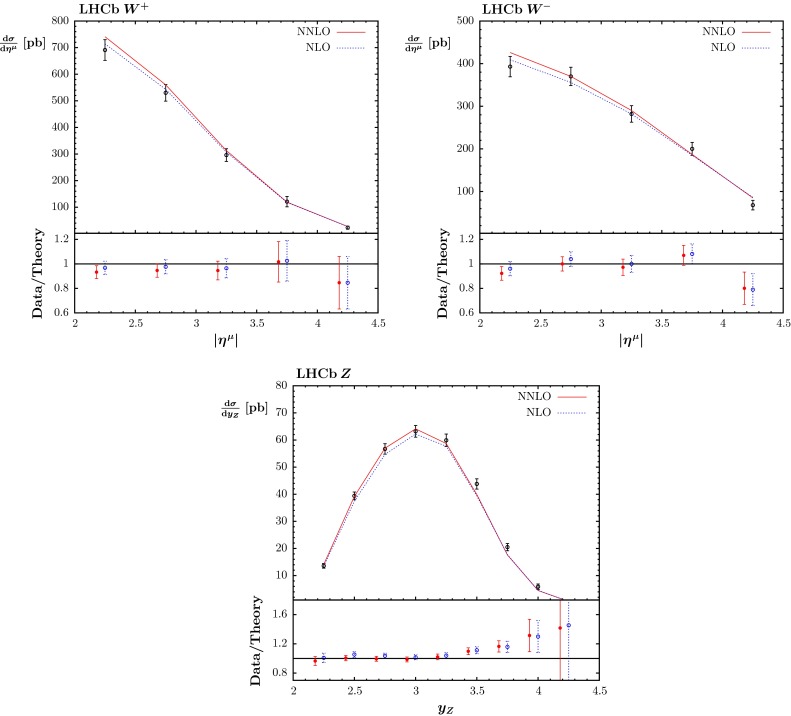
The fit quality for the LHCb data for $W$ and $Z$ production in [78] and [79] at NLO and NNLO. Note that correlated uncertainties are made available in the form of a correlation matrix, so the shift of data relative to theory cannot be shown. The plots show $\mathrm{d}\sigma /\mathrm{d}\eta ^{\mu }$ versus $\eta ^{\mu }$, and $\mathrm{d}\sigma /\mathrm{d}y_Z$ versus $y_Z$

We also include the results for $W^\pm$ production [78] and for $Z \rightarrow e^+e^-$ [79] obtained by the LHCb experiment. These data are both predicted and fitted well at NLO. At NNLO the description is a little worse and is significantly under some of the data points for rapidity $y \approx 3.5$ for the $Z\rightarrow e^+e^-$ data. However, this small discrepancy is not evident when we compare with the preliminary higher precision $Z\rightarrow \mu ^+\mu ^-$ data [80]. The fit quality is shown in Fig. 9. The tendency to undershoot the high rapidity $Z$ data is clear, but this is not an obvious feature of the comparison to the $W^{\pm }$ data. In principle, there are electroweak corrections, including those where the photon distribution appears in the initial state, which are potentially significant. However, the electroweak corrections are still somewhat smaller than the data uncertainty, so we use the pure QCD calculation in this article, though these data, and further measurements, will be an essential feature of a future update of [81] which will appear shortly; see also [82].

#### CMS $Z\rightarrow e^+e^-$ and ATLAS high-mass Drell–Yan data

**Fig. 10:**
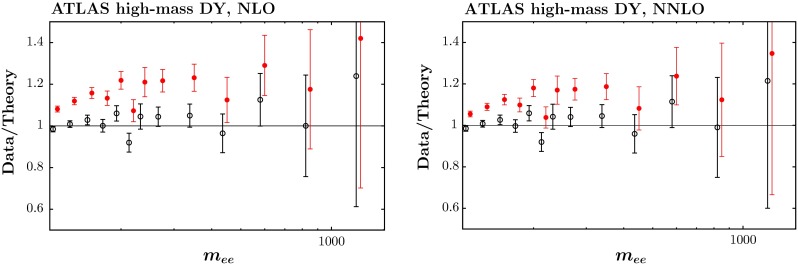
The fit quality for the ATLAS high-mass Drell–Yan data set [83] at NLO (*left*) and NNLO (*right*). The *red points* represent the ratio of measured data to theory predictions, and the b*lack points* (clustering around Data/Theory $=$ 1) correspond to this ratio once the best fit has been obtained by shifting theory predictions relative to data by using the correlated systematics

In addition, we include in the fit the CMS data for $Z\rightarrow e^+e^-$ [84], and the ATLAS high-mass Drell–Yan data [83]. Both are well described, again slightly better at NLO than at NNLO. The fit quality for the ATLAS high-mass Drell–Yan data is shown in Fig. 10. The correlated uncertainties clearly play a big part in allowing the good quality fit, particularly at NLO. However, these are presented in the form of correlation matrices so it is not possible to illustrate shifts of data relative to theory. For these data sets the variation of the theory predictions within the range of PDF uncertainties is smaller than the data uncertainties. As in the previous subsection, in principle there are electroweak corrections, including those where the photon distribution appears in the initial state, which is particularly relevant for this type of process, and they are included in the analysis of [83], which takes the photon PDF from [81], and used as a very weak constraint on the photon PDF in [85]. However, as in the last subsection these are still much smaller than the data uncertainty, though this may well not continue with future measurements.

#### CMS double-differential Drell–Yan data

**Fig. 11:**
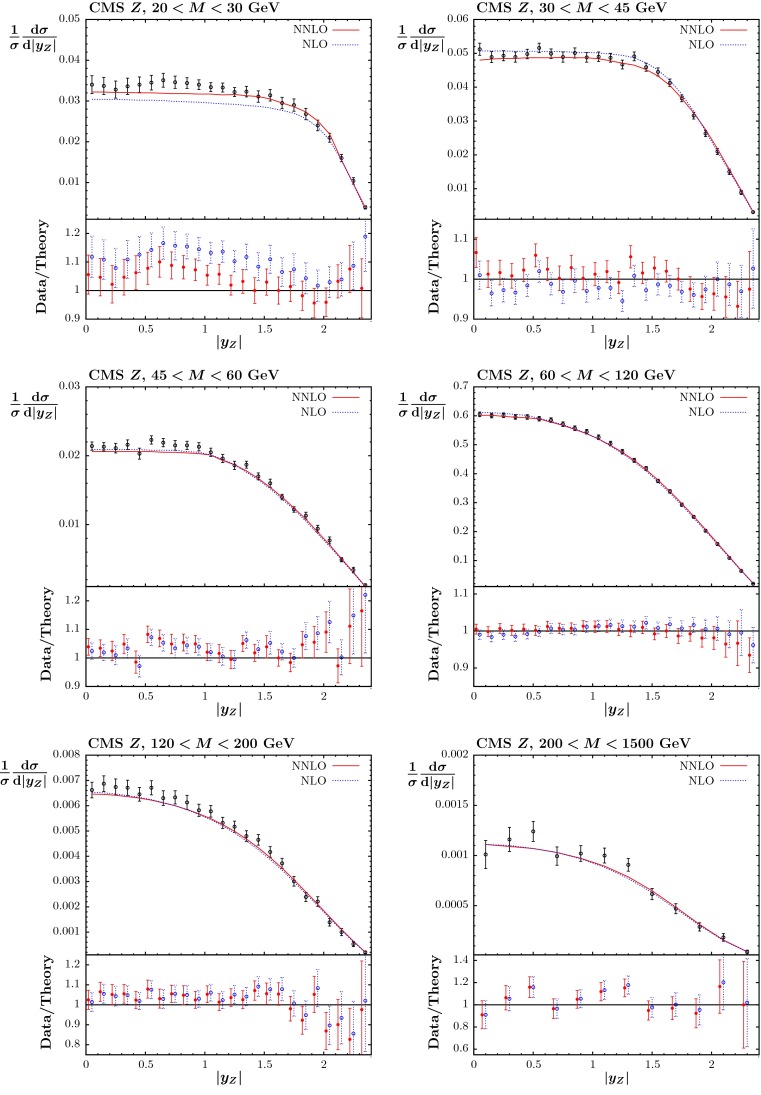
The fit quality for the CMS double-differential Drell–Yan data for $(1/\sigma _Z)\cdot \mathrm{d}\sigma /\mathrm{d}|y_Z|$ versus $|y_Z|$, in [86], for the lowest two mass bins ($20<M<30$ and $30<M<45$ GeV) (*top*), the mass bins ($45<M<60$ and $60<M<120$ GeV) (*middle*) and the mass bins ($120<M<200$ and $200<M<1500$ GeV) (*bottom*), at NLO and NNLO. Note that correlated uncertainties are made available in the form of a correlation matrix, so the shift of data relative to theory cannot be shown

Finally, we include the CMS double-differential Drell–Yan data [86] extending down to relatively low masses, $M(\ell ^+\ell ^-) \sim 20$–$40$ GeV. (Again there is some sensitivity to electroweak corrections away from the $Z$-peak, but we do not include these corrections in the theoretical calculations.) The fit to these data is extremely poor at NLO, as shown in Table 2, and this is largely due to the comparison in the two lowest mass bins $20$–$30$ and $30$–$45~\mathrm GeV$; see Fig. 11. The data/theory comparison in the other mass bins is similar at NLO and NNLO, being very good in both cases. The fit quality can only be improved marginally if this data set is given a very high weighting in the fit – the PDFs are probed at similar values of $x$ in adjacent mass bins, and if the normalisation is changed to improve the match to data in one mass bin it affects the quality in the nearby bins. The fit quality is hugely improved at NNLO, as shown in Fig. 11. This might be taken as an indication that NNLO corrections are particularly important for low-mass Drell–Yan production. However, it is a little more complicated than this. The $p_T$ cut on one lepton in the final state is $14~\mathrm GeV$ (the other is $9~\mathrm GeV$), meaning that at LO the minimum invariant mass is $28~\mathrm GeV$, and most of the lowest mass bin in the double-differential cross section receives a contribution of zero from the LO calculation, and in this region the first non-zero results are at $\mathcal{O}(\alpha _S)$ when an extra particle is emitted. Hence, the $K$-factor going from LO to NLO is over 6 in the $20$–$30~\mathrm GeV$ region, and is still large $\sim$1.3 when going from NLO to NNLO. The $K$-factors are much smaller in higher-mass bins. Hence, it is perhaps more correct to say that the NLO fit is poor because for the lowest mass it is effectively (nearly) a LO calculation, rather than because the NNLO correction is intrinsically very important. A similar effect is noted in the low-mass single-differential measurement in [87], where the prediction using MSTW2008 PDFs at NNLO is very good, but it is poor at NLO at low mass, and fits performed in this paper work well at NNLO, but not at NLO.

#### Procedure for LO fit to Drell–Yan data

At LO we follow the procedure for fitting Drell–Yan (vector boson production) data given in [1]. In this, and other previous studies, it has been found that it is not possible to obtain a good simultaneous fit of structure function and Drell–Yan data, since the quark (and antiquark) distributions are not compatible due to NLO corrections to coefficient functions being much larger for Drell–Yan production. This is because of a significant difference between the result in the space-like and time-like regimes; that is, there is a factor of $1 + (\alpha _S(M^2)/\pi ) C_F\pi ^2/2$ at NLO in the latter regime. Even for $Z$ production this is a factor of $1.25$. Hence, as in [1] we include this common factor for all vector boson production in the LO fit. Doing this enables a good fit to the low-energy fixed-target Drell–Yan data [88] (though it is less good for the asymmetry [89]). However, the general fit quality to rapidity-dependent data from the LHC and the Tevatron is generally poor (with some exceptions, which are generally ratios, e.g. the D0 $Z$-rapidity data [90], and the CMS lepton asymmetry data), with neither the precise normalisation or the shape being correct. Nevertheless, the fit is distinctly better when including the correction factor than without it, while the normalisation is consistently very poor. We do not include the CMS double-differential Drell–Yan data at LO, since, as mentioned above, in the lowest mass bins the LO contribution is an extremely poor approximation.

### Data on $t\bar{t}$ pair production

We include in the fit the combined measurement of the D0 and CDF experiments for the $t\bar{t}$ production cross section as measured at the Tevatron [91]

22 $$\begin{aligned} \sigma (t\bar{t})=7.60\pm 0.41~\mathrm{pb\,\,\,\, with\,\,\,\,}m_t=172.5~\mathrm GeV, \end{aligned}$$

together with published $t\bar{t}$ cross-section measurements from ATLAS and CMS at $\sqrt{s}=7$ TeV [92–102] and at 8 TeV [103].^7^ We use APPLGrid–MCFM at NLO and the code from [104] for the NNLO corrections. We take $m_t=172.5$ GeV (defined in the pole scheme) with an error of 1 GeV, with the corresponding $\chi ^2$ penalty applied. A variation of $1~\mathrm GeV$ in the mass is roughly equivalent to a $3\,\%$ change in the cross section. A number of the measurements of the cross section, including the most precise [99], use the same value of the mass as default. Some also parameterise the measured cross section as a function of $m_t$, and in these cases the cross section falls with increasing mass, as for the theory prediction. However, the dependence is weaker, typically $\sim$1 % per GeV or less, and so this variation is outweighed significantly by the variation in the theory (though one can assume that the 1 GeV uncertainty on the top mass used in the theory calculation is partially accounting for the variation of the cross section data as well, and the uncertainty on the top mass applied is consequently slightly less than 1 GeV in practice).

**Fig. 12:**
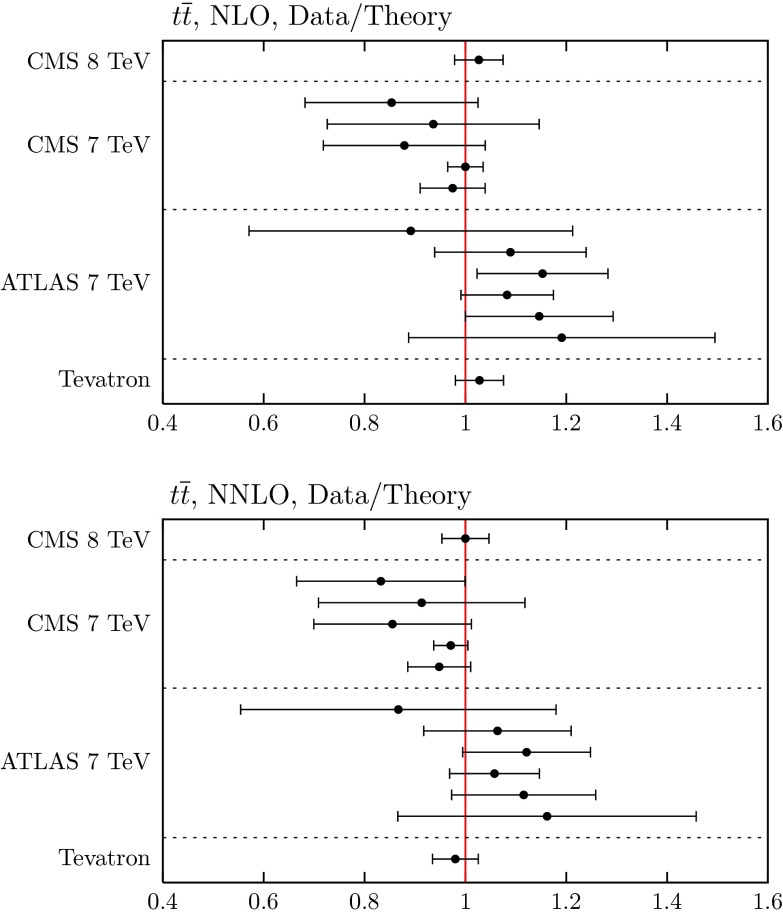
The fit quality of the cross section data for $t\bar{t}$ production ($\sigma ({t \bar{t}})$) at NLO (*top*) and NNLO (*bottom*)

The predictions and the fit are very good, as shown in Table 3, and in Fig. 12, with a slightly lower mass $m_t = 171.7~\mathrm GeV$ preferred in the NLO fit, and a slightly higher value $m_t = 174.2~\mathrm GeV$ in the NNLO fit. Using the dynamical tolerance method both NLO and NNLO fits constrain the top mass to within about $0.7$–$0.8~\mathrm GeV$ of the best-fit values, though the best value and uncertainties cannot be interpreted as independent determinations as a preferred value and uncertainty for $m_t$ is input in the analysis. Nevertheless, it is encouraging that the preferred mass at NNLO is consistent with the world average of $173.34\,\pm \, 0.76~\mathrm GeV$ [105], whereas the NLO preferred value is a little low, highlighting the importance of the NNLO corrections, even though the fit quality is similar at both orders. There is a significant interplay between the gluon distribution, the top mass and the strong coupling constant. It is very clear that as the top quark mass increases the predicted cross section decreases, which can be compensated for in the cross section by an increase in both the gluon and in $\alpha _S(M_Z^2)$. This will be discussed further in a forthcoming article, which presents the variation of PDFs with $\alpha _S(M_Z^2)$ in detail and illustrates the constraint on the coupling. However, we note here that although the fit quality to the $t\bar{t}$ production cross section does depend quite strongly on the values of $m_t$ and $\alpha _S(M_Z^2)$, the small size of the data set is such that the value of $\alpha _S(M_Z^2)$ for the best fit depends very little on variation of $m_t$, or even on the inclusion of the top data, i.e. of order $0.0003$ at most.

**Table 3:** The quality of the description (as measured by the value of $\chi ^2$) of Tevatron and LHC $t \bar{t}$ data before and after they are included in the global NLO and NNLO fits. We also show for comparison the $\chi ^2$ values obtained in the CPdeut fit of the NLO MMSTWW analysis [11], which did not include LHC data. Note that the subprocess $q\bar{q}\rightarrow t\bar{t}$ dominates at the Tevatron with $x_1,x_2 \sim 0.2$, while at the LHC $gg\rightarrow t\bar{t}$ gives the major contribution with $x_1,x_2\sim 0.05$

Data set	$N_\mathrm{pts}$	MMSTWW (Ref. [11])	MMHT2014 (no LHC)	MMHT2014 (with LHC)
NLO				
Tevatron, ATLAS, CMS $\sigma ({t\bar{t}})$	13	8	10	7
NNLO				
Tevatron, ATLAS, CMS $\sigma ({t\bar{t}})$	13	8	11	8

The fit quality at LO is very poor, with $\chi ^2/N_\mathrm{pts}=53/13$. This is because the LO calculation is too low and $m_t=163.5~\mathrm GeV$ is preferred, even though this incurs a very large $\chi ^2$ penalty.

### LHC data on jets

In the present global analysis at NLO we include the CMS inclusive jet data at $\sqrt{s}=7$ TeV with jet radius $R=0.7$ [106], together with the ATLAS data at 7 TeV [107] and at 2.76 TeV with jet radius $R=0.4$ [108]. For the latter we use cuts proposed in the ATLAS study, which eliminate the two lowest $p_T$ points in each bin, due to the large sensitivity to hadronisation corrections in these bins, and some of the highest $p_T$ points.^8^ We perform the calculations within the fitting procedure using FastNLO [110] version 2 [111], which uses NLOJet$++$ [112, 113], and APPLGrid. The jet data from the two experiments appear to be extremely compatible with each other. The data are both well predicted and well fit, as shown in Table 4. Before these data are included in the fit we find $\chi ^2 =107$ for 116 data points for ATLAS and $\chi ^2=143$ for the 133 CMS jet data points at NLO, very similar to the values of $\chi ^2$ obtained from the earlier MMSTWW NLO PDF set. Including these jet data in the NLO fit leads to more improvement in the $\chi ^2$ for CMS than for the ATLAS data, i.e. $143 \rightarrow 138$ as opposed to $107\rightarrow 106$. However, in both cases the possible improvement is rather small. We note that the treatment of the systematic uncertainties for the CMS jet data has been modified to take account of an increased understanding by the experiment since the original publication of the data [106]. Initially the the single pion related correlated uncertainties were all correlated. However, in [114] a decision was made to decorrelate single pion systematics, i.e. to split the single pion source into five separate parts. This lowers the $\chi ^2$ obtained in the best fit significantly, from about 170 to about 135. However, it leads to no real change in PDFs extracted in the global fit, though it allows a slightly higher value of $\alpha _S(M_Z^2)$. The fit quality for the LHC jet data is shown at NLO in Figs. 13, 14 and 15. One can see that the correlated uncertainties play a significant role in enabling the good fit quality, with the shift of data against theory being larger than the uncorrelated uncertainties. However, for each of the three data sets the shape of the data/theory comparison is very good even before the correlated systematics are applied, with only a small correction of order $10\,\%$ at most needed, this being relatively independent of $p_T$, rapidity, or even data set.^9^

**Table 4:** The quality of the description (as measured by the value of $\chi ^2$) of the LHC inclusive jet data before and after they are included in the global NLO and NNLO fits. We also show for comparison the $\chi ^2$ values obtained in the CPdeut fit of the NLO MMSTWW analysis [11], which did not include LHC data. Also the LHC jet data are not included in the final NNLO MMHT global fit presented in this paper. However, the NNLO $\chi ^2$ numbers and $K$ factors mentioned in the table correspond to an exploratory approximate NNLO study described in Sect. 4.3.1

Data set	$N_\mathrm{pts}$	MMSTWW (Ref. [11])	MMHT2014 (no LHC)	MMHT2014 (with LHC)
NLO				
ATLAS jets ($2.76+7$ TeV)	116	107	107	106
CMS jets (7 TeV)	133	140	143	138
NNLO small $K$-factor				
ATLAS jets ($2.76+7$ TeV)	116	(107)	(123)	(122) 115
CMS jets (7 TeV)	133	(142)	(137)	(138) 137
NNLO large $K$-factor				
ATLAS jets ($2.76+7$ TeV)	116	(117)	(132)	(132) 126
CMS jets (7 TeV)	133	(145)	(137)	(139) 139

Of course, the full NNLO QCD calculation is not available for jet cross sections, either in DIS or in hadron–hadron collisions. The NNLO calculation of jet production is ongoing, but not yet complete. It is an enormous project and much progress has been made; see [115–117], and it will hopefully be available soon.

Despite the absence of the full NNLO result, in the NNLO MSTW analysis the Tevatron jet data [118, 119] were included in the fit using an approximation based on the knowledge of the threshold corrections [120]. It was argued that although there was no guarantee that these give a very good approximation to the full NNLO corrections, in this case the NLO corrections themselves are of the same order as the systematic uncertainties on the data. The threshold corrections are the only expected source of possible large NNLO corrections, so the fact that they provide a correction which is smooth in the $p_T$ of the jet and moderately small compared to systematic uncertainties in the data strongly implies that the full NNLO corrections would lead to little change in the PDFs. Since these jet data are the only good direct constraint on the high-$x$ gluon it was decided to include them in the NNLO fit judging that the impact of leaving them out would be far more detrimental than any inaccuracies in including them without knowing the full NNLO hard cross section.

In fact the threshold corrections to the Tevatron data gave about a 10 $\%$ positive correction; see for example Fig. 50 in [109]. We also see from the same figure that the threshold corrections for the LHC data are similar to those at the Tevatron for the highest $x$ values at which jets are measured, but blow up at the low $x$ values probed, that is, when they are far from threshold. Recent detailed studies exploring the dependence of the threshold corrections on the jet radius $R$ values at NLO and NNLO show that the true corrections in the threshold region show a significant dependence^10^ on $R$ at NLO [121, 122], but that this is rather reduced at NNLO [122]. However, the improved NNLO threshold calculations in [122] show that there are still problems at low and moderate values of jet $p_T$.

In the present global analysis, as a default at NNLO, we still include the Tevatron jet data in the fit. This seems reasonable, since they are always relatively near threshold, and the corrections do not obviously break down at the lowest $p_T$ values of the jet.^11^ On the other hand, we omit the LHC jet data, since at the lowest $p_T$ measured the threshold corrections are not stable and, moreover, have large uncertainties at the highest rapidities observed. This is slightly more blunt, but quite similar in practice to the conclusion of [124] which compares the degree of agreement between the approximate threshold calculation and the exact calculation for the $gg \rightarrow gg$ channel, where the latter is known. It is found that the agreement is good for high values of $p_T$ (relative to centre-of-mass energy $\sqrt{s}$) and relatively central rapidity. These regions of agreement are then deemed to be the regions where the approximate NNLO is likely quite reliable. They correspond to most of the Tevatron data, except at high rapidity (where the systematic errors on data are large), much of the CMS jet data, but little of the ATLAS jet data. Hence, we feel confident including the Tevatron jet data using approximate NNLO expressions, especially given that in [109] we investigated the effect of rather dramatic modifications of these corrections, finding only rather moderate changes in PDFs and $\alpha _S(M_Z^2)$. We could arguably include (much of) the CMS jet data, but for the moment err on the side of caution.

#### Exploratory fits to LHC jet data at ‘NNLO’ 

Despite leaving the LHC jet data out of the PDF determination at NNLO we have explored the effect of including very approximate NNLO corrections to the LHC data based on the threshold corrections and the known exact calculations so far available. To do this, we applied a $5$–$20\,\%$ positive correction, growing at the lower $p_T$ values, that is, similar to the shape of the NNLO/NLO corrections in Figures 2 and 3 of [116]. In detail, we have used

23 $$\begin{aligned} K_\mathrm{NNLO/NLO}= & {} (1\!+\! k(9.2 \!-\! 0.5\ln (p_T^2))/9.2),\quad \mathrm{CMS},\quad \end{aligned}$$

24 $$\begin{aligned} K_\mathrm{NNLO/NLO}= & {} (1+ k(8.0 - 0.5\ln (p_T^2))/8.0),\nonumber \\&\quad \mathrm{ATLAS ~7~TeV},\end{aligned}$$

25 $$\begin{aligned} K_\mathrm{NNLO/NLO}= & {} (1+ k(8.0 - 0.5\ln ((7/2.76)^2p_T^2))/8.0),\nonumber \\&\quad \mathrm{ATLAS ~2.76~TeV}. \end{aligned}$$

**Fig. 16:**
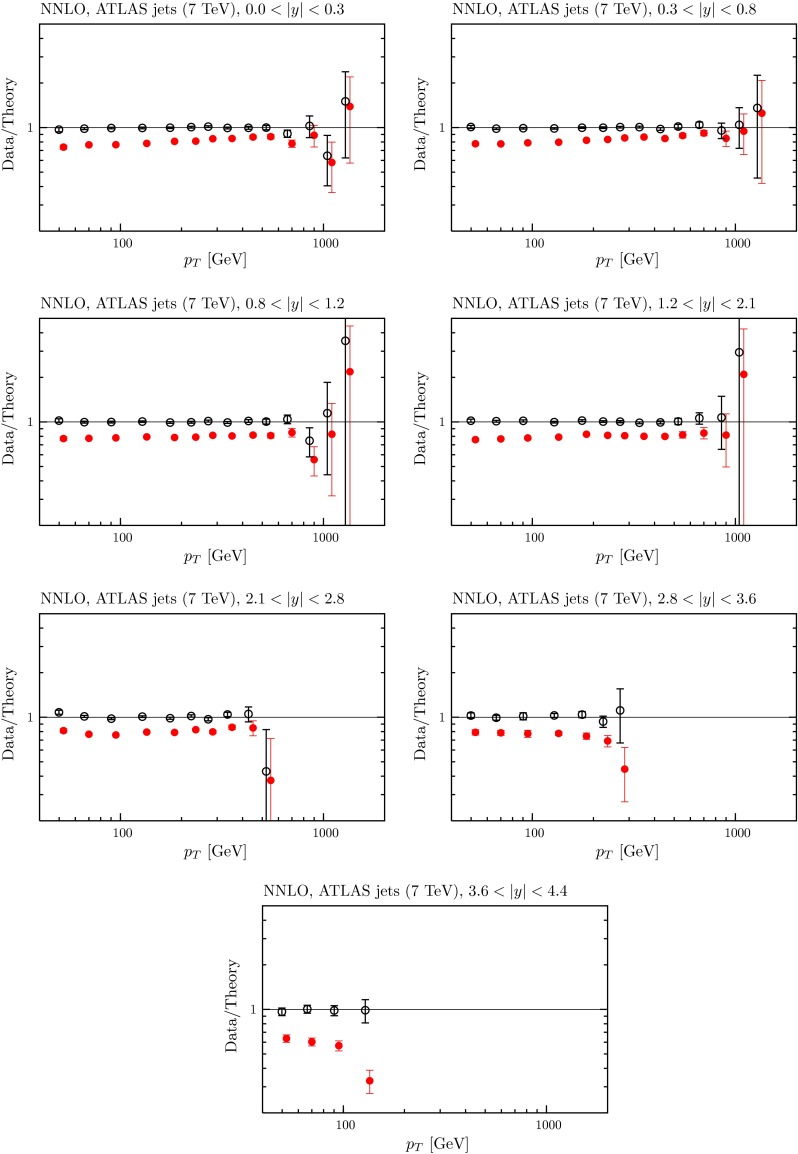
The fit quality for the ATLAS $7~\mathrm TeV$ jet data [107] at NNLO, using the ‘larger’ $K$-factor described in the text. The *red points* represent the ratio of measured data to theory predictions, and the *black points* (clustering around Data/Theory $=$ 1) correspond to this ratio once the best fit has been obtained by shifting theory predictions relative to data by using the correlated systematics

**Fig. 17:**
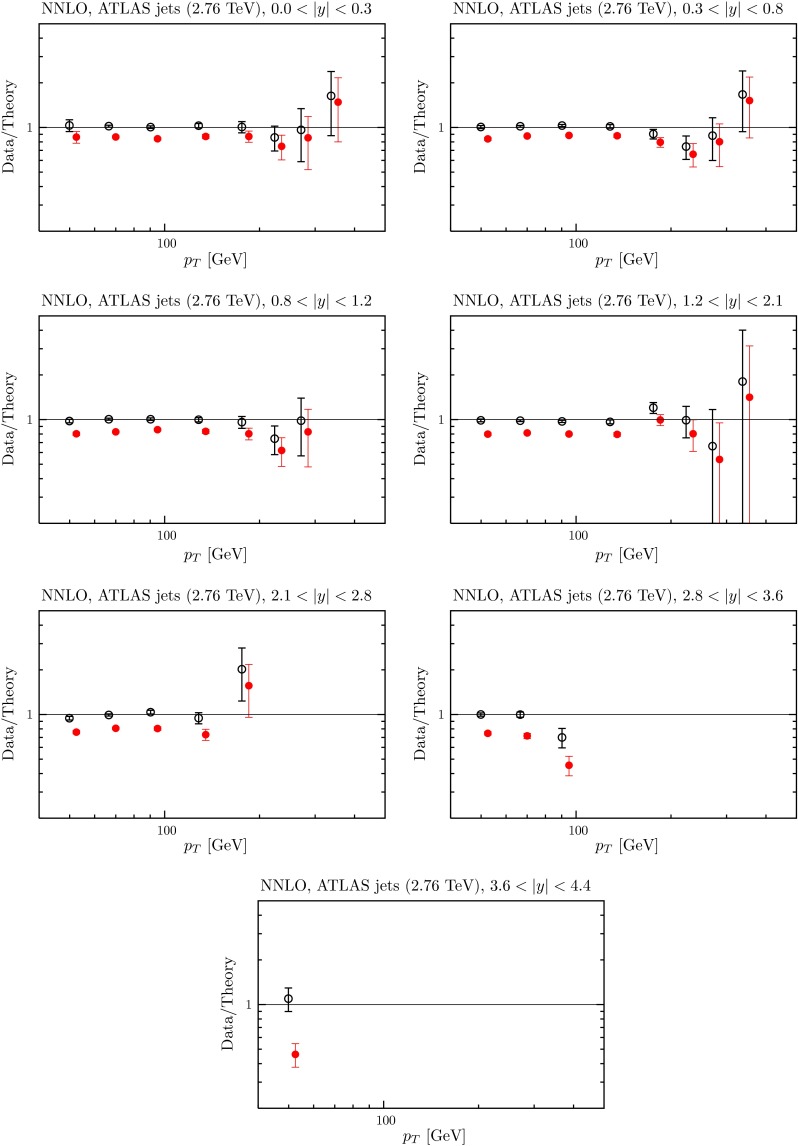
The fit quality for the ATLAS $2.76~\mathrm TeV$ jet data [108] at NNLO, using the ‘larger’ $K$-factor described in the text. The *red points* represent the ratio of measured data to theory predictions, and the *black points* (clustering around Data/Theory $=$ 1) correspond to this ratio once the best fit has been obtained by shifting theory predictions relative to data by using the correlated systematics

**Fig. 18:**
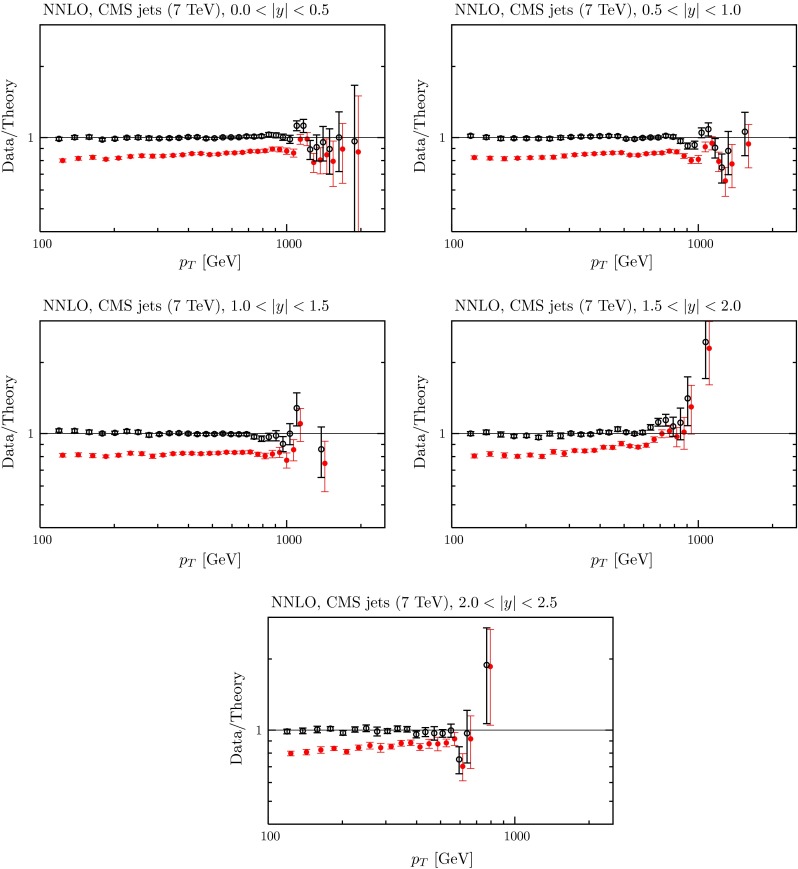
The fit quality for the CMS $7~\mathrm TeV$ jet data [106] at NNLO, using the ‘larger’ $K$-factor described in the text. The *red points* represent the ratio of measured data to theory predictions, and the *black points* (clustering around Data/Theory $=$ 1) correspond to this ratio once the best fit has been obtained by shifting theory predictions relative to data by using the correlated systematics

We tried two alternatives, a ‘smaller’ and ‘larger’ $K$-factor, i.e. $k=0.2$ and $k=0.4$, with corrections of about 10 and 20 $\%$ at $p_T=100$ GeV, independent of rapidity. The quality of the comparison to the data is shown in Table 4 using both the smaller and larger $K$-factors. The numbers in brackets represent predictions rather than a new fit. Clearly for both MMSTWW and MMHT PDFs the quality of the prediction for the CMS data is similar to that for the predictions, and the best fit, at NLO, using either choice of $K$-factor. For the ATLAS data the prediction using MMSTWW PDFs is also similar to the best NLO results with the smaller $K$-factor, but it deteriorates a little with the larger $K$-factor. The predictions using MMHT are slightly worse, and again there is more deterioration with increasing $K$-factor. The greater deterioration for ATLAS data seems to be due to the fact that while the fit to data is not changed much by $K$-factors of $10$–$20\,\%$ at NNLO, the ATLAS data are sensitive to the relative change of the theoretical calculation between the two energies, which is rather difficult to approximate/guess accurately. Even so, in this case the comparison to data is still quite good, even with the larger $K$-factors. The fit quality for the LHC jet data is shown at NNLO, using the larger $K$-factor, in Figs. 16, 17 and 18. One can see that the shape of data relative to theory remains very good, but the discrepancy before correlated uncertainties are applied is now larger in magnitude. This seems to cause little problem for the fit quality for CMS data, but the fact that the relative size of the mismatch between “raw” theory and data is different for the two energies for the ATLAS measurement leads to some limited deterioration in the fit quality.

We have also tried the experiment of including the CMS and ATLAS jet data into the MMHT2014 fit with each of the $K$-factors. The quality is then shown by the unbracketed numbers in the right-hand column of Table 4. The fit quality to the jet data improves slightly, mainly for ATLAS data, though it is still slightly worse than for the NLO fit. The PDFs and $\alpha _S(M_Z^2)$ change extremely little when the LHC jet data are included in the NNLO fit (discussed a little more later), and the fit quality to the other data increases by at worst a couple of units in $\chi ^2$. 12

#### Jet data in the LO fit

In the LO fit, where the cross section is calculated at order $\mathcal{O}(\alpha _S^2)$, the jet data are all included. The fit quality to both LHC and Tevatron data is worse than at NLO, but only with an increase in $\chi ^2$ of $10$–$20\,\%$, except for ATLAS data where we obtain $\chi ^2/N_\mathrm{pts}=162/116$. The fit does normalise the Tevatron data downwards quite significantly, but this is not so apparent for the LHC data, partially due to the much smaller normalisation uncertainties at the LHC.

## Results for the global analysis

The previous section shows the quality of the description of the LHC data before and after they are included in both the NLO and the NNLO global fit. In this section we discuss the overall fit quality and the resulting parton distributions functions. We also compare the results with the MSTW 2008 PDFs.

The parameterisation of the input PDFs is as discussed in Sect. 2.1, and we now treat the coefficients of the first two Chebyshev polynomials for the $s_{+}$ distribution as free, unlike the case before inclusion of LHC data. At LO we make some changes to the parameterisation to stop the PDFs behaving peculiarly in regions where they are not directly constrained – there is a tendency for a large negative contribution in a very limited region of $x$ which would provide a negative contribution to the momentum sum rule, and for $s_{+}$ to become extremely large at very small $x$. Hence, we only allow the first Chebyshev polynomial for $s_{+}$ to be free at LO and parameterise the gluon with four free Chebyshev polynomials, but no second term. This means that both $s_{+}$ and the gluon have one fewer free parameter at LO than at NLO or NNLO.

### The values of the QCD coupling, $\alpha _S(M^2_Z)$

At both NLO and at NNLO the value of $\alpha _S(M_Z^2)$ is allowed to vary as a free parameter in the fit. At NLO the best value of the QCD coupling is found to be

26 $$\begin{aligned} \alpha _{S,\mathrm{NLO}}(M^2_Z)=0.1201. \end{aligned}$$

This is extremely similar to the value of $0.1202$ found in [1]. At NNLO the best value of the QCD coupling is found to be

27 $$\begin{aligned} \alpha _{S,\mathrm{NNLO}}(M^2_Z)=0.1172, \end{aligned}$$

again very similar to that of $0.1171$ in [1] – to be precise only 0.00015 larger. The difference between the NLO and NNLO values has decreased slightly. At LO it is difficult to define an absolute best fit, but the preferred value of $\alpha _S(M_Z^2)$ is certainly in the vicinity of 0.135, so we fix it at this value.

It is a matter of considerable debate whether one should attempt to extract the value of $\alpha _S(M_Z^2)$ from PDF fits or simply use it as in input with the value taken from elsewhere – for example, simply to use the world average value [129]. We believe that useful information on the coupling can be obtained from PDF fits, and as our extracted values of $\alpha _S(M_Z^2)$ at NLO and NNLO are quite close to the world average of $\alpha _S(M_Z^2)=0.1185\pm 0.0006$ we regard these as our best fits. We will discuss the variation with $\alpha _S(M_Z^2)$ and the uncertainty in a PDF fit determination in a future publication. However, we elaborate slightly here.

As well as leaving $\alpha _S(M^2_Z)$ as a completely independent parameter, we also include the world average value (without the inclusion of DIS data to avoid double counting) of $\alpha _S(M_Z^2)=0.1187\pm 0.0007$ as a data point in our fit. This changes the preferred values to

28 $$\begin{aligned} \alpha _{S,\mathrm{NLO}}(M^2_Z)=0.1195 \quad \mathrm{and} \quad \alpha _{S,\mathrm{NNLO}}(M^2_Z)=0.1178.\nonumber \\ \end{aligned}$$

**Fig. 19:**
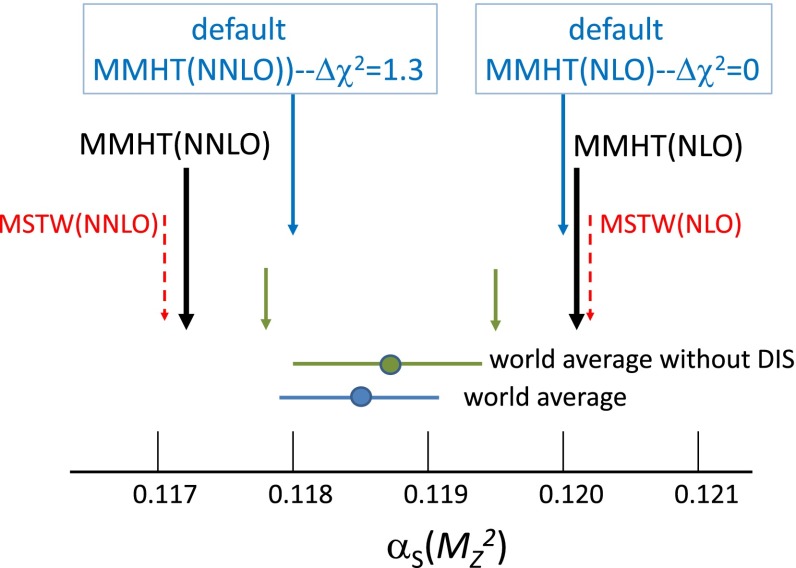
The *dark arrows* indicate the optimal values of $\alpha _S(M_Z^2)$ found in NLO and NNLO fits of the present analysis (MMHT2014). The *dashed arrows* are the values found in the MSTW2008 analysis [1]. These are compared to the world average value, which was obtained assuming, for simplicity, that the NLO and NNLO values are the same – which, in principle, is not the case. The *short arrows* indicate the NLO and NNLO values obtained from the present global analyses if the world average value (obtained without including DIS data) were to be included in the fit. However, the default values $\alpha _{S,\mathrm{NLO}}=0.120$ and $\alpha _{S,\mathrm{NNLO}}=0.118$ are used for the final MMHT2014 PDF sets presented here; the values of $\Delta \chi ^2$ are the changes in $\chi ^2_\mathrm{global}$ in going from the optimal to the default fit

Each of these is about one standard deviation away from the world average, so our PDF fit is entirely consistent with the independent determinations of the coupling. Moreover, the quality of the fit to the data other than the single point on $\alpha _S(M_Z^2)$ increases by about 1.5 units at NLO and just over one unit at NNLO when the coupling value is added as a data point. It is ideal to present PDF sets at common, and hence round values of $\alpha _S(M_Z^2)$ in order to compare with, and combine with, other PDF sets, for example as in [75, 130–132]. At NLO we hence choose $\alpha _S(M_Z^2)=0.120$ as the default value, which is essentially identical to the value for the best PDF fit when the coupling is free, and still very similar when the world average is included as a constraint. At NNLO, when $\alpha _S(M_Z^2)=0.118$ is chosen, the fit quality is still only 1.3 units in $\chi ^2$ higher than that when the coupling is free. This value is extremely close to the value determined when the world average is included as a data point. Hence, we choose to use $\alpha _S(M_Z^2)=0.118$ as the default for our NNLO PDFs, a value which is very consistent with the world average. The summary of this discussion is shown above in Fig. 19. At NLO we also make a set available with $\alpha _S(M_Z^2)=0.118$, but in this case the $\chi ^2$ increases by 17.5 units from the best-fit value.

### The fit quality

The quality of the best fit is shown at LO, NLO and NNLO in Table 5. Note that at NNLO the values are for the absolute best fit with $\alpha _S(M_Z^2)=0.1172$, though the values are generally extremely similar when $\alpha _S(M_Z^2)=0.118$ and the total is $2718.6$ rather than $2717.3$. It has already been noted that both at NLO and NNLO (with the exception of the CMS double-differential data at NLO) the fit quality is excellent. In most cases there is little improvement in the quality of the fit from the inclusion of the LHC data (the ATLAS $W,Z$ and CMS asymmetry data being minor exceptions). It is clear that the inclusion of the LHC data has not spoilt the fit to any of the non-LHC data in any way at all. The fit quality is very similar to that in [11] for the data sets that are common to both fits, with some small differences being attributable to the changes in the procedure applied in this study, as outlined in, for example, Sects. 2.6 and 2.7. The fit quality for non-LHC data is within a handful of chisquared units of the fit when only non-LHC data were included. In fact, in some cases the two extra free parameters in the total strange distribution in the fit including LHC data leads to an improvement in non-LHC data, despite the extra constraint from new data. For example, at NNLO $\chi ^2/N_\mathrm{pts}=637.7/621$ for the HERA combined structure function data in the full fit compared to $\chi ^2/N_\mathrm{pts}=644.2/621$ in the non-LHC fit (at NLO the non-LHC fit gives $666.0/621$ compared to $678.8/621$ in the full fit). At NNLO the main deterioration, about six units, is in NuTeV structure function data, which is in some tension with ATLAS $W,Z$ data. This is not an issue at NLO.

**Table 5:** The values of $\chi ^2 / N_\mathrm{pts}$ for the data sets included in the global fit. For the NuTeV $\nu N\rightarrow \mu \mu X$ data, the number of degrees of freedom is quoted instead of $N_\mathrm{pts}$ since smearing effects mean nearby points are highly correlated. The details of corrections to data, kinematic cuts applied and definitions of $\chi ^2$ are contained in the text

Data set	LO	NLO	NNLO
BCDMS $\mu p$ $F_2$ [125]	162/153	176/163	173/163
BCDMS $\mu d$ $F_2$ [19]	140/142	143/151	143/151
NMC $\mu p$ $F_2$ [20]	141/115	132/123	123/123
NMC $\mu d$ $F_2$ [20]	134/115	115/123	108/123
NMC $\mu n/\mu p$ [21]	122/137	131/148	127/148
E665 $\mu p$ $F_2$ [22]	59/53	60/53	65/53
E665 $\mu d$ $F_2$ [22]	52/53	52/53	60/53
SLAC $ep$ $F_2$ [23, 24]	21/18	31/37	31/37
SLAC $ed$ $F_2$ [23, 24]	13/18	30/38	26/38
NMC/BCDMS/SLAC/HERA $F_L$ [20, 24, 63–65, 125]	113/53	68/57	63/57
E866/NuSea $pp$ DY [88]	229/184	221/184	227/184
E866/NuSea $pd/pp$ DY [89]	29/15	11/15	11/15
NuTeV $\nu N$ $F_2$ [29]	35/49	39/53	38/53
CHORUS $\nu N$ $F_2$ [30]	25/37	26/42	28/42
NuTeV $\nu N$ $xF_3$ [29]	49/42	37/42	31/42
CHORUS $\nu N$ $xF_3$ [30]	35/28	22/28	19/28
CCFR $\nu N\rightarrow \mu \mu X$ [31]	65/86	71/86	76/86
NuTeV $\nu N\rightarrow \mu \mu X$ [31]	53/40	38/40	43/40
HERA $e^+p$ NC 820 GeV [61]	125/78	93/78	89/78
HERA $e^+p$ NC 920 GeV [61]	479/330	402/330	373/330
HERA $e^-p$ NC 920 GeV [61]	158/145	129/145	125 /145
HERA $e^+p$ CC [61]	41/34	34/34	32/34
HERA $e^-p$ CC [61]	29/34	23/34	21/34
HERA $ep$ $F_2^\mathrm{charm}$ [62]	105 /52	72/52	82/52
H1 99–00 $e^+p$ incl. jets [126]	77/24	14/24	–
ZEUS incl. jets [127, 128]	140/60	45/60	–
DØ II $p\bar{p}$ incl. jets [119]	125/110	116/110	119/110
CDF II $p\bar{p}$ incl. jets [118]	78/76	63/76	59/76
CDF II $W$ asym. [66]	55/13	32/13	30/13
DØ II $W\rightarrow \nu e$ asym. [67]	47/12	28/12	27/12
DØ II $W\rightarrow \nu \mu$ asym. [68]	16/10	19/10	21/10
DØ II $Z$ rap. [90]	34/28	16/28	16/28
CDF II $Z$ rap. [70]	95/28	36/28	40/28
ATLAS $W^+, W^-, Z$ [10]	94/30	38/30	39/30
CMS $W$ asymm $p_T >35~\mathrm GeV$ [9]	10/11	7/11	9/11
CMS asymm $p_T >25~\mathrm GeV,30~\mathrm GeV$ [77]	7/24	8/24	10/24
LHCb $Z\rightarrow e^+e^-$ [79]	76/9	13/9	20/9
LHCb $W$ asymm $p_T >20~\mathrm GeV$ [78]	27/10	12/10	16/10
CMS $Z\rightarrow e^+e^-$ [84]	46/35	19/35	22/35
ATLAS high-mass Drell–Yan [83]	42/13	21/13	17/13
CMS double-diff. Drell–Yan [86]	–	372/132	149/132
Tevatron, ATLAS, CMS $\sigma _{t\bar{t}}$ [91–97]	53/13	7/13	8/13
ATLAS jets (2.76 +7 TeV) [107, 108]	162/116	106/116	–
CMS jets (7 TeV) [106]	150/133	138/133	–
All data sets	**3706/2763**	**3267/2996**	**2717/2663**

Overall the quality of the NNLO fit is 247 units in $\chi ^2$ lower when counted for the data which are included in both fits, though this is reduced to only 25 units when the CMS double-differential Drell–Yan data are removed from the comparison. Some of the data sets within the global fit have a lower $\chi ^2$ at NLO than at NNLO. It would be surprising if the total $\chi ^2$ were lower at NLO, but this is not impossible: even though one would expect NNLO to be closer to the “ideal” theory prediction fluctuations in data could allow an apparently better fit quality to a worse prediction. On the other hand, given that NLO and NNLO are in general not very different predictions for most quantities it is quite possible that the shape of the PDFs obtained by the best fit at NNLO results in a best fit where the improvement in fit quality to some data sets is partially compensated by a slight deterioration in the fit to some other data sets. As already noted with the LHC data, the LO fit is sometimes very poor, in particular for the HERA jet data where NLO corrections are large.

### Central PDF sets and uncertainties

The parameters for the central PDF sets at LO, NLO and NNLO are shown in Table 6. In order to describe the uncertainties on the PDFs we apply the same procedure as in [1] (originally presented in [133]), i.e. we use the Hessian approach with a dynamical tolerance, and hence obtain a set of PDF eigenvector sets each corresponding to $68\,\%$ confidence level uncertainty and being orthogonal to each other.

**Table 6:** The optimal values of the input PDF parameters (as defined in Sect. 2.1) at $Q_0^2 = 1$ GeV$^2$ determined from the global analyses. $A_u$, $A_d$, $A_g$ and $x_0$ are determined from sum rules and are not fitted parameters. Similarly, $A_\Delta$ is determined from $\int _0^1\mathrm {d}{x}\;\Delta (x,Q_0^2)$

Parameter	LO	NLO	NNLO
$\alpha _S(M_Z^2)$	$0.135$	$0.120$	$0.118$
$A_u$	$1.3358$	$4.2723$	$3.8539$
$\delta _u$	$0.34430$	$0.74687$	$0.70900$
$\eta _u$	$2.2318$	$2.7421$	$2.8773$
$a_{u,1}$	$-0.26767$	$0.26349$	$0.80527$
$a_{u,2}$	$-0.51620$	$-0.00256$	$-0.19419$
$a_{u,3}$	$0.47167$	$0.25858$	$0.27225$
$a_{u,4}$	$-0.12224$	$0.05000$	$-0.01211$
$A_d$	$3.6009$	$3.3002$	$7.5602$
$\delta _d$	$0.25049$	$0.90012$	$1.1147$
$\eta _d-\eta _u$	$2.3847$	$-0.58802$	$-0.25180$
$a_{d,1}$	$-1.3817$	$1.2898$	$1.2663$
$a_{d,2}$	$0.49690$	$0.60385$	$0.78475$
$a_{d,3}$	$-0.040740$	$0.33590$	$0.32372$
$a_{d,4}$	$-0.03926$	$0.26150$	$0.25099$
$A_S$	$18.597$	$31.329$	$43.726$
$\delta _S$	$-0.09018$	$-0.13358$	$-0.03946$
$\eta _S$	$10.922$	$11.945$	$12.776$
$a_{S,1}$	$-1.5611$	$-1.6020$	$-1.5979$
$a_{S,2}$	$0.85903$	$0.86538$	$0.87445$
$a_{S,3}$	$-0.30427$	$-0.29923$	$-0.30196$
$a_{S,4}$	$0.07061$	$0.06022$	$0.006227$
$\int _0^1\mathrm {d}{x}\;\Delta (x,Q_0^2)$	$0.15782$	$0.09531$	$0.081983$
$A_\Delta$	$0.29972$	$7.1043$	$25.408$
$\delta _\Delta$	$0.60594$	$1.7116$	$2.1602$
$\gamma _\Delta$	$13.029$	$10.659$	$8.1584$
$\epsilon _\Delta$	$46.611$	$-33.341$	$-36.418$
$A_g$	$17.217$	$0.88746$	$0.53411$
$\delta _g$	$-0.33293$	$-0.45853$	$-0.56889$
$\eta _g$	$5.3687$	$2.8636$	$1.3022$
$a_{g,1}$	$-1.664$	$-0.36317$	$0.56995$
$a_{g,2}$	$0.99169$	$0.20961$	$0.37592$
$a_{g,3}$	$-0.42245$	–	–
$a_{g,4}$	$0.10176$	–	–
$A_{g^\prime }$	–	$-1.0187$	$-0.09827$
$\delta _{g^\prime }$	–	$-0.42510$	$-0.57405$
$\eta _{g^\prime }$	–	$32.614$	$22.417$
$A_+$	$2.2447$	$4.6779$	$8.2868$
$\eta _+$	$14.055$	$11.588$	$13.752$
$a_{+,1}$	$-1.5090$	$-1.5910$	$-1.5958$
$a_{+,2}$	–	$0.86501$	$0.88792$
$A_-$	$-0.53737$	$-0.01614$	$-0.011373$
$\eta _-$	$14.402$	$7.1599$	$6.4376$
$\delta _-$	$0.91595$	$-0.26403$	$-0.26403$
$x_0$	$0.056131$	$0.026495$	$0.028993$

#### Procedure to determine PDF uncertainties

In more detail, if we have input parameters $\{a_i^0\}=\{a_1^0,\ldots ,a_n^0\}$, then we write

29 $$\begin{aligned} \Delta \chi ^2_\mathrm{global} \equiv \chi ^2_\mathrm{global} - \chi _\mathrm{min}^2 = \sum _{i,j=1}^n H_{ij}(a_i-a_i^0)(a_j-a_j^0), \end{aligned}$$

where the Hessian matrix $H$ has components

30 $$\begin{aligned} H_{ij} = \left. \frac{1}{2}\frac{\partial ^2\,\chi ^2_\mathrm{global}}{\partial a_i\partial a_j}\right| _\mathrm{min}. \end{aligned}$$

The uncertainty on a quantity $F(\{a_i\})$ is then obtained from standard linear error propagation:

31 $$\begin{aligned} \Delta F = T \sqrt{\sum _{i,j=1}^n\frac{\partial F}{\partial a_i}C_{ij}\frac{\partial F}{\partial a_j}}, \end{aligned}$$

where $C\equiv H^{-1}$ is the covariance matrix, and $T = \sqrt{\Delta \chi ^2_\mathrm{global}}$ is the “tolerance” for the required confidence interval, usually defined to be $T=1$ for $68\,\%$ confidence level.

It is very useful to diagonalise the covariance (or Hessian) matrix [133], and work in terms of the eigenvectors. The covariance matrix has a set of normalised *orthonormal* eigenvectors $v_k$ defined by

32 $$\begin{aligned} \sum _{j=1}^n C_{ij} v_{jk} = \lambda _k v_{ik}, \end{aligned}$$

where $\lambda _k$ is the $k$th eigenvalue and $v_{ik}$ is the $i$th component of the $k$th orthonormal eigenvector ($k = 1,\ldots ,n$). The parameter displacements from the global minimum can be expanded in terms of rescaled eigenvectors $e_{ik}\equiv \sqrt{\lambda _k}v_{ik}$:

33 $$\begin{aligned} \Delta a_i\equiv a_i - a_i^0 = \sum _k e_{ik} z_k, \end{aligned}$$

i.e. the $z_k$ are the coefficients when we express a change in parameters away from their best-fit values in terms of the rescaled eigenvectors, and a change in parameters corresponding to $\Delta \chi ^2_\mathrm{global}=1$ corresponds to $z_k=1$. This results in the simplification

34 $$\begin{aligned} \chi ^2_\mathrm{global} = \chi ^2_\mathrm{min} + \sum _k z_k^2. \end{aligned}$$

Eigenvector PDF sets $S_k^\pm$ can then be produced with parameters given by

35 $$\begin{aligned} a_i(S_k^\pm ) = a_i^0 \pm t\,e_{ik}, \end{aligned}$$

with $t$ adjusted to give the desired $T = \sqrt{\Delta \chi ^2_\mathrm{global}}$. In the limit that Eq. (29) is exact, i.e. there are no significant corrections to quadratic behaviour, $t\equiv T$. We limit our number of eigenvectors so that this is true to a reasonable approximation. This results in the PDF eigenvector sets being obtained by fixing some of the parameters at their best-fit values, otherwise the large degree of correlation between some parameters would lead to significant violations in $t\approx T$.

As in [1] we do not determine the size of the eigenvectors using the standard $\Delta \chi ^2=1$ or $T=1$ rule. Rather, we allow $T \ne 1$ to account, primarily, for the tensions in fitting the *different* data sets within fixed-order perturbative QCD. Neither do we use a fixed value of $T$. Instead we use the “dynamical tolerance” procedure devised in [1]. In brief, we define the 68 % confidence-level region for each data set $n$ (comprising $N$ data points) by the condition that

36 $$\begin{aligned} \chi _n^2 < \left( \frac{\chi _{n,0}^2}{\xi _{50}}\right) \xi _{68}, \end{aligned}$$

where $\xi _{68}$ is the 68th percentile of the $\chi ^2$-distribution with $N$ degrees of freedom, and $\xi _{50}\simeq N$ is the most probable value. For each eigenvector (in each of the two directions) we then determine the values of $t$ and $T$ for which the $\chi _n^2$ for each data set $n$ are minimised, together with $68\,\%$ confidence level limits defined by values at which Eq. (36) ceases to be satisfied. For a perfect data set we would only need the value of $\xi _{68}$, but for a number of data sets $\chi _{n,0}^2$ is not very close to $\xi _{50}$ ($\xi _{50}\sim n_\mathrm{pts}$), being potentially both higher and lower, as seen in Table 5. For more details of the “dynamical tolerance” procedure see Section 6.2 of [1].

#### Uncertainties of the MMHT2014 PDFs

The increase in the parameterisation flexibility in the present MMHT analysis leads to an increase in the number of parameters left free in the determination of the PDF uncertainties, as compared to the MSTW2008 analysis. Indeed, we now have 25 eigenvector pairs, rather than the 20 in [1] or even the 23 in [11]. The 25 parameters^13^ left free for the determination of the eigenvectors consist of: $\eta , \delta , a_2$ and $a_3$ for each of the valence quarks, $A, \eta , \delta , a_2$ and $a_3$ for the light sea; $\int _0^1\mathrm {d}{x}\;\Delta (x,Q_0^2), \eta$ and $\gamma$ for $\bar{d} - \bar{u}$; $\eta , \delta , \eta _-$ and $\delta _-$ for the gluon (or $\eta , \delta , a_2$ and $a_3$ at LO); $A,\eta$ and $a_2$ for $s_+$ (or $A,\eta$ and $a_1$ at LO); and $A$ and $\eta$ for $s_-$. During the determination of the eigenvectors all deuteron parameters, free coefficients for nuclear corrections and all parameters associated with correlated uncertainties, including normalisations, are allowed to vary (some with appropriate $\chi ^2$ penalty).

The most constraining data set for each eigenvector direction, and also the values of $t$ and $T$ are shown for the NLO fit in Table 7. The fractional contribution to the total uncertainty of each PDF is then also shown in summary in Table 8. The same information is shown for the NNLO fit in Tables 9 and 10. One can see that for the vast majority of cases there is good agreement between $t$ and $T$ at both NLO and NNLO. Hence, within the region of $68\,\%$ uncertainty confidence levels for the PDFs, the $\chi ^2$ distribution is quite accurately a quadratic function of the parameters. There is, however, a reasonable degree of asymmetry between the $t$ and $T$ values in the two directions for a single eigenvector, and it is nearly always the case that it is a different data set which is the main constraint in the two directions. In fact, the data set which has the most rapid deterioration in fit quality in one direction is often improving in fit quality until quite a high value of $t$ along the other direction. This is an indication of the tension between data sets, with nearly all eigenvectors having some data sets which pull in opposite directions. The values of $t$ and $T$ for the $68\,\%$ confidence levels are on average about $t\approx T \approx 3$, i.e. $\Delta \chi ^2_\mathrm{global} \approx 10$, though $T^2$ does vary between about 1 unit and at most $T^2\approx 40$.

**Table 7:** Table of expected $\sqrt{\Delta \chi ^2}=t$ and true $\sqrt{\Delta \chi ^2}=T$ values for $68\,\%$ confidence-level uncertainty for each eigenvector and the most constraining data sets for the MMHT2014 NLO fits

Eigen-vector	$+$ $t$	$T$	Most constraining data set	$-t$	$T$	Most constraining data set
1	4.00	3.97	HERA $e^+p$ NC 920 GeV	4.30	4.66	HERA $e^+p$ NC 820 GeV
2	2.50	2.84	HERA $e^+p$ NC 920 GeV	1.80	1.53	NMC $\mu d$ $F_2$
3	3.80	4.00	NMC.....HERA $F_L$	3.70	3.69	NMC $\mu d$ $F_2$
4	4.05	4.00	DØ II $W\rightarrow \nu e$ asym.	5.00	5.11	DØ II $W\rightarrow \nu \mu$ asym.
5	3.40	3.35	DØ II $W\rightarrow \nu \mu$ asym.	4.20	4.45	NuTeV $\nu N\rightarrow \mu \mu X$
6	1.85	1.88	NuTeV $\nu N\rightarrow \mu \mu X$	3.70	3.71	DØ II $W\rightarrow \nu \mu$ asym.
7	1.55	1.67	E866/NuSea $pd/pp$ DY	2.15	2.03	E866/NuSea $pd/pp$ DY
8	2.75	2.64	DØ II $W\rightarrow \nu \mu$ asym.	1.90	2.01	E866/NuSea $pd/pp$ DY
9	3.40	3.46	E866/NuSea $pd/pp$ DY	3.80	3.78	BCDMS $\mu p$ $F_2$
10	3.15	3.47	NuTeV $\nu N\rightarrow \mu \mu X$	2.40	2.13	NuTeV $\nu N$ $F_2$
11	3.80	3.86	CDF II $W$ asym.	4.00	3.96	E866/NuSea $pd/pp$ DY
12	3.70	3.53	SLAC $ed$ $F_2$	3.60	3.81	BCDMS $\mu p$ $F_2$
13	4.30	5.47	HERA $e^+p$ NC 820 GeV	5.30	4.33	NMC $\mu d$ $F_2$
14	3.30	3.36	DØ II $W\rightarrow \nu e$ asym.	2.80	3.42	CMS $W$ asym. $p_T >35~\mathrm GeV$
15	2.90	3.08	NuTeV $\nu N$ $xF_3$	3.30	3.12	E866/NuSea $pp$ DY
16	3.65	3.70	CDF II $p\bar{p}$ incl. jets	2.65	2.64	NuTeV $\nu N$ $xF_3$
17	1.80	1.85	E866/NuSea $pd/pp$ DY	2.40	2.16	E866/NuSea $pd/pp$ DY
18	1.15	1.42	CMS asym. $p_T >25,30~\mathrm GeV$	2.60	3.19	BCDMS $\mu p$ $F_2$
19	2.60	2.86	CMS asym. $p_T >25,30~\mathrm GeV$	2.10	3.35	DØ II $p\bar{p}$ incl. jets
20	1.60	1.72	CCFR $\nu N\rightarrow \mu \mu X$	1.55	1.45	NuTeV $\nu N\rightarrow \mu \mu X$
21	2.80	3.45	NuTeV $\nu N\rightarrow \mu \mu X$	3.30	3.47	ATLAS $W^+, W^-, Z$
22	4.70	6.48	NuTeV $\nu N$ $xF_2$	4.00	3.67	NuTeV $\nu N$ $xF_3$
23	1.90	1.96	NuTeV $\nu N\rightarrow \mu \mu X$	4.85	3.50	CCFR $\nu N\rightarrow \mu \mu X$
24	2.35	3.13	HERA $e^+p$ NC 920 GeV	3.75	4.27	HERA $e^+p$ NC 920 GeV
25	2.50	2.63	E866/NuSea $pd/pp$ DY	1.30	2.15	E866/NuSea $pd/pp$ DY

**Table 8:** The three numbers in each entry are the fractional contribution to the total uncertainty for the $g,u_v,\ldots$ input distributions in the small $x$ ($x<0.01$), medium $x$ ($0.01<x<0.1$) and large $x$ ($x>0.1$) regions, respectively, arising from eigenvector $k$ in the NLO global fit. Each number has been multiplied by ten; for example, 4 denotes 0.4. For a precise value of $x$ the sum of each column should be 10. However, the entries shown are the maximum fraction in each interval of $x$, so often do not satisfy this condition. In general we do not show contributions below $5\,\%$, but for the first two eigenvectors at NLO no uncertainty contribution is this large, so we show the largest contributions

Eigen vector	$g$	$u_v$	$d_v$	$S(\mathrm{ea})$	$\bar{d}-\bar{u}$	$s+\bar{s}$	$s-\bar{s}$
1	–	–	–	0 0.3 0	–	–	–
2	–	–	–	0 0.4 0	–	–	–
3	4 0 0	–	–	–	–	–	–
4	2 0 0	0 0 2	–	–	–	–	–
5	1 0 0	–	–	1 0 0	–	–	1 0 0
6	–	–	–	–	–	–	2 1 2
7	–	–	–	–	0 2 2	–	–
8	–	–	0 0 2	–	0 1 2	–	–
9	–	1 2 3	–	–	0 1 2	–	–
10	–	–	–	2 1 0	–	2 3 1	–
11	–	0 1 2	2 3 4	–	0 1 1	–	–
12	–	4 3 5	1 2 2	0 1 0	–	–	–
13	8 5 2	1 1 1	0 0 1	1 1 0	–	–	–
14	–	–	2 3 7	–	–	–	–
15	1 2 2	1 1 2	2 1 2	0 0 1	1 1 0	–	–
16	0 1 5	1 2 2	0 1 2	0 3 3	1 2 0	–	–
17	–	–	–	0 0 1	2 3 4	–	–
18	–	4 4 0	0 1 0	–	–	–	–
19	–	–	2 3 2	–	–	–	–
20	–	–	–	0 0 1	1 0 0	0 0 6	1 0 0
21	0 0 1	1 2 0	2 1 2	4 4 4	0 1 0	5 6 6	4 3 3
22	1 2 0	1 0 1	2 2 2	4 2 4	0 0 1	2 1 2	1 0 0
23	–	0 1 0	0 0 1	1 0 3	1 0 0	1 2 2	2 8 10
24	0 5 6	–	0 1 1	0 1 0	0 0 1	–	–
25	–	–	–	–	7 4 9	–	–

**Table 9:** Table of expected $\sqrt{\Delta \chi ^2}=t$ and true $\sqrt{\Delta \chi ^2}=T$ values for $68\,\%$ confidence-level uncertainty for each eigenvector and the most constraining data sets for the MMHT2014 NNLO fits

Eigen-vector	$+$ $t$	$T$	Most constraining data set	$-t$	$T$	Most constraining data set
1	3.50	3.41	HERA $e^+p$ NC 920 GeV	4.50	4.78	HERA $e^+p$ NC 820 GeV
2	3.95	3.92	NMC.....HERA $F_L$	3.95	4.03	HERA $e^+p$ NC 920 GeV
3	3.85	4.10	HERA $e^+p$ NC 920 GeV	1.55	1.37	NMC $\mu d$ $F_2$
4	5.00	5.07	BCDMS $\mu p$ $F_2$	5.00	4.99	SLAC $ed$ $F_2$
5	2.50	2.48	DØ II $W\rightarrow \nu \mu$ asym.	2.40	2.46	NuTeV $\nu N\rightarrow \mu \mu X$
6	5.30	5.47	CCFR $\nu N\rightarrow \mu \mu X$	2.30	2.31	NuTeV $\nu N\rightarrow \mu \mu X$
7	1.40	1.46	E866/NuSea $pd/pp$ DY	1.70	1.64	E866/NuSea $pd/pp$ DY
8	2.50	2.60	DØ II $W\rightarrow \nu \mu$ asym.	2.70	2.61	DØ II $W\rightarrow \nu e$ asym.
9	5.70	6.00	HERA *ep* $F_2^\mathrm{charm}$	3.20	3.04	CCFR $\nu N\rightarrow \mu \mu X$
10	3.40	3.13	E866/NuSea $pd/pp$ DY	4.60	4.67	CDF II $W$ asym.
11	4.30	4.41	E866/NuSea $pd/pp$ DY	3.00	2.92	NuTeV $\nu N\rightarrow \mu \mu X$
12	4.85	5.25	HERA *ep* $F_2^\mathrm{charm}$	4.70	4.44	BCDMS $\mu p$ $F_2$
13	1.85	2.14	CMS asym. $p_T >25,30~\mathrm GeV$	4.70	4.34	NuTeV $\nu N$ $xF_3$
14	2.85	3.01	BCDMS $\mu d$ $F_2$	2.55	2.79	CMS $W$ asym. $p_T >35~\mathrm GeV$
15	1.20	0.95	Tevatron, ATLAS, CMS $\sigma _{t\bar{t}}$	3.30	3.72	CDF II $p\bar{p}$ incl. jets
16	1.75	2.01	CMS asym. $p_T >25,30~\mathrm GeV$	3.55	3.43	BCDMS $\mu p$ $F_2$
17	1.75	1.90	CMS asym. $p_T >25,30~\mathrm GeV$	3.30	3.12	E866/NuSea $pd/pp$ DY
18	3.10	3.11	BCDMS $\mu p$ $F_2$	1.40	1.87	CMS asym. $p_T >25,30~\mathrm GeV$
19	1.80	1.84	CMS asym. $p_T >25,30~\mathrm GeV$	2.55	3.26	DØ II $p\bar{p}$ incl. jets
20	2.00	2.20	CCFR $\nu N\rightarrow \mu \mu X$	1.50	1.51	NuTeV $\nu N\rightarrow \mu \mu X$
21	3.00	3.03	ATLAS $W^+, W^-, Z$	4.70	5.49	HERA $e^+p$ NC 920 GeV
22	1.20	1.60	E866/NuSea $pd/pp$ DY	6.90	5.31	NMC $\mu n/\mu p$
23	2.20	2.86	HERA $e^+p$ NC 920 GeV	1.85	3.73	HERA $e^+p$ NC 920 GeV
24	4.30	3.38	CCFR $\nu N\rightarrow \mu \mu X$	1.75	1.86	NuTeV $\nu N\rightarrow \mu \mu X$
25	1.90	3.39	HERA $e^+p$ NC 920 GeV	1.60	2.78	HERA $e^+p$ NC 920 GeV

**Table 10:** The three numbers in each entry are the fractional contribution to the total uncertainty for the $g,u_v,\ldots$ input distributions in the small $x$ ($x<0.01$), medium $x$ ($0.01<x<0.1$) and large $x$ ($x>0.1$) regions, respectively, arising from eigenvector $k$ in the NNLO global fit

Eigen vector	$g$	$u_v$	$d_v$	$S(\mathrm{ea})$	$\bar{d}-\bar{u}$	$s+\bar{s}$	$s-\bar{s}$
1	1 0 0	–	–	–	1 0 0	–	–
2	4 0 0	–	–	–	–	–	–
3	–	–	–	0 1 0	–	–	–
4	1 0 0	0 0 2	–	–	1 0 0	–	–
5	–	–	–	–	1 0 0	–	1 0 1
6	1 1 0	0 0 1	0 0 1	1 1 0	–	–	2 1 2
7	–	–	–	–	1 2 2	–	–
8	–	–	0 0 3	–	–	–	1 1 1
9	2 2 0	1 1 1	0 0 1	0 1 1	–	1 2 1	1 0 1
10	–	1 1 2	0 1 1	1 1 1	0 3 3	1 2 1	–
11	–	–	1 1 2	1 1 1	0 1 1	1 2 2	1 1 1
12	4 3 2	0 1 3	1 2 2	0 3 1	1 1 1	–	–
13	1 1 1	5 4 4	1 1 1	0 1 0	1 0 0	–	–
14	–	–	2 2 6	–	–	–	–
15	1 2 4	1 1 1	1 1 1	–	1 0 0	–	–
16	0 0 2	2 2 1	0 1 1	0 2 2	1 1 1	–	–
17	–	2 1 0	–	–	2 3 4	–	–
18	0 0 1	3 3 1	0 1 1	0 0 10	0 0 10	–	–
19	–	–	5 4 2	–	–	–	–
20	–	–	–	0 0 1	–	0 0 5	1 0 1
21	0 0 2	1 2 1	2 2 2	3 3 5	0 0 2	4 6 6	3 3 3
22	–	0 1 1	0 0 1	0 0 1	8 6 9	–	–
23	1 2 5	–	–	1 1 1	–	1 2 0	–
24	0 0 1	–	0 0 1	0 0 1	1 0 0	0 1 1	2 10 10
25	1 2 2	–	–	1 0 0	1 0 0	–	–

We comment briefly on the manner in which the values of $t$ and $T$ arise for some illustrative cases. For a number of eigenvectors there is one data set which is overwhelmingly most constraining. Examples are eigenvectors 17 and 25 at NLO and 7 and 25 at NNLO. A number of these are where the constraint is from the E866/NuSea Drell–Yan ratio data, since this is one of the few data sets sensitive to the $\bar{d} -\bar{u}$ difference. In these cases the tolerance tends to be low. For the cases where the tolerance is high there are some definite examples where this is due to tension between two data sets. One of the clearest and most interesting examples is eigenvector 13 at NLO. In this case the fit to HERA $e^+p$ NC 820 GeV improves in one direction and deteriorates in the other, while the fit to NMC structure function data for $x < 0.1$ deteriorates in one direction and improves in the other. In this case the NMC data are at low $Q^2$ and the HERA data at higher $Q^2$ and the fit does not match either perfectly simultaneously. The effect is smaller at NNLO though is evident in eigenvector 3. Other cases where $t$ is high and data sets are in very significant tension are eigenvector 4 at NLO, where DØ electron and muon asymmetry compete and eigenvector 20 at NLO where CCFR and NuTeV dimuon data prefer a different high-$x$ strange quark. This complete tension is less evident in NNLO eigenvectors. However, there are some cases where one data set has deteriorating fit quality in one direction and improving quality in the other, while another data set deteriorates quickly in one direction, but varies only slowly in the other. Examples of this are eigenvectors 1 and 23 at NLO and eigenvector 1 at NNLO. Often the variation of $\chi ^2$ of all data sets is fairly slow except for one data set in one direction and a different data set in another direction. Examples of this are eigenvector 22 at NLO and eigenvectors 10, 22 and 24 at NNLO. A final type of cases is similar, but where one data set deteriorates in both directions but one other deteriorates slightly more quickly in one direction but very slowly in the other. Examples are eigenvector 4 at NNLO, where BCDMS data deteriorates in both directions but SLAC only in one direction and eigenvector 21 at NNLO, where ATLAS $W,Z$ data deteriorates in both directions, but HERA data only in one direction.

We do not show the details of the eigenvectors at LO since we regard this as a much more approximate fit. However, we note that at LO the good agreement between $t$ and $T$ breaks down much more significantly, particularly for eigenvectors with the highest few eigenvalues. This is a feature of even more tension between data sets in the LO fit, and indeed, in the NLO and NNLO fit we would regard these eigenvectors as unstable, and discount them. However, we wish to obtain a conservative uncertainty on the PDFs at LO, so keep the same number of eigenvectors as at NLO and NNLO.

We see that there is some similarity between the eigenvectors for the NLO and NNLO PDFs, with some, e.g. 1, 5, 7, 19, 20, being constrained by the same data set and corresponding to the same type of PDF uncertainty. In some cases the order of the eigenvectors (determined by size of eigenvalue) is simply modified slightly by the changes between the NLO and NNLO fit e.g. 3 at NLO and 2 at NNLO, 23 at NLO and 24 at NNLO. However, despite the fact that the data fit at NNLO is very similar to that at NLO, and the parameterisation of the input PDFs is identical, the changes in the details of the NLO and NNLO fit are sufficient to remove any very clear mapping between the eigenvectors in the two cases, and some are completely different. We present the details of the eigenvectors at NLO here for the best-fit value of $\alpha _S(M_Z^2) =0.120$. However, we also make available a NLO PDF set with $\alpha _S(M_Z^2) =0.118$ with both a central value and a full set of eigenvectors (though the fit quality is 17 units worse for this value of $\alpha _S(M_Z^2)$). It is perhaps comforting to note that there is a practically identical mapping between the NLO eigenvectors for the two values of $\alpha _S(M_Z^2)$, with the main features of PDF uncertainties being the same, without any modification of the order of the eigenvectors. The precise values of $t$ and $T$ are modified a little, and in a couple of cases the most constraining sets changed (always for one which was almost the most constraining set at the other coupling value). The uncertainties (defined by changes in $\chi ^2$ relative to the best-fit values in each case) are very similar.

#### Data sets which most constrain the MMHT2014 PDFs

It is very clear from Tables 7 and 9 that a wide variety of different data types are responsible for constraining the PDFs. At NLO 6 of the 50 eigenvector directions are constrained by HERA structure function data, 13 by fixed-target data structure function data, and 4 by the newest LHC data. Three of the LHC driven constraints are on the valence quarks and come from lepton asymmetry data. One is a constraint on the strange quark from the ATLAS $W$ and $Z$ data. There are still nine constraints from Tevatron data, again mainly on the details of the light-quark decomposition. The CCFR and NuTeV dimuon data [31] constrain eight eigenvector directions because they still provide by far the dominant constraint on the strange and antistrange quarks, which have five free parameters in the eigenvector determination. Similarly, the E866 Drell–Yan total cross section asymmetry data constrain 10 eigenvector directions mainly because the asymmetry data are still by far the best constraint on $\bar{d} - \bar{u}$, which has three free parameters.

At NNLO the picture is quite similar, but now HERA data constrain 11 eigenvector directions. Fixed-target data are similar to NLO with 10, but the Tevatron reduces to six. The LHC data now constrain eight eigenvector directions. As at NLO, this is dominantly lepton asymmetry data constraining valence quarks (winning out over Tevatron data compared to NLO in a couple of cases) but also ATLAS $W,Z$ data constrain the sea and strange sea in one eigenvector direction and $\sigma ({t \bar{t}})$ provide a constraint on the high-$x$ gluon. The dimuon and E866 Drell–Yan data provide similar constraints to NLO with nine and six, respectively, though in the latter case it is always the asymmetry data which contribute.

We do not make $90\,\%$ confidence-level eigenvectors directly available, as was done in [1], but we simply advocate an expansion of the $68\,\%$ confidence-level uncertainties by the standard factor of 1.645. This is true to a reasonably good approximation. There was not a very obvious demand for explicit $90\,\%$ confidence-level eigenvectors in the last release, and some cases where the availability of two different sets of eigenvectors led to mistakes and confusion.

#### Availability of MMHT2014 PDFs

**Fig. 20:**
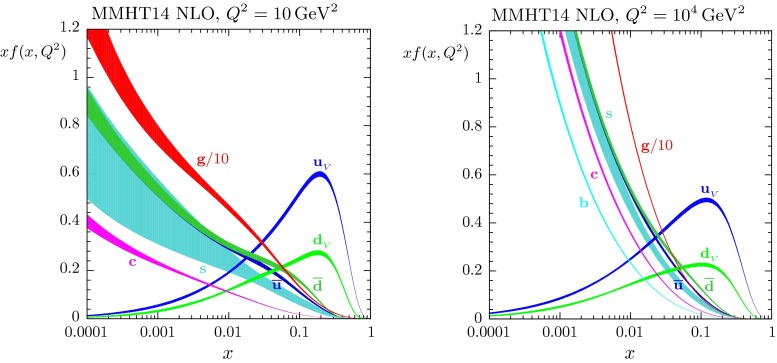
MMHT2014 NLO PDFs at $Q^2=10 ~\mathrm GeV^2$ and $Q^2=10^4 \mathrm GeV^2$, with associated 68 $\%$ confidence-level uncertainty bands. The corresponding plot of NNLO PDFs was shown in Fig. 1

Recall that the NNLO set of PDFs that we present correspond to the default value of $\alpha _S(M_Z^2)=0.118$. These NNLO PDFs at scales of $Q^2=10$ and $10^4~\mathrm GeV^2$ were shown in Fig. 1. The corresponding NLO PDFs with a default value $\alpha _S(M_Z^2)=0.120$ are shown in Fig. 20. As $Q^2$ increases we expect the uncertainties on the PDFs to decrease, particularly at very small $x$. This is well illustrated in the plots by comparing the PDFs at $Q^2=10~\mathrm GeV^2$ with those at $Q^2=10^4 ~\mathrm GeV^2$. We also make available a second set of NLO PDFs with $\alpha _S(M_Z^2)=0.118$. In addition, we provide a LO set of PDFs, which have $\alpha _S(M_Z^2)=0.135$, though these give a poorer description of the global data; see Table 5.

These four sets of PDFs are available as program-callable functions from [14], and from the LHAPDF library [15]. A new HepForge [16] project site is also expected.

Although we leave a full study of the relationship between the PDFs and the strong coupling constant $\alpha _S$ to a follow-up publication we also make available PDF sets with changes of $\alpha _S(M_Z^2)$ of 0.001 relative to the PDF eigenvector sets, i.e. at $\alpha _S(M_Z^2)=0.117$ and $0.119$ at both NLO and NNLO, and also at $\alpha _S(M_Z^2)=0.121$ at NLO. We also make sets available at $\alpha _S(M_Z^2)=0.134$ and 0.136 at LO. This is in order to enable the $\alpha _S$ variation in the vicinity of the default PDFs to be examined and for the uncertainty to be calculated if the simple procedure of addition of $\alpha _S(M_Z^2)$ errors in quadrature is applied.^14^

### Comparison of MMHT2014 with MSTW2008 PDFs

**Fig. 21:**
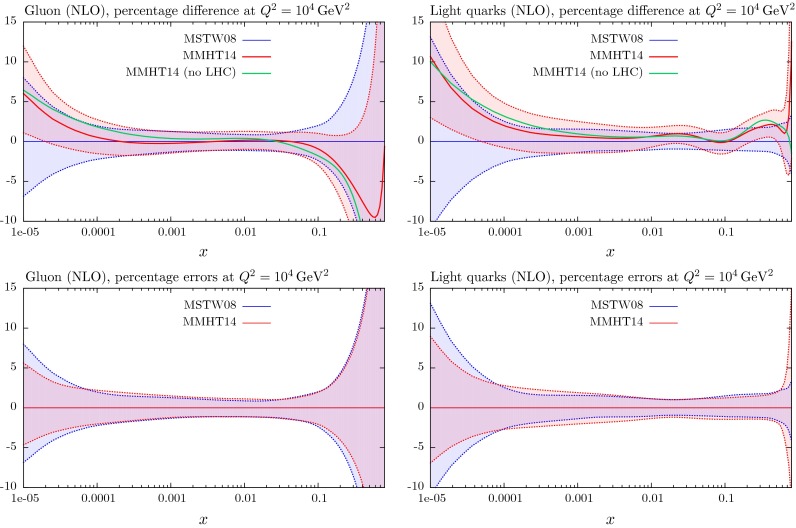
The change, in the $g$ and light-quark PDFs at NLO for $Q=10^4~\mathrm GeV^2$, in going from the MSTW values to those in the present global NLO fit, which includes the LHC data. Also shown are comparisons of the percentage errors in the two analyses

**Fig. 22:**
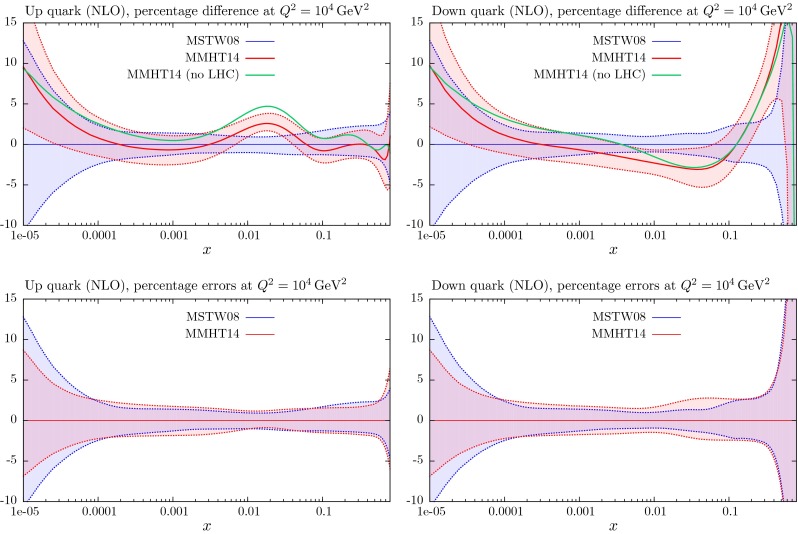
The change, in the $u$ and $d$ PDFs at NLO for $Q=10^4~\mathrm GeV^2$, in going from the MSTW values to those in the present global NLO fit, which includes the LHC data. Also shown are comparisons of the percentage errors in the two analyses

**Fig. 23:**
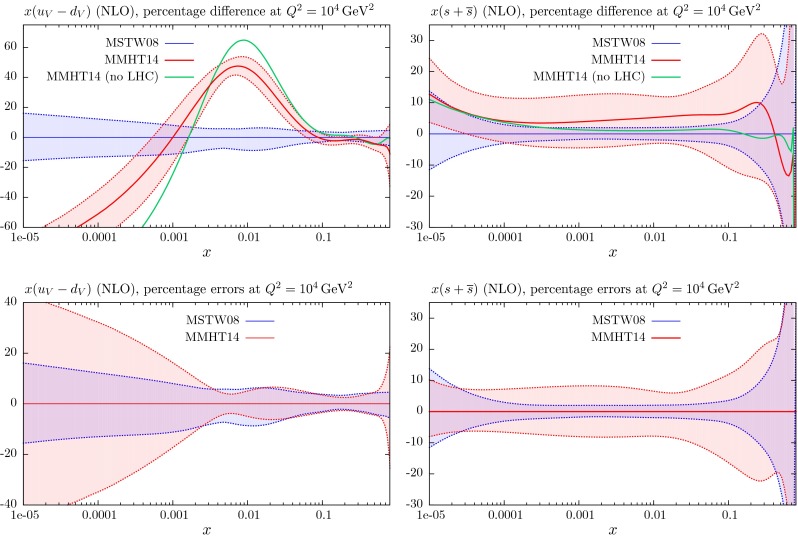
The change, in the $(u_V-d_V)$ and $(s+\bar{s})$ PDFs at NLO for $Q=10^4~\mathrm GeV^2$, in going from the MSTW values to those in the present global NLO fit, which includes the LHC data. Also shown are comparisons of the percentage errors in the two analyses

We now show the change in both the central values and the uncertainties of the NLO PDFs at $Q^2=10^4~\mathrm GeV^2$ in going from the NLO MSTW analysis. The ratio of the MMHT2014 PDFs, along with uncertainties, to the MSTW2008 PDFs is shown in Figs. 21, 22 and 23. We also show the central value of the MMHT2014 fit before LHC data are added in the top plot in each case. In the lower plots we simply compare the uncertainties of the MMHT2014 PDFs and the MSTW2008 PDFs.

#### Gluon and light quark

In Fig. 21 we compare the gluon and total light-quark distributions. In this and subsequent plots we show uncertainty bands for the full MMHT2014 and MSTW2008 PDFs, but only show the central value of the MMHT2014 PDFs obtained without LHC data. This is because it is interesting to see the (usually quite small) direct effect on the best PDFs from LHC data, but we note that the parameterisation for the strange quark is more limited when LHC data are not included as without LHC Drell–Yan type data there is insufficient constraint on the details of the shape of the strange quark. This means it is not possible to properly reflect the change in strange quark uncertainty in MMHT2014 PDFs before and after LHC data is added, which is actually the dominant change in PDF uncertainties between MSTW2008 and MMHT2014 PDFs, and which feeds into the total light-quark uncertainty. Really, it is only the addition of the LHC data which allow us to present an uncertainty on the strange PDFs with full confidence. We do note, however, that the gluon uncertainty is essentially unchanged by the addition of LHC data except to a very minor improvement at high-$x$ at NLO.

The change in the central value of the gluon is almost the same with and without LHC data. It is slightly softer at high $x$ and a little larger at the smallest $x$ values shown, but within uncertainties, particularly when the LHC data are included. This slight change in shape is due to the inclusion of the combined HERA data, as indicated in [135]. However, the slight softening at high $x$ is also exhibited when the default heavy flavour scheme is replaced by the optimal scheme in [34] and when LHC jet data are included in [109]. Hence, it seems that a variety of new effects all prefer this slight change in shape, but even the combination of all of them only results in a small change. The gluon and light-quark uncertainty decreases a little at lowest $x$, due to the combined HERA data, and the gluon uncertainty decreases very slightly at $x > 0.1$ due to inclusion of LHC jet data. The light sea is a little larger at the smallest $x$, driven by the same shape change in the gluon distribution and the evolution. We note that there are few data for $x < 10^{-4}$, but there is some, which acts to constrain the small-$x$ sea. There is less direct constraint on the gluon at very small $x$ and $Q^2$, though still some from $\mathrm{d}F_2(x,Q^2)/\mathrm{d}\ln \, Q^2$ and $F_L(x,Q^2)$ and the uncertainty is very large. However, at much higher $Q^2$ most of the gluon and light sea at $x=10^{-5}$ is determined by evolution from higher $x$, and even a very large uncertainty at input is largely washed out by this.

The changes in detailed shape at high $x$ are mainly due to individual quark flavour contributions and will be discussed below. The uncertainty is reduced for $x< 0.0001$, mirroring the same effect in the gluon. The increase in uncertainty at very high $x$ is due to the improved parameterisation flexibility. The slight increase in uncertainty over a wide range of $x$ is due to the large uncertainty introduced into the branching ratio, $B_{\mu }$, for charmed mesons decaying to muons (as discussed in Sect. 2.6), which increases the strange quark uncertainty and hence that of the entire light sea.

#### Up and down quark

In Fig. 22 we compare the up and down quark distributions. The very small $x$ increase has already been explained, and is common to all quarks. The increase around $x=0.01$ compared to MSTW2008 was already apparent in [11], and is due to the improved parameterisation (and to some extent improved deuteron corrections) and the increase is mainly in the up valence distribution. The increase is very compatible with fitting ATLAS and CMS data on $W^{\pm }$ production at low rapidity, but is not actually driven by this at all. In fact, we see that the increase is actually significantly larger before the inclusion of LHC data. The down quark has changed shape quite clearly. The decrease for $x\sim 0.05$ and increase at high $x$ was again already apparent in [11] and is due to improved deuterium corrections and parameterisation. The fine details are modified by the inclusion of LHC data, but the main features are present in the fit without LHC data. The change in the uncertainties is similar to that for the total light sea, though the flexibility in the improved deuteron corrections does contribute to the increase in uncertainty of the down distribution.

#### $u_V-d_V$ and $s+\bar{s}$ distributions

In Fig. 23 we compare the $u_V(x,Q^2)-d_V(x,Q^2)$ and $s(x,Q^2) + \bar{s}(x,Q^2)$ distributions. The very dramatic change in the former was already seen in [11]. In fact Ref. [11] was able to give a reasonable description of the observed lepton charge asymmetry at the LHC, whereas MSTW2008 gave a poor prediction. This is really the only blemish of the MSTW2008 [1] predictions. The change in $u_V-d_V$ for $x\lesssim 0.03$ is very evident in the figure. This change is not driven by the LHC data, but rather by the improved flexibility of the MMHT (and MMSTWW [11]) parameterisations (and improved deuteron corrections). Indeed, as seen with the up quark, the change, from the MSTW2008 partons, is larger before the inclusion of LHC data. The uncertainty in $u_V(x,Q^2)-d_V(x,Q^2)$ increases very significantly at small $x$ due to the increased flexibility of the MMHT parameterisation. However, there is a decrease near $x=0.01$ due to the constraint added by the LHC asymmetry data, which is the only real change compared to the MMSTWW distribution.

There is a very significant increase in the uncertainty in the $s+\bar{s}$ distribution (at all but the lowest $x$ where the distribution is governed mainly by evolution from the gluon), due mainly to the freedom allowed for the branching fraction $B_{\mu }$, see Sect. 2.6, though there is also one more free parameter for this PDF in the eigenvector determination. The central value of the total strange distribution is very similar to MSTW2008 before LHC data are included, with only the common slight increase at lowest $x$. This is despite the correction of the theoretical calculation of dimuon production and a change in nuclear corrections, showing the small impact of these two effects (though they do actually tend to pull in opposite directions). There is a few percent increase when the LHC data are included, mainly driven by the ATLAS $W,Z$ data. The central value is outside the uncertainty band of the MSTW2008 distribution. However, the MSTW2008 distribution is included comfortably within the error band of the MMHT2014 distribution.

#### $\bar{d} - \bar{u}$ and $s-\bar{s}$ distributions

**Fig. 24:**
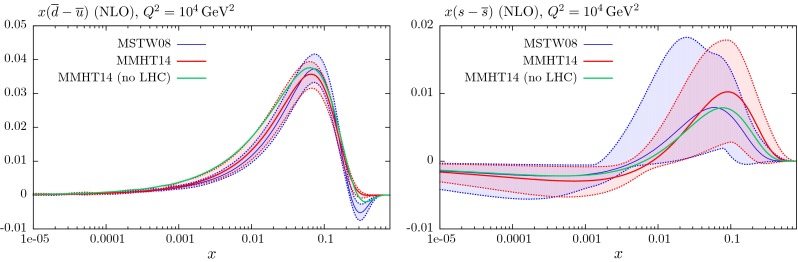
The change, in the $(\bar{d} -\bar{u})$ and $(s-\bar{s})$ PDFs at NLO for $Q=10^4~\mathrm GeV^2$, in going from the MSTW values to those in the present global NLO fit, which includes the LHC data

In Fig. 24 we show the comparison of $\bar{d}(x,Q^2)-\bar{u}(x,Q^2)$ and $s(x,Q^2) - \bar{s}(x,Q^2)$. In this case showing the percentage uncertainties is not useful, due to the fact that both distributions pass through zero. One can see that there is no very significant change in either the central values or uncertainties. There is a fairly distinct tendency for $\bar{d}(x,Q^2)-\bar{u}(x,Q^2)$ to be negative for $x\sim 0.3$ in the MSTW2008 set, which may be a sign of the overall more restricted parameterisation in this case, but other than this the MSTW2008 and MMHT2014 $\bar{d}(x,Q^2)-\bar{u}(x,Q^2)$ distributions are very consistent. This is unsurprising as the dominant constraint is still the E866/NuSea Drell–Yan ratio data [89]. The MMHT2014 $s(x,Q^2) - \bar{s}(x,Q^2)$ distribution has a tendency to peak at slightly higher $x$, but the MSTW2008 and MMHT2014 distributions are very consistent and have similar size uncertainties. The main constraint is still overwhelmingly the CCFR and NuTeV $\nu N\rightarrow \mu \mu X$ data [31], and the change in the treatment of the branching ratio has little effect on the asymmetry. There is some small constraint from $W$ asymmetry data, and the new data from the LHC provides some pull, and contributes to the MMHT2014 uncertainty being a little smaller for $x<0.05$. This constraint will improve in the future.

#### Comparison with MSTW2008 at NNLO

**Fig. 25:**
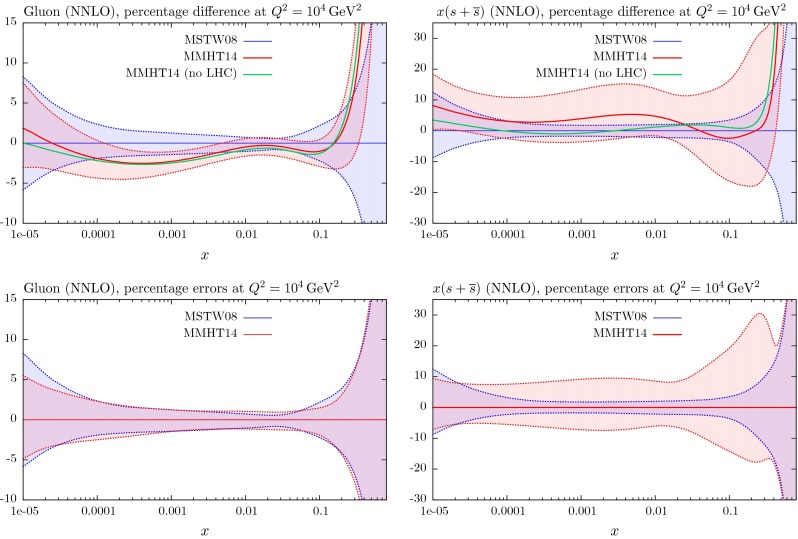
The change, in the $g$ and $s+\bar{s}$ PDFs at NNLO for $Q=10^4~\mathrm GeV^2$, in going from the MSTW values to those in the present global NNLO fit, which includes the LHC data. Also shown are comparisons of the percentage errors in the two analyses

The changes in the NNLO PDFs going from MSTW2008 to MMHT2014 are very similar to those at NLO. However, the $g$ and $s+\bar{s}$ changes are shown in Fig. 25. The gluon has now become a little harder at high $x$ and a bit smaller between $x=0.0001$ and $x=0.01$. The slight decrease in the NNLO gluon between $x=0.0001$ and $x=0.01$ (which, via evolution, shows up to some extent in the sea quarks) is driven largely by the fit to the combined HERA data, while the increase at very high $x$ is related to the use of multiplicative uncertainties for the Tevatron jet data, and by the momentum sum rule. The change in the MMHT2014 $s +\bar{s}$ distribution is similar to that at NLO, except that there is a slight decrease near $x=0.1$ as opposed to an increase at all $x$. This is due to a slightly larger correction to the dimuon cross section in this region at NNLO than at NLO, but also, this seems to be the preferred shape to fit the ATLAS $W,Z$ data at NNLO.

**Fig. 26:**
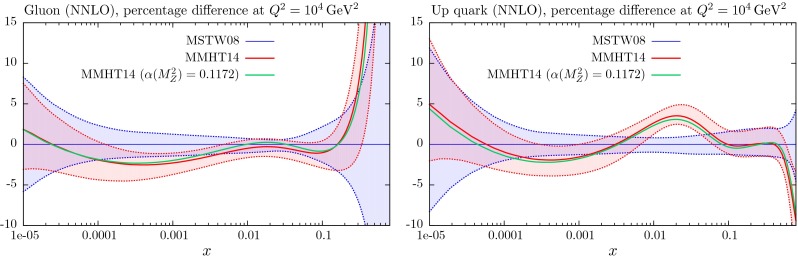
The change in the $g$ and $u$ PDFs at NNLO for $Q=10^4~\mathrm GeV^2$, in going from the MSTW values to those in the present global NNLO fit [with default $\alpha _S(M_Z^2)=0.118$], which includes the LHC data. Also shown is the NNLO fit for the optimal value $\alpha _S(M_Z^2)=0.1172$

Part of the change in the gluon distribution is due to the fact that the MMHT2014 PDFs were defined at $\alpha _S(M_Z^2)=0.118$ while the MSTW2008 PDFs are defined at $\alpha _S(M_Z^2)=0.1171$. Recall that the gluon increases at very high $x$ and decreases at lower $x$ with an increase in $\alpha _S(M_Z^2)$, as seen in Fig. 11(f) of [136]. However, this is responsible for only a relatively minor part of the total difference between the MMHT2014 and MSTW2008 NNLO gluon distributions. The gluon distribution for the MMHT optimal fit value of $\alpha _S(M_Z^2)=0.1172$ is shown in Fig. 26. As one sees the gluon for $\alpha _S(M_Z^2)=0.1172$ is much closer to the MMHT2014 gluon (for the default $\alpha _S(M_Z^2)=0.118$) than to the MSTW2008 gluon, and is always well within the uncertainty band. For the up and down quark distributions the difference between the results for the default value $\alpha _S(M_Z^2)=0.118$ and the optimal $\alpha _S(M_Z^2)=0.1172$ at $Q^2=10^4~\mathrm GeV^2$ agree to within $0.5\,\%$ for all $0.0001<x<0.6$, as one can also see in Fig. 26. We also see, by comparing to Fig. 22, that the change in the up quark distribution in going from MSTW2008 to MMHT2014 is indeed very similar at NNLO to that at NLO.

Just as at NLO, the only real impact on the quark uncertainties due to the LHC data is a slight improvement in the flavour decomposition near $x=0.01$. However, the fact that LHC jet data is absent at NNLO means the very slight reduction in uncertainty in the high-$x$ gluon due to the inclusion of LHC data is absent at NNLO.

**Fig. 27:**
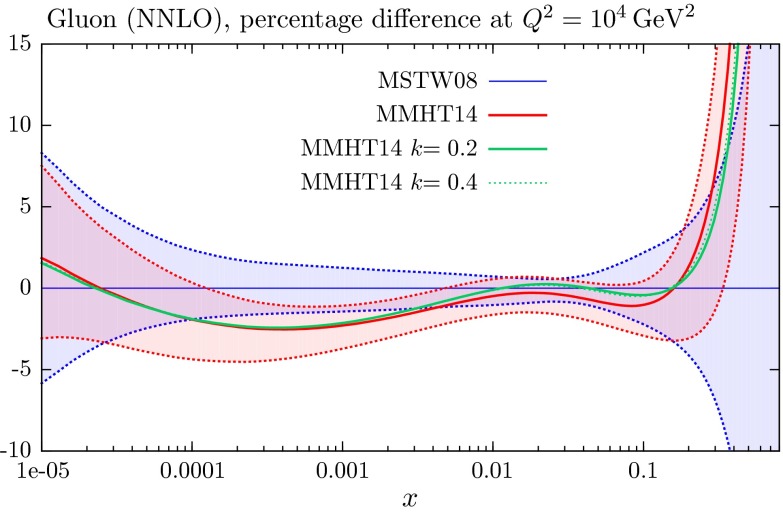
The change in the $g$ PDF at NNLO for $Q=10^4~\mathrm GeV^2$, in going from the MSTW values to those in the present global NNLO fit, which includes the LHC data. Also shown are the central values of the change in the $g$ PDF in the NNLO fits where LHC jet data are included with both the larger and smaller approximate $K$-factors; these *two curves* are almost indistinguishable from each other

We also show the effect of including the LHC jet data in the NNLO fit with the use of both the smaller and larger $K$-factors described in Sect. 4.3.1. In both fits the preferred value of $\alpha _S(M_Z^2)$ is close to $0.1172$. The resulting gluon distribution in each case is shown in Fig. 27. One can see that the change in the gluon is very small (indeed it is very similar to that in the $\alpha _S(M_Z^2)=0.1172$ fit, as can be seen by comparing with Fig. 26) and fairly insensitive to the overall size of the $K$-factor. As was seen in Fig. 18, a relatively smooth and moderately sized correction to theory can be largely accommodated by a larger shift of data compared to theory using correlated systematics, with little, if any extra penalty. As noted in Sect. 4, however, this is not as easy to do with jet data taken at two different energy scales, and it will also not be as successful with reduced correlated systematic uncertainties.

#### Comparison between NLO and NNLO

**Fig. 28:**
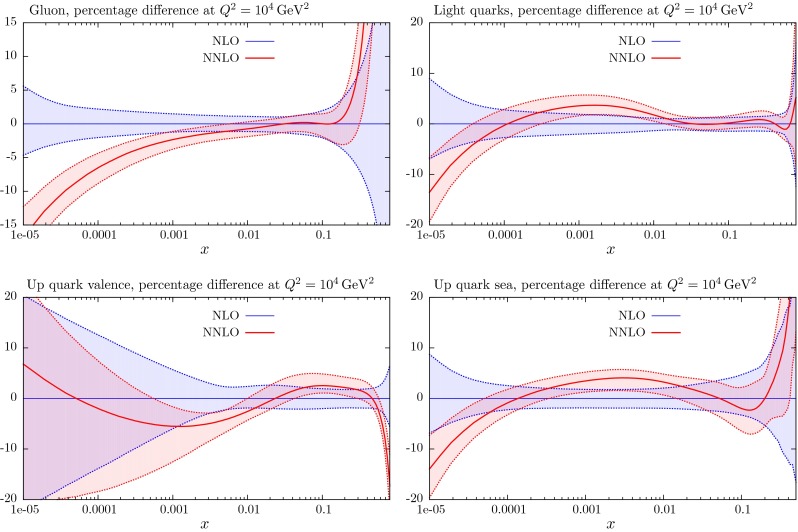
The comparison between the NLO and NNLO $g$, light quark, $u_V$ and $\bar{u}$ PDFs for $Q=10^4~\mathrm GeV^2$

The comparison between some of the NLO and NNLO PDFs is shown in Fig. 28. One can see that the NNLO gluon is a little higher at highest $x$ and becomes smaller at the lowest $x$ values. The latter effect may be understood as being due to the slower evolution of the gluon at very small $x$ at NNLO as a consequence of the correction to the splitting function. This is mirrored in the very small-$x$ behaviour of the light quarks and the up sea quark, where the evolution is driven by the gluon. The change in shape of $u_V$ between NLO and NNLO is a consequence of the NNLO non-singlet coefficient function which is positive at very large $x$, leading to fewer quarks, and then becomes negative near $x=0.1$, leading to more valence quarks. The effect at high $x$ is less clear in the $d_V$ distribution due to the freedom for the deuteron correction to be different at NNLO than at NLO. The sea quark is larger at NNLO for all $x<0.1$ until the lowest values. This is due to a negative NNLO structure function coefficient function in this region, which means the fit to data requires more sea quarks. The shape is common to all light sea quarks, not just $\bar{u}$. This is also evident in the change in the light-quark distribution. The heavy quarks are generated almost entirely by evolution from the gluon, so their shape change is extremely similar to that of the gluon. The uncertainties at NLO and NNLO are very similar to each other, depending primarily on the uncertainties in the data.

## Predictions and benchmarks

In Tables 11 and 12 we show the predictions for various benchmark processes at the LHC for the MSTW PDFs [1] and the MMHT sets of PDFs, also showing the results before LHC data are included in the fit for comparison (though the uncertainties are not calculated in this case). We calculate the total cross sections for $Z\rightarrow l^+l^-$, $W\rightarrow l \nu$, Higgs production via gluon–gluon fusion and $t \bar{t}$ production. For $W, Z$ and Higgs production we use the same approach to calculation as used^15^ in [1], and improved in [132]. For the $Z\rightarrow l^+l^-$ branching ratio we use 0.033658 and for the $W\rightarrow l \nu$ we take 0.1080 [129]. We use LO electroweak perturbation theory, with the $qqW$ and $qqZ$ couplings defined by

37 $$\begin{aligned} g_W^2 = G_F M_W^2 / \sqrt{2}, \quad g_Z^2 = G_F M_Z^2 \sqrt{2}, \end{aligned}$$

and other electroweak parameters are as in [1]. We take the Higgs mass to be $m_H=125~\mathrm GeV$, and the top pole mass $m_t=172.5~\mathrm GeV$. For the $t \bar{t}$ cross section we use the calculation and code in [104]. In all cases we use the particle mass as the renormalisation and factorisation scale. The main purpose of the presentation is to investigate how both the central values and the uncertainties of the predictions have changed in going from MSTW2008 PDFs to MMHT2014 PDFs, so we provide results for the Tevatron and LHC with centre-of-mass energies 7 and 14  TeV. This gives quite a spread of energies whereas relative effects at 8 and 13 TeV would be very similar to those at 7 and 14  TeV. We do not intend to present definite predictions or compare in detail to other PDF sets as both these results are frequently provided in the literature with very specific choices of codes, scales and parameters which may differ from those used here.

**Table 11:** The values of various cross sections (in nb) obtained with the NLO MSTW 2008 parton sets [1] and the NLO MMHT 2014 sets. We show the values before and after the LHC data are included in the present fits, but not the uncertainty in the former case. The uncertainties are PDF uncertainties only

	MSTW08 NLO	MMHT14 NLO no LHC	MMHT14 NLO
$W\,\, \mathrm{Tevatron}\,\,(1.96~\mathrm TeV)$	$2.659^{+0.057}_{-0.045}$	2.685	$2.645^{+0.058}_{-0.049}$
$Z \,\,\mathrm{Tevatron}\,\,(1.96~\mathrm TeV)$	$0.2426^{+0.0054}_{-0.0043}$	0.2486	$0.2442^{+0.0049}_{-0.0043}$
$W^+ \,\,\mathrm{LHC}\,\, (7~\mathrm TeV)$	$5.960^{+0.129}_{-0.097}$	6.107	$5.974^{+0.092}_{-0.086}$
$W^- \,\,\mathrm{LHC}\,\, (7~\mathrm TeV)$	$4.192^{+0.092}_{-0.071}$	4.181	$4.163^{+0.069}_{-0.061}$
$Z \,\,\mathrm{LHC}\,\, (7~\mathrm TeV)$	$0.931^{+0.020}_{-0.014}$	0.941	$0.932^{+0.013}_{-0.013}$
$W^+ \,\,\mathrm{LHC}\,\, (14~\mathrm TeV)$	$12.07^{+0.24}_{-0.21}$	12.43	$12.17^{+0.20}_{-0.18}$
$W^- \,\,\mathrm{LHC}\,\, (14~\mathrm TeV)$	$9.107^{+0.19}_{-0.16}$	9.16	$9.10^{+0.15}_{-0.14}$
$Z \,\,\mathrm{LHC}\,\, (14~\mathrm TeV)$	$2.001^{+0.040}_{-0.032}$	2.035	$2.016^{+0.031}_{-0.033}$
$\mathrm{Higgs} \,\,\mathrm{Tevatron}$	$0.658^{+0.021}_{-0.027}$	0.636	$0.644^{+0.021}_{-0.022}$
$\mathrm{Higgs} \,\,\mathrm{LHC}\,\,(7~\mathrm TeV)$	$11.39^{+0.16}_{-0.19}$	11.26	$11.28^{+0.21}_{-0.20}$
$\mathrm{Higgs} \,\,\mathrm{LHC}\,\,(14~\mathrm TeV)$	$37.93^{+0.42}_{-0.60}$	37.67	$37.63^{+0.67}_{-0.59}$
$t\bar{t} \,\,\mathrm{Tevatron}$	$6.85^{+0.19}_{-0.13}$	6.89	$6.82^{+0.18}_{-0.17}$
$t\bar{t}\,\,\mathrm{LHC}\,\,(7~\mathrm TeV)$	$162.0^{+4.3}_{-5.4}$	157.0	$158.6^{+4.5}_{-4.5}$
$t\bar{t}\,\,\mathrm{LHC}\,\,(14~\mathrm TeV)$	$903.8^{+16}_{-17}$	886.7	$891.9^{+18}_{-18}$

**Table 12:** The values of various cross sections (in nb) obtained with the NNLO MSTW 2008 parton sets [1] and the NNLO MMHT 2014 sets. We show the values before and after the LHC data are included in the present fits, but not the uncertainty in the former case. The uncertainties are PDF uncertainties only

	MSTW08 NNLO	MMHT14 NNLO no LHC	MMHT14 NNLO
$W\,\, \mathrm{Tevatron}\,\,(1.96~\mathrm TeV)$	$2.746^{+0.049}_{-0.042}$	2.803	$2.782^{+0.056}_{-0.056}$
$Z \,\,\mathrm{Tevatron}\,\,(1.96~\mathrm TeV)$	$0.2507^{+0.0048}_{-0.0041}$	0.2574	$0.2559^{+0.0052}_{-0.0046}$
$W^+ \,\,\mathrm{LHC}\,\, (7~\mathrm TeV)$	$6.159^{+0.111}_{-0.099}$	6.214	$6.197^{+0.103}_{-0.092}$
$W^- \,\,\mathrm{LHC}\,\, (7~\mathrm TeV)$	$4.310^{+0.078}_{-0.069}$	4.355	$4.306^{+0.067}_{-0.076}$
$Z \,\,\mathrm{LHC}\,\, (7~\mathrm TeV)$	$0.9586^{+0.020}_{-0.014}$	0.9695	$0.9638^{+0.014}_{-0.013}$
$W^+ \,\,\mathrm{LHC}\,\, (14~\mathrm TeV)$	$12.39^{+0.22}_{-0.21}$	12.49	$12.48^{+0.22}_{-0.18}$
$W^- \,\,\mathrm{LHC}\,\, (14~\mathrm TeV)$	$9.33^{+0.16}_{-0.16}$	9.39	$9.32^{+0.15}_{-0.14}$
$Z \,\,\mathrm{LHC}\,\, (14~\mathrm TeV)$	$2.051^{+0.035}_{-0.033}$	2.069	$2.065^{+0.035}_{-0.030}$
$\mathrm{Higgs} \,\,\mathrm{Tevatron}$	$0.853^{+0.028}_{-0.029}$	0.877	$0.874^{+0.024}_{-0.030}$
$\mathrm{Higgs} \,\,\mathrm{LHC}\,\,(7~\mathrm TeV)$	$14.40^{+0.17}_{-0.23}$	14.54	$14.56^{+0.21}_{-0.29}$
$\mathrm{Higgs} \,\,\mathrm{LHC}\,\,(14~\mathrm TeV)$	$47.50^{+0.47}_{-0.74}$	47.61	$47.69^{+0.63}_{-0.88}$
$t\bar{t} \,\,\mathrm{Tevatron}$	$7.19^{+0.17}_{-0.12}$	7.54	$7.51^{+0.21}_{-0.20}$
$t\bar{t}\,\,\mathrm{LHC}\,\,(7~\mathrm TeV)$	$171.1^{+4.7}_{-4.8}$	176.5	$175.9^{+3.9}_{-5.5}$
$t\bar{t}\,\,\mathrm{LHC}\,\,(14~\mathrm TeV)$	$953.3^{+16}_{-18}$	969.0	$969.9^{+16}_{-20}$

For the NLO PDFs one can see that there are no shifts in $W$ or $Z$ cross sections as large as the uncertainties when going from the MSTW2008 predictions to those of MMHT2014. The NLO values of the cross section for $Z$ production at the Tevatron and of $W^+$ production at the LHC do change by slightly more than one standard deviation on the non-LHC MMHT2014 fit, but the inclusion of LHC data brings these cross sections back towards the MSTW2008 predictions. The uncertainties are generally slightly smaller when using the MMHT2014 PDFs, but this is a fairly minor effect. For Higgs production via gluon–gluon fusion at NLO the changes are all within one standard deviation, with a slight decrease in the MMHT2014 sets due to the slightly smaller high-$x$ gluon distribution. The uncertainties are slightly decreased with the new PDFs at low energy, but increase a little at higher energy. For $t \bar{t}$ production there is a slight decrease in the predicted cross section for the MMHT2014 set at the LHC, and as with Higgs production this is more of an effect before LHC data are included. As with Higgs production this is due mainly to the smaller gluon at high-$x$, with $\sigma _{\bar{t} t}$ probing higher $x$ than Higgs production.

The trend is the same for the predictions for $$W$$ and $$Z$$ cross sections at NNLO. There is generally a slight increase from the use of the MMHT2014 sets, but, with the marginal exception of $$Z$$ production at the Tevatron, this change is always within one standard deviation for the full MMHT2014 PDFs. It is sometimes slightly more than this when using the non-LHC data MMHT2014 sets, and again the inclusion of LHC data brings MMHT2014 closer to MSTW2008. For the Higgs cross sections via gluon–gluon fusion there is consistently a very small increase. This is because even though the gluon distribution decreases in the most relevant $$x$$ region, i.e. $$x \approx 0.06$$ for $$\sqrt{s}=1.96~\mathrm TeV$$ and i.e. $$x \approx 0.009$$ for $$\sqrt{s}=14~\mathrm TeV$$, the coupling constant has increased, and this slightly overcompensates the smaller gluon. If the predictions are made using the absolutely best-fit PDFs with $$\alpha _S(M_Z^2)=0.1172$$ the Higgs predictions decrease compared to MSTW2008, but again by much less than the uncertainty. As at NLO the MMHT2014 uncertainties have reduced a little at the highest energies but increased at higher energies. For $$t \bar{t}$$ production there is an increase in the cross section for the MMHT PDFs of about $$4$$–$$5\,\%$$ at the Tevatron and $$2$$–$$3\,\%$$ at the LHC, with again the effect being slightly larger before LHC data are included. This is partially due to the larger coupling in the MMHT sets, with the change being reduced to about $$3\,\%$$ at the Tevatron and $$1$$–$$2\,\%$$ if the MMHT2014 absolute best-fit set with $$\alpha _S(M_Z^2)=0.1172$$ is used. The remainder of the effect is due to the enhancement of the very high$$-x$$ gluon at NNLO in MMHT2014. The change is in some cases more than one standard deviation from the best MSTW prediction, but only when compared to just the PDF uncertainties. If predictions with common $$\alpha _S(M_Z^2)$$ are compared, or PDF $$+$$$$\alpha _S(M_Z^2)$$ uncertainties taken into account the changes are at most about one standard deviation.

## Other constraining data: dijet, $W+c$, differential $t \bar{t}$

As well as improvements in the type of data we currently include in the PDF analysis there are currently a variety of new forms of LHC data being released, which will also provide new, sometimes complementary, constraints on PDFs. Some of the most clear examples of these are dijet data [106, 107, 140], top quark differential distributions [141, 142] and $W^-+c$ (and $W^++\bar{c}$) production [143, 144]. The first two should help constrain the high-$x$ gluon and the last is a direct constraint on the strange quark distribution. None of these have been included in our current analysis, either because suitably accurate data satisfying our cut-off on the publication date, was not available or because there is some limitation in the theoretical precision, or both. Nevertheless, we briefly comment on the comparison with each set of data.

### Dijet production at the LHC

The comparison to the dijet data in [106, 107] was studied in [109]. It was clear that at high rapidity there was a significant difference in conclusions depending on which scale choice was used, i.e. one depending just on $p_T$ or one with rapidity dependence as well. There is also double counting between the events included in the inclusive and the dijet data. In [140] the data are limited to relatively low rapidity, and full account of correlations between data sets is taken. The analysis in [140] shows that for the full data sample MSTW2008 PDFs fit extremely well, better than most alternatives, and, as seen in this article, there should be little change if the MMHT2014 PDFs are used. We will include appropriate dijet data samples in the future. However, we will probably wait for the complete NNLO formulae for the cross sections to become available, before including them in the NNLO analysis. We also note that MSTW2008 PDFs give an excellent description of the higher luminosity $7~\mathrm TeV$ ATLAS jet data [145], so presumably MMHT2014 PDFs will as well.

### $W+$ charm jet production

We also compare to the CMS [144] $W$ plus charm jet data with total cross section on $W$ plus charm jets, satisfying $p_T^\mathrm{jet} > 25$ GeV and $|\eta ^\mathrm{jet}|<2.5$, for two values of the cut on the $W$ decay lepton: $p_T^\mathrm{lep} > 25$ GeV and $p_T^\mathrm{lep} > 35$ GeV. The results are shown in Table 13 for the total $W+c$ cross section and for the ratio $R_c^{\pm } \equiv \sigma (W^+ + \bar{c} +X)/\sigma (W^- +c+X)$. The predictions are calculated using MCFM, and we get completely consistent results with the data in [144] when using the NNLO MSTW 2008 PDFs and $m_c=1.5~\mathrm GeV$. However, since the cross section is calculated at NLO, we use NLO PDFs, and we take our default mass to be $m_c=1.4~\mathrm GeV$. (This change in mass increases the cross sections by about $1\,\%$, though a little more in the lower than the higher $p_T^\mathrm{lep}$ bin.) The cross sections are then slightly larger than quoted in [144], but still below the data. The ratio of $c$ to $\bar{c}$ production is slightly lower than the data, but consistent. When using MMHT2014 the cross sections increase by a few percent, and they are actually slightly larger than the data, though well within the data uncertainty. The PDF uncertainty in the cross section is now very much larger, reflecting the increase in the uncertainty on the total $s + \bar{s}$ production. The ratios are slightly lower, and the uncertainty is very similar to that with MSTW2008, reflecting the fact that the uncertainty on $s -\bar{s}$ is essentially unchanged. The ATLAS measurements [143] are not corrected to the parton level, so cannot be directly compared. However, they appear to be a few percent higher than the CMS measurements. This is in reasonable disagreement with MSTW2008 PDFs, but appears very likely to be fully consistent with MMHT2014 PDFs. The ratio, where non-perturbative corrections presumably largely cancel, is close to 0.90, so is again likely to be very compatible with the MMHT2014 prediction.

**Table 13:** The values of the total $W+c$ cross section (in pb), and the $W^+/W^-$ ratio $R_c^{\pm }$, measured by CMS [144], compared with the predictions obtained using MSTW2008 and MMHT2014 NLO PDFs. The charm jet is subject to the acceptance cuts $p_T^\mathrm{jet} > 25$ GeV and $|\eta ^\mathrm{jet}|<2.5$

	GeV	Data	MSTW2008	MMHT2014
$\sigma (W + c)$	$p_T^\mathrm{lep}>25$	$107.7 \pm 3.3\, (\mathrm{stat.}) \pm 6.9 \,(\mathrm{sys.})$	$102.8 \pm 1.7$	$110.2 \pm 8.1$
$\sigma (W + c)$	$p_T^\mathrm{lep}>35$	$84.1 \pm 2.0 \,(\mathrm{stat.}) \pm 4.9 \,(\mathrm{sys.})$	$80.4 \pm 1.4$	$86.5 \pm 6.5$
$R^{\pm }_c$	$p_T^\mathrm{lep}>25$	$0.954 \pm 0.025 \,(\mathrm{stat.}) \pm 0.004 \,(\mathrm{sys.})$	$0.937 \pm 0.029$	$0.924 \pm 0.026$
$R^{\pm }_c$	$p_T^\mathrm{lep}>35$	$0.938 \pm 0.019 \,(\mathrm{stat.}) \pm 0.006 \,(\mathrm{sys.})$	$0.932\pm 0.030$	$0.904 \pm 0.027$

### Differential top-quark-pair data from the LHC 

**Fig. 29:**
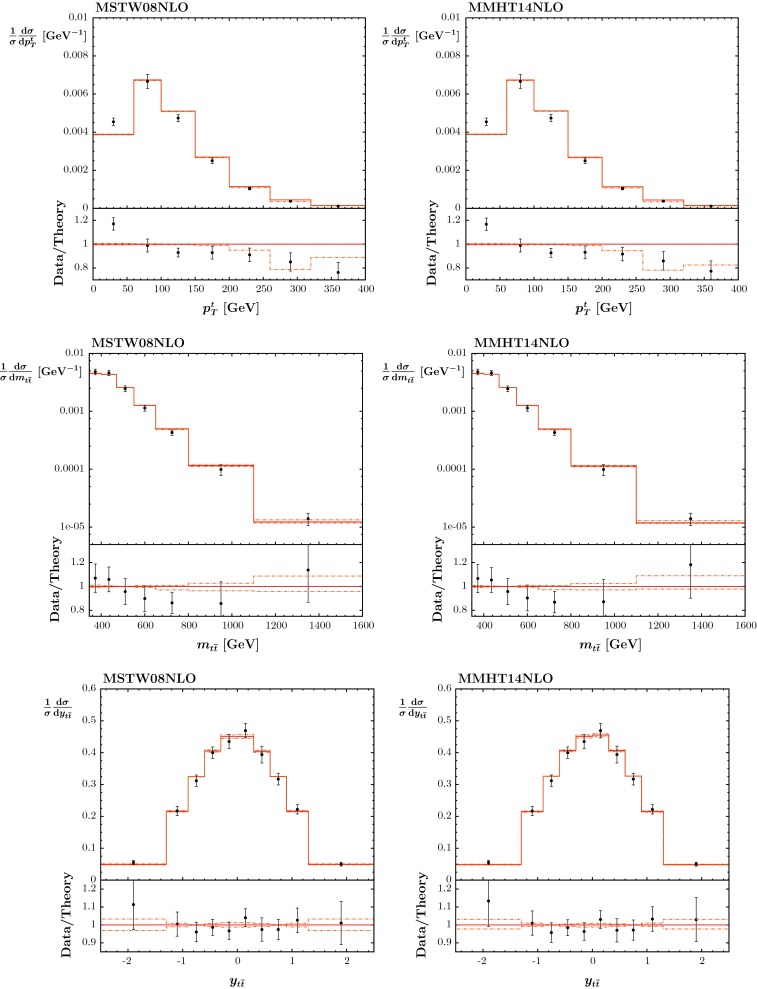
The CMS differential top quark data as functions of $p_T^t$ (*top pair of plots*), of $m_{t\bar{t}}$ (*middle plots*), and of $y_{t\bar{t}}$ (*bottom plots*), compared to the predictions of the MSTW2008 PDFs (*left*) and MMHT2014 PDFs (*right*). The *dotted lines* represent the PDF uncertainties

Finally we compare to some recent differential top quark data [142]. The comparison between NLO theory, with the calculation performed using MCFM [146], and data are shown for both MSTW2008 and MMHT2014 in Fig. 29 as functions of $p_T^t$, of $m_{t \bar{t}}$ and of $y_{t\bar{t}}$. One can see that the $p_T^t$ distribution of the data falls more quickly than the prediction. The same is arguably true, to a lesser extent, for the $m_{t \bar{t}}$ distribution, except for the last point, while the rapidity distribution matches data very well. The same trend is true for the other ‘top’ data sets. However, there is an indication [147] that NNLO corrections soften the $p^t_T$ distribution in particular, so the relatively poor comparison may be due mainly to the missing higher-order corrections.

## Comparison of MMHT with other available PDFs

Here we compare the MMHT14 PDFs to PDF sets obtained by other groups. The most direct comparison is with the NNPDF3.0 PDFs which have very recently been obtained in a new global analysis performed by the NNPDF collaboration [17]. This involves a fit to very largely the same data sets, including much of the available LHC data, and also uses a general mass variable flavour number scheme which has been shown to converge with that used in our analysis as the order increases [148]. There do, however, remain some significant differences in the two theoretical approaches. For example, NNPDF3.0 does not apply deuteron and heavy-nuclear target corrections. Moreover, the MMHT and NNPDF collaborations use quite a different procedure for the analysis. The NNPDF collaboration combine a Monte Carlo representation of the probability measure in the space of PDFs with the use of neural networks to give a set of unbiased input distributions. On the other hand, here, we use parameterisations of the input distributions based on Chebyshev polynomials where the optimum order of the polynomials for the various PDFs is explored in the fit.

Although the most direct comparison is between the MMHT14 and NNPDF3.0 sets of PDFs, we also compare to older PDF sets; i.e. the MSTW08 [1] and NNPDF2.3 [3] sets, which MMHT14 and NNPDF3.0 supersede, and with the ABM12 [5], CT10 [2] and HERAPDF1.5 [4] sets which are obtained from a smaller selection of data.^16^

### Representative comparison plots of various PDF sets

**Fig. 30:**
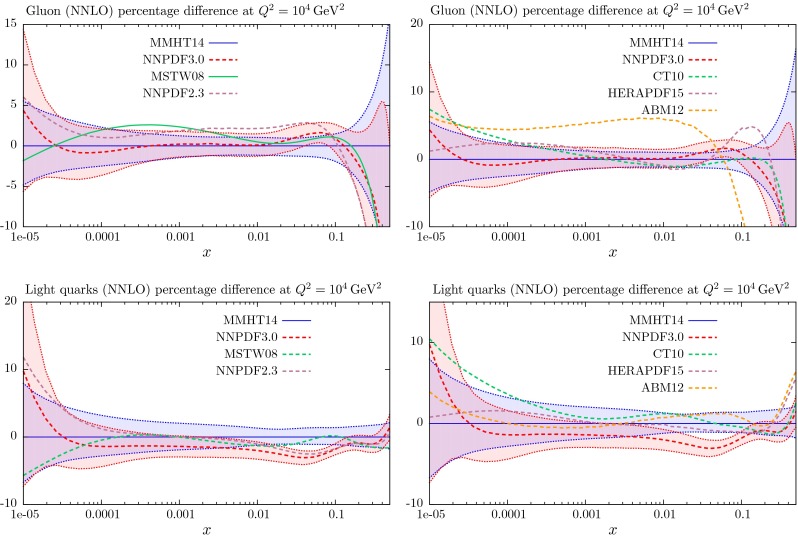
The comparison between NNLO NNPDF3.0 and MMHT14 PDFs at $Q^2=10^4~\mathrm GeV^2$ showing the $g$ and light-quark PDFs. Also shown (without error corridors, which would be similar to those of the newer sets in most cases) are the NNPDF2.3 and MSTW08 PDFs (*left*) which they supersede and (*right*) CT10 HERAPDF1.5 and ABM12 PDFs

**Fig. 31:**
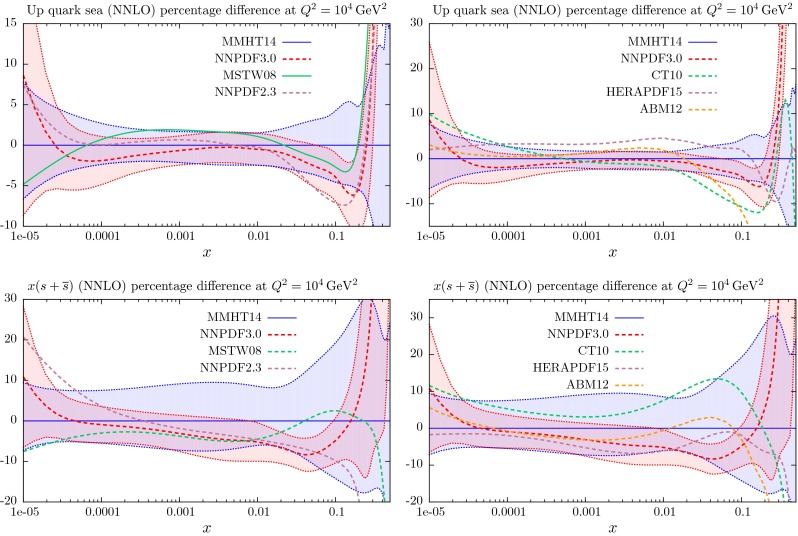
The comparison between NNLO NNPDF3.0 and MMHT14 PDFs at $Q^2=10^4~\mathrm GeV^2$ showing the $\bar{u}$ and $s + \bar{s}$ quark PDFs. Also shown (without error corridors) are the NNPDF2.3 and MSTW08 PDFs (*left*) which they supersede and (*right*) CT10 HERAPDF1.5 and ABM12 PDFs

**Fig. 32:**
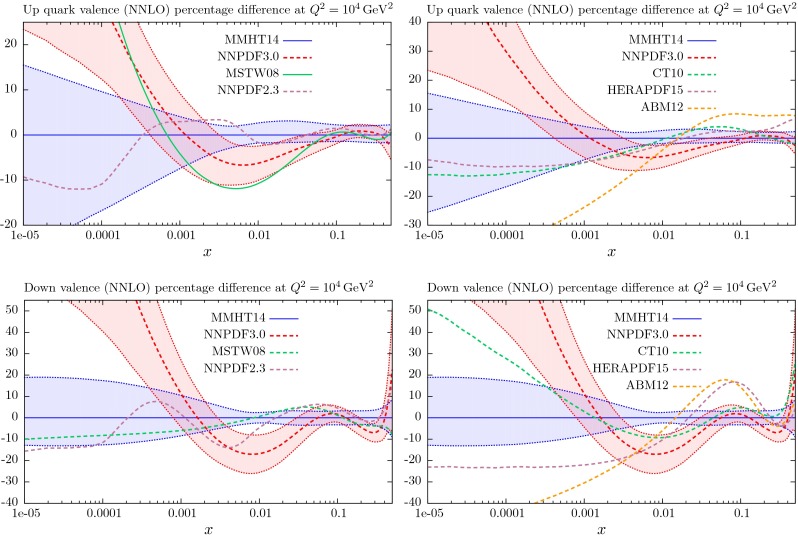
The comparison between NNLO NNPDF3.0 and MMHT14 PDFs at $Q^2=10^4~\mathrm GeV^2$ showing the $u_V$ and $d_V$ quark PDFs. Also shown (without error corridors) are the NNPDF2.3 and MSTW08 PDFs (*left*) which they supersede and (*right*) CT10 HERAPDF1.5 and ABM12 PDFs

As a representative sample, we show in Figs. 30, 31 and 32 the comparison of MMHT14 and NNPDF3.0 for six PDFs: namely the $g$, light quark, $u_V$, $d_V$, $\bar{u}$ and $s + \bar{s}$, at $Q^2=10^4~\mathrm GeV^2$ at NNLO. All the plots show the MMHT14 and NNPDF3.0 PDFs with their error corridors. The plots on the left of the figures also show the MSTW08 and NNPDF2.3 PDFs (but now without their error corridors), which have been superseded by the MMHT14 and NNPDF3.0 sets, respectively, The plots on the right of the figures show the comparison with the central values of ABM12, CT10 and HERAPDF1.5 PDFs. These representative plots of PDFs are sufficient to draw general conclusions concerning the comparisons, which we discuss in the subsections below.

As noted above, the treatment of the input distributions and the uncertainties are quite different in the NNPDF and MMHT analyses. However, remarkably, we see from Figs. 30, 31 and 32 that in regions where the NNLO PDFs are tightly constrained by the data, with a few exceptions, the values, and also the error corridors, are very consistent between the two analyses.

### Comparison of gluon PDFs and sea quark PDFs

We may conclude (at $Q^2=10^4~\mathrm GeV^2$) that to within 2 % accuracy, the NNLO gluon is determined in the domain $3\times 10^{-4} \lesssim x \lesssim 5\times 10^{-2}$. There is much better agreement between MMHT14 and NNPDF3.0 for the gluon than between MSTW08 and NNPDF2.3.^17^ In the region $x \sim 0.01$ NNPDF2.3 is outside the combined error band of the two newer sets (leading to the reduced cross section for Higgs production via gluon fusion for the NNPDF update noted in [17]). For $x \sim 0.0001$–$0.001$ MSTW08 is outside the combined error band (though quite close to NNPDF2.3).

The CT10 and HERAPDF1.5 gluons are in good agreement with MMHT14/NNPDF3.0, except for HERAPDF near $x=0.1$–$0.2$, though at the edge of the error band precisely at the central Higgs rapidity $x$ values of $0.01$–$0.02$. ABM12 is much larger below $x \sim 0.05$ and much smaller for $x>0.1$. Part of this is due to the much smaller strong coupling obtained by ABM12, but the general effect persists even if $\alpha _S(M_Z^2)=0.118$ is used. It was argued in [36] that this difference with ABM12 is primarily due to their use of a fixed-flavour number scheme (FFNS).

The very good agreement in the MMHT14 and NNPDF3.0 gluon distributions is responsible for the comparably good agreement in the small-$x$ ($x<0.01$) light-quark, $\bar{u}$ and $s + \bar{s}$ distributions, which are driven at small $x$ by evolution mainly from the gluon. For these values of $x$ the superseded MSTW08 and NNPDF2.3 distributions for these PDFs also show good agreement, although there has been a noticeable transfer from $\bar{u}$ to $s +\bar{s}$ quarks in going from MSTW08 to MMHT14. It would be surprising to see much change in the sea quarks in this region, as a linear combination of them is very tightly constrained by HERA structure function data. Indeed, there is also generally good agreement with ABM12, CT10 and HERAPDF1.5 distributions. CT10 lies a little higher at very small $x$, consistent with the similar feature for the gluon distribution. HERAPDF has a distinctly higher $\bar{u}$ distribution at lower $x$, but this is compensated, to some extent, by a smaller $s + \bar{s}$ distribution.

Perhaps the most surprising discrepancy between MMHT14 and NNPDF3.0 is in the total light-quark distribution at $x\sim 0.05$; see Fig. 30. This seems to be a particular feature of NNPDF, with NNPDF2.3 and NNPDF3.0 being very similar, while all the other PDF sets are very similar to MMHT14 in this region. The difference is $\sim 3~\%$, but the PDF uncertainty is only $\sim 1~\%$ here. The main reason for this difference seems to be that NNPDF have the smallest strange quark in this region, as well as smaller valence quarks than other PDF sets. NNPDF are the only sets of PDFs which have used HERA-II data, which constrain this $x$ range, so this may have some effect. Also, the singlet-quark distribution is probed in charged-current neutrino DIS by $F_2(x,Q^2)$, and some difference may be due to nuclear corrections being or not being included when fitting to these data. The smaller NNPDF light-quark distribution for $x \sim 0.05$ is perhaps apparent in NNPDF3.0 having smaller quark–quark luminosity than CT10 and MSTW08 in Fig. 59 of [17] for $M_X\sim 600~\mathrm GeV$ at the LHC with 13 TeV centre-of-mass energy. However, in the luminosity plot the error bands easily overlap due to sampling a range of $x$ values for each $M_X$.

### Comparison of $s+\bar{s}$ distributions

The MMHT14 and NNPDF3.0 $s + \bar{s}$ distributions are fully compatible, but NNPDF3.0 has a lower distribution. The latter observation is due to the increase in the strange fraction in MMHT14 arising from the improved treatment of the $D\rightarrow \mu$ branching ratio $B_{\mu }$, whereas NNPDF3.0 is similar to NNPDF2.3 (and also to MSTW08, except at fairly high $x$ values). The improved treatment of $B_{\mu }$ means MMHT14 has a rather larger uncertainty for $s + \bar{s}$ than previously, and this also seems to be larger than that for NNPDF3.0.

MMHT14 also has a larger total strange distribution than HERAPDF (as already noted at small $x$), but the two are compatible. There is quite good agreement with ABM12 except for $x>0.2$, where there is little constraint from data. CT10 has the largest $s+ \bar{s}$ distribution, and the central value is even outside the MMHT14 error band near $x=0.05$, though their uncertainty band is large. However, it was recently reported in [151] that a sign error was discovered in the CT10 heavy flavour contribution to charged-current DIS. This led to a considerable underestimate of the dimuon cross section, and hence a larger strange distribution. A significant reduction of $s+ \bar{s}$ is therefore expected in future CT PDF sets.

### Comparison of valence quark distributions

There is, perhaps unsurprisingly, more difference in the PDFs for valence distributions, as seen in Fig. 32, since there is less direct constraint from the data. MMHT14 and NNPDF3.0 agree well for both $u_V$ and $d_V$ at $x > 0.05$ where the valence quarks provide the dominant contribution to the structure function data. However, at lower $x$ values. Where sea quarks dominate, the PDFs start to differ significantly. Both the $u_V$ and $d_V$ of NNPDF3.0 become smaller than those of MMHT14 for $x \sim 0.01$ (though more so for $d_V$), and then become larger at very small $x$ as a result of the quark number constraint.

The same sign difference for both valence quarks for $x \sim 0.01$ allows $u_V-d_V$ to be similar for MMHT14 and NNPDF3.0, so both fit the LHC lepton asymmetry data at low rapidity, which is sensitive to $u_V-d_V$ at $x \sim 0.01$. It may be the case that the absence of deuteron corrections in NNPDF3.0 compared to the relatively large ones now used in the MMHT14 analysis leads to a difference in the $d_V$ distribution which also impacts on the $u_V$ distribution due to the constraint on the difference between them. Indeed, MSTW08 (which had a more restricted deuteron correction) and NNPDF2.3 agree quite well for $d_V$. However, there is also some direct constraint on valence distributions from nuclear target data, and also sensitivity to the $F_3(x,Q^2)$ structure function. Here MMHT apply nuclear correction factors, while NNPDF do not, and also they employ a larger $Q^2$ cut for $F_3(x,Q^2)$ than for $F_2(x,Q^2)$ due to the probable large higher-twist corrections at lower $x$ values. As already commented on, the valence distributions in MMHT14 and MSTW08 are quite different due to the extended parameterisation and to the deuteron corrections – the main features of the change are already present in [11]. Note that there are also some quite significant changes in going from NNPDF2.3 to NNPDF3.0 at smaller $x$.

The MMHT14 $u_V$ distribution agrees quite well with that of both CT10 and HERAPDF1.5. The ABM12 $u_V$ distribution is very different in shape to all the rest, perhaps due to the approach of fitting higher-twist corrections, rather than employing a *conservative* kinematic cut as the other groups do. MMHT14 also exhibits reasonable agreement with the CT10 $d_V$ distribution, but both HERAPDF1.5 and ABM12 have quite different shapes (though similar to each other). HERAPDF has little constraint on $d_V$ and the uncertainty is large, though it is not influenced by assumptions about deuteron corrections or by imposing isospin symmetry conservation. The reason for the difference for ABM12 may be similar to that proposed for the difference in $u_V$. The valence quarks are very different as $x \rightarrow 0$, perhaps suggesting an underestimation of the uncertainty here, even by NNPDF. However, it is not clear what experimental data would be sensitive to the very small $x$ valence quark differences.

### Comparison at NLO

**Fig. 33:**
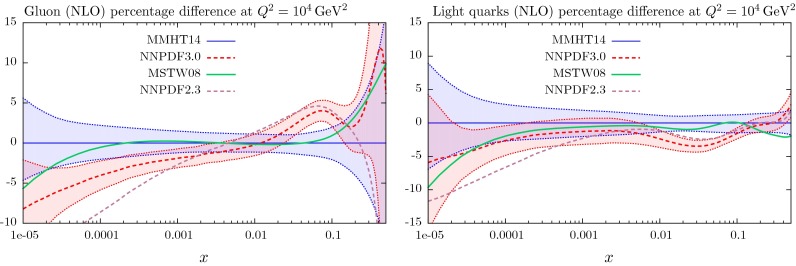
The comparison between NLO NNPDF3.0 and MMHT14 PDFs at $Q^2=10^4~\mathrm GeV^2$. The two plots show the $g$ and light-quark PDFs. Also shown (without error corridors) are the NNPDF2.3 and MSTW08 PDFs which they supersede

The same type of PDF comparison is made between NNPDF3.0 and MMHT14 at NLO in Fig. 33. For the gluon (left-hand plot) this shows less agreement between the values of the MMHT14 and NNPDF3.0 PDFs than the comparison at NNLO, though the width of the error corridors are still comparable. NNPDF3.0 is larger for $x \sim 0.1$ but becomes considerably smaller at very low $x$. Even so, the plots show that there is now closer agreement than between the MSTW08 [1] and NNPDF2.3 [3] PDFs that they supersede, though the form of the difference is the same. For the quarks the differences between PDF sets are largely similar at NLO as at NNLO (an exception being that HERAPDF1.5 has a smaller high-$x$ gluon at NLO and larger high-$x$ sea quarks compared to its NNLO comparison to other sets). The main additional difference between NNPDF3.0 and MMHT14 (and between NNPDF2.3 and MSTW08) is simply that inherited from the gluon difference, i.e. the smaller NNPDF gluon at low $x$ leads to smaller low $x$ sea quarks. This is illustrated in the NLO comparison of the light-quark distributions shown in the right-hand-side plot of Fig. 33, and is similar for all sea quarks at low $x$.

So far we have compared the PDF sets at $Q^2=10^4 ~\mathrm GeV^2$. The comparison of MMHT14 and NNPDF3.0 (and other) PDFs at lower $Q^2$, say $Q^2=10~\mathrm GeV^2$, shows the same general trends, but now the error corridors are wider, particularly at very small $x$, as illustrated for MMHT2014 PDFs in Figs. 1 and 20, respectively.

## Conclusions

We have performed fits to the available global hard scattering data to determine the PDFs of the proton at NLO and NNLO, as well as at LO. These PDF sets, denoted MMHT2014, supersede the MSTW2008 sets, that were obtained using a similar framework, since we have made improvements in the theoretical procedure and since more data have become available in the intervening period. The resulting MMHT2014 PDF sets may be accessed, as functions of $x,Q^2$ in computer retrievable form, as described in Sect. 5.3.4.

How has the theoretical framework been improved? This was the subject of Sect. 2. First, we now base the parameterisation of the input distributions on Chebyshev polynomials. It was shown in [11] that this provided a more stable determination of the parameters. We now also use more free parameters than previously, i.e. an additional two for each valence quark, for the overall sea distribution and the strange sea. However, we only use five more in determining PDF eigenvectors as there is some still some redundancy in parameters. Next, note that even with the advent of LHC data, we find we still need the fixed-target nuclear data to determine the flavour separation of the PDFs. So our second improvement is to use a physically motivated parametric form for the deuteron correction, and to allow the data to determine the parameters with the uncertainties determined by the quality of the fit. The first step in this direction was taken in [11], but now we find that the global fit results in a correction factor even more in line with theoretical expectations; see Fig. 3. There are similar improvements for the heavy-nuclear corrections for the deep inelastic neutrino scattering data, with an update of the corrections used, and again allowing some freedom to modify these corrections and for the fit to choose the final form. The third improvement concerns the treatment of the heavy $(c,b)$ quark thresholds. We use an optimal GM-VFNS to give improved smoothness in the transition region where the number of active flavours increases by one. The fourth improvement is to use the multiplicative, rather than the additive, definition of correlated uncertainties. Another important change in our procedure is the treatment of the $D\rightarrow \mu$ branching ratio, $B_{\mu }$, needed in the analysis of (anti)neutrino-produced dimuon data. These data give the primary constraints on the $s$ and $\bar{s}$ PDFs. In the present analysis we avoid using the determination of $B_{\mu }$ obtained independently from the same dimuon data, but instead, in the global fit, we include the value, and its uncertainty, obtained from direct measurements. It turns out that the global fit determines a consistent value of $B_{\mu }$, but with a larger uncertainty than the direct measurement, leading to a much larger uncertainty on the strange quark PDFs than that in the MSTW2008 PDFs; see Figs. 23 and 25.

What data are now included that were not available for the MSTW08 analysis? This was the subject of Sects. 3 and 4. First, we are now able to use the combined H1 and ZEUS run I HERA data for the neutral and charged current, and for the charm structure functions. Then we have $W$ charge asymmetry data updated from the Tevatron experiments and new from the LHC experiments. We also have LHC data for $W,Z$, top-quark-pair and jet production. It is interesting to see which data sets most constrain the PDFs. This is discussed in Sect. 5.3.3; and displayed in Tables 7 and 9 for the NLO and NNLO PDF sets, respectively. It is still the case that the constraints come from a very wide variety of data sets, both old and new, with LHC data providing some important constraints, particularly on quark flavour decomposition.

Some LHC data are not included in the present fits; namely dijet production, $W+$charm jet data and the differential top-quark-pair distributions. However, as shown in Sect. 7, these data seem to be well predicted by MMHT14 partons, except for the behaviour of $t\bar{t}$ production at large $p_T^t$ (using NLO QCD), see Sect. 7.3. In all these cases full NNLO corrections are still awaited, and it will be interesting to see how they change the predictions we have at NLO.

The new MMHT14 PDFs only significantly differ from the MSTW08 PDF sets for $u_V-d_V$ for $x\sim 0.01$; see Fig. 23. The only data probing valence quarks in this region are the $W$ charge asymmetry measurements at the Tevatron and the LHC. The MSTW08 partons gave a poor description of these data. This was cured by changing to a Chebyshev polynomial parameterisation of the input distributions, with more free parameters, and by a better treatment of the form of the deuteron corrections, as first noted in [11], and further improved here. It is therefore not surprising that the MSTW08 PDFs still give reliable predictions for all other data; see Tables 11 and 12 for some NLO and NNLO predictions, respectively. The only other significant change is in the total strange quark distribution, with a moderate increase in magnitude (larger than the MSTW2008 uncertainty) for the best-fit value, but a very significant increase in uncertainty. Thus, we may conclude that one is unlikely to obtain an inaccurate prediction for the vast majority of processes using MSTW08 PDFs, but we recommend the use of MMHT14 PDFs for the optimum accuracy for both the central value and uncertainty.

As we enter an era of precision physics at the LHC, it is crucial to have PDFs determined as precisely as possible. So improvements to the MSTW08 PDFs are valuable. In this respect, it is important to notice that the values and error corridors of the two very recent sets of PDFs (the MMHT14 and NNPDF3.0 sets, obtained with very different methodologies) are consistent with each other at NNLO, with only a few differences of more than one standard deviation, and that the values are closer together than hitherto; see Figs. 30, 31 and 32. Hence, although it appears that the intrinsic uncertainties from individual PDF sets are not shrinking at present, with new data being balanced by better means of estimating full PDF uncertainty, the PDF uncertainties from combinations of PDFs, for example as in [130], are very likely to decrease in the future.

We note that the current strategy is to upgrade and to run the LHC at $\sqrt{s}= 14$ TeV, with increasing integrated luminosity from 30 fb$^{-1}$ (already taken at $\sqrt{s}=8$ TeV) to 300 fb$^{-1}$ at the first stage, and eventually, in the High Luminosity LHC (HL-LHC), to 3000 fb$^{-1}$ [152]. The increase in luminosity means that we can increase the mass reach for the direct search of new particles. For example, the last factor of 10 gain in luminosity means the centre-of-mass energy reach goes from about 7.5 to 8.5 TeV [152], while HL-LHC continues to operate at $\sqrt{s}=14$ TeV. However, the knowledge of the PDFs at large $x$ will also have to improve. From the present study, we see that gluon PDF at NNLO at $Q^2=10^4~\mathrm GeV^2$ is known to within a small number of $\%$ for $0.001\lesssim x \lesssim 0.2$, but that, at the moment, we have little constraint from the data in the larger $x$ domain. For the two processes which constrain the high $x$ gluon PDF, that is, jet production and the differential distributions for top-quark-pair production, it will be important to complete the NNLO formalism. There are already some results for the former process in [115–117] and for the latter process in [153]. On the experimental side it will be important to reliably measure the distributions for these processes, particularly for values of $p^t_T$ and rapidity $y_t$, which are as large as possible.

## Footnotes

1 We note that preliminary reports on these new PDFs have been presented in [7, 8].

2 The PDF sets in this article are often referred to as MSTWCPdeut, but we will use the nomenclature MMSTWW, i.e. the initials of the authors of the article, throughout this paper.

3 This choice works well for PDF uncertainties, as discussed in [26].

4 We do not treat the top quark as a parton, i.e. even at high scale we remain in a five flavour scheme. Even at LHC energies the mass of the top quark is quite large compared to any other scale in the process, and the expressions for the cross sections for top production are all available in the scheme where the top appears in the final state.

5 The parameters are those of the input PDFs, the QCD coupling $\alpha _s(M_Z^2)$ and the nuclear corrections.

6 Exceptions are exclusive $J/\psi$ production [50] and low-mass Drell–Yan production [51] at high rapidity $y$ at the LHC, but here the data are sparse and, moreover, on the theory side, there are potentially large uncertainties, particularly in the former case where it is not the standard integrated PDFs which are being directly probed, and more work is needed for data from these processes to be useful [52–56].

7 We note that the measurement at 8 TeV is actually published after the beginning of 2014 (although submitted at the end of 2013), and hence officially does not satisfy our cut-off on the date for data included. However, this data point is extremely well fit at both NLO and NNLO, with the contribution to the $\chi ^2$ much less than 1 unit, and has extremely little pull on the PDFs. It is effectively included as a comparison rather than as a constraint.

8 In the analysis of [109] we cut two more ATLAS points at the edge of rapidity bins due to very poor fits to these points. This was much more of an issue when using the additive definition for correlated uncertainties, and we have reinstated these points here. Indeed the whole fit quality for this data set is much better using the multiplicative definition.

9 It has very recently been brought to our attention that there is a change in the luminosity determination for the data in [10, 107], and the cross sections should be multiplied by a factor of 1.0187 and the uncertainty on the global normalisation (“Lumi”) increases slightly from $3.4$ to $3.5\,\%$. This was too late to be included explicitly in our PDF determination. However, we note that this corrections results in the $\chi ^2$ for the best fits at NLO and NNLO both reducing by about half a unit, and any changes in the PDFs are very much smaller than all uncertainties.

10 The dependence on $R$ was not accounted for in [120].

11 We realise that, strictly speaking, the D0 jet data are difficult to include in an NNLO fit since the mid-point algorithm used becomes infrared unsafe at this order [123]. However, the whole “NNLO” jet treatment is approximate at present. We will revisit the question of whether to include these data in future fits when the full NNLO calculation is known. At this time presumably there will also be more precise LHC jet data and the D0 jet data would play a diminishing role in the fit anyway.

12 We note, however, that the stability of the fit quality to CMS jet data with inclusion of NNLO $K$-factors was less apparent before the improved treatment of systematics advocated in [114] was incorporated, and a fit with the data included did tend to lower $\alpha _S(M_Z^2)$ slightly.

13 The expressions for the input PDFs in terms of the parameters are given in Sect. 2.1.

14 See [134], where it is shown this is equivalent to treating $\alpha _S(M_Z^2)$ as an extra parameter in the eigenvector approach in the limit that the Hessian formalism is working perfectly.

15 We use the code provided by Stirling, based on the calculation in [137, 138] and [139].

16 The ABM12 analysis does include some of the LHC $W,Z$ data.

17 We note that NNPDF3.0 uses a charm pole mass of $m_c=1.275~\mathrm GeV$ rather than the value $m_c=\sqrt{2}~\mathrm GeV$ used for NNPDF2.3. As noted in [149] (see Fig. 4), and [150] (see Fig. 40) this type of change has some effect on the gluon, potentially of order $1~\%$ at $Q^2=10^4~\mathrm GeV^2$ (except at very high and low $x$), but very little change near $x=0.01$. The value of $m_b$ is also changed, but this should have negligible change on the PDFs, except for the $b$ distribution.
